# Harnessing Functionalized Thioalkylated‐Cyclopentadithiophene Monolayers on NiOx for Two‐Step Fabricated Tin Perovskite Solar Cells

**DOI:** 10.1002/smtd.202501309

**Published:** 2025-09-03

**Authors:** Shakil N. Afraj, Yun‐Sheng Shih, Che‐Hsin Kuo, Chun‐Hsiao Kuan, Pei‐Yu Huang, Pei‐Yun Lee, Arulmozhi Velusamy, Shao‐Huan Hong, Cheng‐Liang Liu, Xianyuan Jiang, Ming‐Chou Chen, Eric Wei‐Guang Diau

**Affiliations:** ^1^ Department of Chemistry National Central University No. 300 Zhongda Rd. Taoyuan Taiwan 320317 China; ^2^ Department of Applied Chemistry and Institute of Molecular Science National Yang Ming Chiao Tung University 1001 Ta‐Hseuh Rd. Hsinchu Taiwan 300093 China; ^3^ Center for Emergent Functional Matter Science National Yang Ming Chiao Tung University 1001 Ta‐Hseuh Rd. Hsinchu Taiwan 300093 China; ^4^ Department of Materials Science and Engineering National Taiwan University No. 1, Sec. 4, Roosevelt Rd. Taipei Taiwan 106319 China; ^5^ School of Physical Science and Technology ShanghaiTech University 393 Middle Huaxia Road Pudong New Area Shanghai 201210 China

**Keywords:** cyclopentadithiophene, dithioalkylated, high‐efficiency, self‐assembled monolayers, stable TPSC, two‐step fabrication

## Abstract

A new series of dithioalkylated‐methylidenyl‐cyclopentadithiophene (CDTS)‐based self‐assembled monolayers (SAMs), including **CDTS‐MN** (**1**), **CDTS^b^‐MN** (**1b**), **CDTS‐CA** (**2**), and **CDTS‐PA** (**3**), are developed for tin perovskite solar cells (TPSCs). Each SAM incorporates sulphur rich center CDTS and is complemented with various anchoring groups, such as methylenemalononitrile, cyanoacrylic acid, and cyano‐vinyl‐phosphonic acid in driving the formation of well‐crystallized and homogeneous perovskite layers using a two‐step fabrication process. Nickel Oxide (NiOx) combined with the newly designed CDTS‐based SAMs is utilized as the hole transport material (HTM). This configuration yielded an excellent power conversion efficiency (PCE) of 8.41% for **CDTS‐MN** (**1**) TPSC device as compared to **CDTS^b^‐MN** (**1b**; PCE: 8.03%), **CDTS‐CA** (**2**; PCE: 4.54%) and **CDTS‐PA** (**3**; PCE: 5.15%). Notably, single‐crystal structures of **CDTS‐MN (1)** and **CDTS^b^‐MN** (**1b**) are successfully obtained and systematically presented, demonstrating the uniformity of the CDTS monolayers formed on the ITO/NiOx substrate. The CDTS‐based SAM **1** excels in TPSCs by enhancing charge transfer and reducing interface recombination through strong π–π stacking and intermolecular S—S interactions. This report highlights the pioneering use of CDTS‐based organic sensitizers in TPSCs, representing the first documented application of CDTS‐based SAMs within the solar cell research field.

## Graphical abstract

A new series of CDTS‐based self‐assembled monolayers on NiOx is developed, which enabled a high‐performance tin‐based perovskite solar cell with an efficiency of 8.4%. With strong intramolecular S···S interactions, the CDTS motif enhanced charge transport, improved efficiency, and gave excellent long‐term stability.

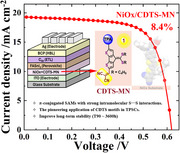

## 1 Introduction

As global population growth accelerates, the demand for energy continues to rise, highlighting the importance of efficient solar‐to‐electricity conversion.^[^ ^1^ ^]^ Solar cells present a sustainable solution to address increasing energy needs while reducing environmental impacts.^[^ ^2^, ^3^, ^4^, ^5^, ^6^ ^]^ At present perovskite solar cells (PSCs) are gaining prominence in research because of their numerous advantages such as affordable solution processing, adjustable band gaps, and extended exciton diffusion lengths.^[^ ^7^, ^8^, ^9^ ^]^ This makes them one of the most promising solar cell technologies on the market.^[^ ^10^ ^]^ The highest PCE of Pb‐based PSCs currently reaches 26.7%, comparable to those of silicon solar cells.^[^ ^11^ ^]^ However, the toxicity of lead in these cells raises significant environmental concerns, posing risks during manufacturing, use, and disposal.^[^ ^12^ ^]^ Conversely, tin‐based PSCs (TPSCs) provide a safer alternative, presenting a more environmentally friendly option.

Notably, TPSCs demonstrate an ideal optical band gap, exceptional carrier mobility, and outstanding optoelectronic properties, making them significant for advanced applications in optoelectronic and photovoltaic fields.^[^ ^13^ ^]^ The significant improvements in power conversion efficiency (PCE) reaching 17.13%^[^ ^14^ ^]^ for TPSC have established them as strong contenders for green photovoltaic technologies.^[^ ^15^, ^16^, ^17^ ^]^ However, TPSC devices face considerable open‐circuit voltage (*V*_OC_) deficits caused by the spontaneous oxidation of Sn^2+^ and an uncontrolled crystallization process.^[^ ^18^ ^]^ To advance the TPSC technology, various strategies have been developed to inhibit Sn^2+^ oxidation, stabilize surface defects, and manage perovskite crystal growth. Traditionally, well‐known HTM, poly(3,4‐ethylenedioxythiophene):poly(styrenesulfonate)(PEDOT:PSS) has been used in a single‐step deposition process.^[^ ^19^, ^20^, ^21^ ^]^ However, its hygroscopic nature can degrade performance in humid conditions. To overcome this problem, a two‐step deposition technique has been introduced, allowing compatibility with hydrophobic polymeric HTMs like poly[bis(4‐phenyl)(2,4,6‐trimethylphenyl)amine](PTAA),^[^ ^22^, ^23^, ^24^ ^]^ enhancing efficiency and stability of TPSC.^[^ ^25^ ^]^ Interestingly, researchers recently explored replacing PEDOT:PSS with small molecular organic‐self‐assembled monolayers (SAMs) to improve the interface between the perovskite and transport layers, potentially increasing the *V*_OC_.^[^ ^26^ ^]^

Ideally SAMs are defined as highly organized 2D materials that are one or a few layers thick. Typically, SAMs consist of three components: an anchoring group, spacer group, and a terminal group.^[^ ^27^, ^28^, ^29^ ^]^ The use of SAMs has significantly improved the performance of Pb‐PSCs, providing considerable benefits compared to conventional configuration.^[^ ^30^ ^]^ SAMs generate a dipole moment at the interface through their chemical bonding, which alters the energy barrier and enhances the extraction of photogenerated holes from the perovskite layer. Moreover, the capacity to precisely adjust the molecular polarity of SAMs enhances the wettability of the perovskite precursor, resulting in the formation of higher‐quality perovskite films.^[^ ^31^ ^]^ Furthermore, SAMs show great promise for the scalable production of large‐area solar modules.^[^ ^32^ ^]^ In short, when compared to traditional polymeric HTMs such as PEDOT:PSS and PTAA, SAMs offer benefits including reduced optical and electrical losses, consistent coatings, tunable work functions, straightforward interfacial modification, cost‐effective processing, and enhanced moisture resistance.^[^ ^28^, ^33^, ^34^ ^]^ Currently, PEDOT:PSS accounts for ≈70% of all published studies, reflecting the scarcity of alternative HTMs in TPSCs.^[^ ^18^ ^]^ This underscores the importance of gaining insights from the effective application of SAMs as HTMs in TPSC devices. Exploring these alternatives is essential for diversifying the HTM landscape and enhancing the performance of TPSC technologies.

Inorganic nickel oxide (NiOx) is a cost‐effective HTM for p‐i‐n PSCs, exhibiting remarkable stability and efficiency.^[^ ^35^ ^]^ However, PSCs that utilize NiOx HTMs often display lower open‐circuit voltages compared to those employing conventional HTMs such as PEDOT:PSS.^[^ ^36^ ^]^ To improve the properties of the HTM and optimize the interface between NiOx and the perovskite layers, Zhu et al. presented a new SAM called **2PADBC** as an interfacial modifier.^[^ ^37^ ^]^ Their findings revealed that the highly reactive Ni^≥3+^ defects were responsible for decreasing the electron density of Sn^2+^, resulting in Sn oxidation. The introduction of **2PADCB** effectively passivated these reactive Ni defects and increased the activation energy required for Sn oxidation. Additionally, the **2PADCB**‐modified HTM demonstrated a uniform surface potential, reduced contact potential differences, and an elevated work function. Consequently, the application of **2PADCB** led to a substantial improvement in *V*_OC_, increasing from 712 mV to 825 mV, and achieving a PCE of 14.19% for TPSCs. This example underscores the potential for designing and synthesizing various organic SAMs for further advancements in TPSC technology.

To achieve high‐quality tin perovskite films with superior morphology and optoelectronic properties, we adopted a two‐step spin‐coating technique instead of the conventional one‐step method. While the one‐step approach is convenient, it suffers from rapid crystallization, leading to poor film uniformity and suboptimal device performance. In contrast, the two‐step process allows precise control over film formation by separating precursor deposition and crystallization. A notable innovation in our method is the use of a cosolvent mixture—developed in 2020 by our lab—comprising isopropyl alcohol (IPA), hexafluoro‐2‐propanol (HFP), and chlorobenzene, which overcomes the degradation issues associated with pure IPA.^[^ ^38^ ^]^ This formulation enables the formation of dense, uniform, and pinhole‐free tin perovskite films with improved crystallinity and performance. Our attempts to use the one‐step process on SAM‐modified substrates yielded poor coverage and significant defects, further emphasizing the importance of the two‐step spin‐coating approach for fabricating high‐quality TPSCs.

Tailoring various organic‐SAMs with diverse anchoring groups specifically for TPSCs applications offers exciting potential for performance enhancement. This customization can lead to increased photovoltaic parameters, and overall PCE in TPSCs.^[^ ^39^ ^]^ In PSCs, SAMs typically form on metal oxide substrates, including ITO, SnO_2_, TiO_2_, ZnO, and NiOx, via anchoring groups.^[^ ^40^, ^41^ ^]^ Figure S1 (Supporting Information) illustrates recently published SAMs for lead‐ and tin‐based PSCs, with the primary objective of demonstrating how these SAMs with potential anchoring groups are advancing Pb and Sn‐PSCs technologies.^[^ ^25^, ^42^, ^43^, ^44^, ^45^, ^46^, ^47^, ^48^, ^49^, ^50^ ^]^

A fused‐ring cyclopentadithiophene (CDT) unit has gained substantial interest in the creation of small molecules^[^ ^51^ ^]^ and donor–acceptor polymers.^[^ ^52^ ^]^ This is largely because of its efficient electron‐donating abilities, the straightforward synthesis using readily available reagents, and the simple side‐chain modifications that increase solubility.^[^ ^53^ ^]^ Further, functionalization of CDT to the sulphur‐rich dithioalkylated methylidenyl‐CDT backbone, is attracting attention for its improved planarity, which facilitates enhanced π–π stacking and stronger S—S intermolecular interactions.^[^ ^54^, ^55^, ^56^ ^]^ Leveraging these advantageous features, our team has recently developed dithioalkylated‐methylidenyl‐CDT based compounds for efficient n‐type OFETs.^[^ ^57^ ^]^

**Figure 1:**
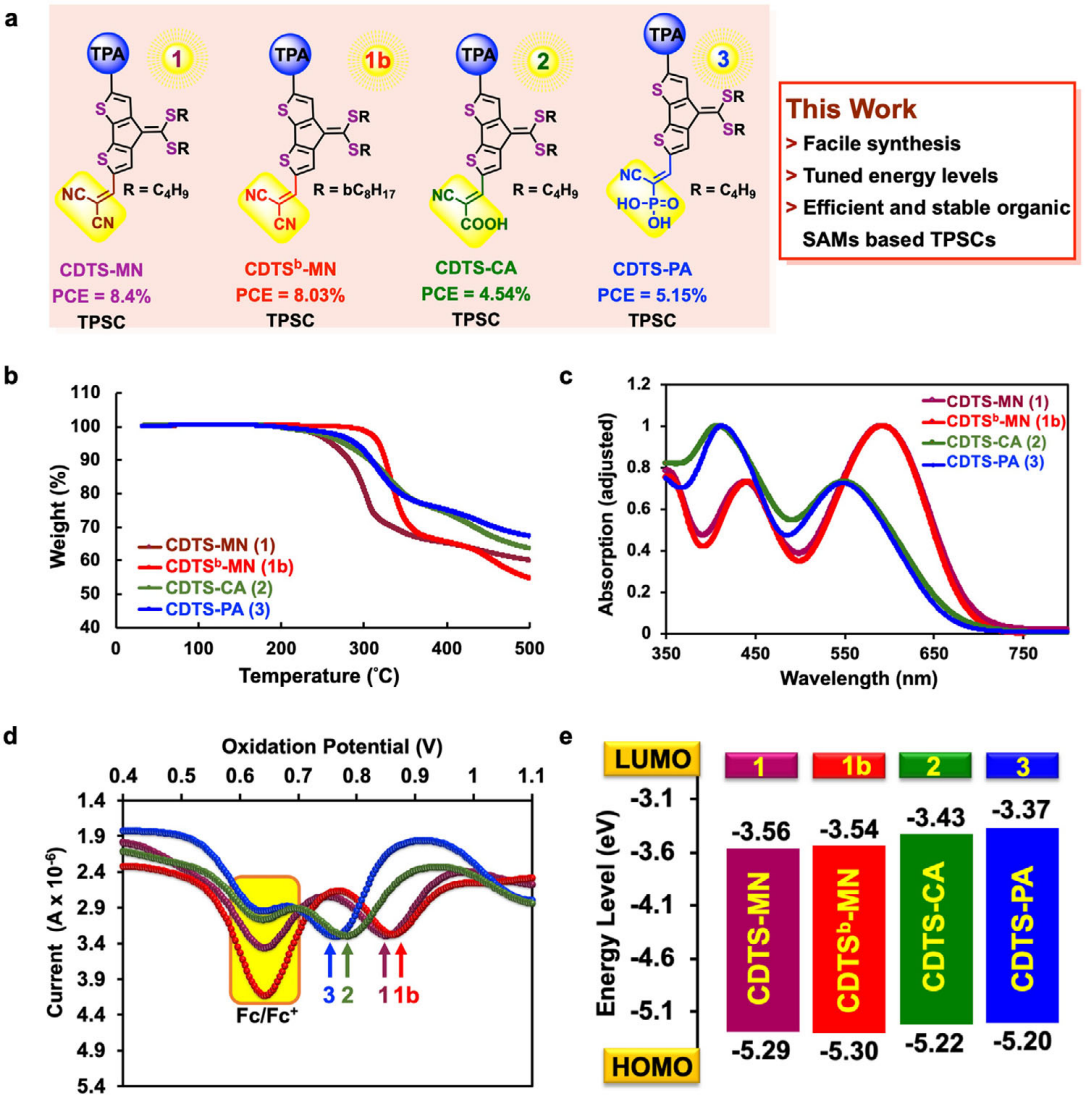
a) CDTS‐based SAM molecules **1‐3** examined in this study for TPSC application. b) Thermogravimetric analysis (TGA) curves of CDTS derivatives with 5% weight loss temperatures reported in the text. c) UV–vis absorption spectra of molecules **1–3** in *o*‐dichlorobenzene. d) Differential pulse voltammetry (DPV) profiles of molecules **1–3**. e) Energy level diagram of molecules **1–3** derived from experimental data.

Inspired by the findings mentioned above, herein, we synthesized SAMs such as **CDTS‐MN** (**1**), **CDTS^b^‐MN** (**1b**), **CDTS‐CA** (**2**), and **CDTS‐PA** (**3**), all based on a dithioalkylated methylidenyl‐CDT backbone linked to triphenylamine units and various potential anchoring groups (**Figure** **1**a). In terms of the design approach for the new CDTS‐based SAMs, the addition of dicyanomethylene, cyanoacrylic acid, and phosphonic acid groups at one terminal of the CDTS core, along with the donor unit 4,4′‐dimethoxytriphenylamine at the opposite terminal, this D‐A system results in a considerable increase in anchoring strength on the NiOx/ITO surface. This design strategy leads to improved interaction of SAMs with NiOx/ITO surface and the perovskite layer, supported by the single‐crystal structures analysis of **CDTS‐MN** and **CDTS^b^‐MN** SAMs. In addition, the dithioalkylated methylidenyl side chains in these SAMs improve solubility of organic material in common organic solvents, which should facilitate seamless integration into the SAM formation process, ensuring uniform coverage across NiOx/ITO surface. The thermal, optical, and electrochemical properties of the new SAMs **1‐3** have been thoroughly investigated and compared, alongside a comprehensive analysis of the DFT‐derived energy levels and ESP mapping to understand the distribution of high electron density within the designed structures. Consequently, our subsequent experiments revealed that using NiOx as the HTM in TPSCs resulted in a very low PCE of 4.0%. To enhance the efficiency of the TPSCs and optimize the interface between the NiOx surface and the perovskite layer. We explored combinations of the new organic **SAMs 1‐3** with the NiOx HTM. The TPSC devices based on NiOx/**CDTS‐MN**, NiOx/**CDTS^b^‐MN**, NiOx/**CDTS‐CA**, and NiOx/**CDTS‐PA** displayed PCEs of 8.41%, 8.03%, 4.54%, and 5.15%, respectively. This report showcases the innovative deployment of CDTS‐based organic sensitizers in TPSCs, marking the inaugural reported use of CDTS‐based SAMs in the field of solar cell technology.

## 2 Results and Discussion

### 2.1 Structural and Optoelectronic Properties of CDTS‐Based SAM Molecules

A series of CDT‐based SAM molecules, including **CDTS‐MN**, **CDTS‐CA**, and others, were synthesized through a concise three‐step synthetic route. The complete synthetic procedures, along with detailed characterization data, are outlined in the Experimental Section and further elaborated in the Supporting Information. Notably, single crystals of both **CDTS‐MN** and **CDTS^b^‐MN** were successfully obtained, enabling high‐resolution structural elucidation via single‐crystal X‐ray diffraction. The crystallographic data and corresponding structural models are presented in Table S1 and Figures S2–S4 (Supporting Information). To confirm the molecular structures of all final SAM compounds, comprehensive spectroscopic analyses were conducted, including high‐resolution mass spectrometry as well as ^1^H, ^13^C, and ^31^P nuclear magnetic resonance (NMR) spectroscopy. The full spectra and interpretations are provided in Figures S5–S29 (Supporting Information), offering robust validation of the designed molecular frameworks. The DFT analysis is also employed for the CDTS‐SAM and displayed in Figure S30 (Supporting Information). We studied the thermal, optical, and electrochemical properties of these self‐assembled monolayers. A compilation of the physical properties of CDTS‐based SAMs **1**‐**3** is presented in Table S2 (Supporting Information). Thermal analysis of the novel SAM molecules was performed using thermogravimetric analysis (TGA), as depicted in Figure 1b. The TGA curves indicated that all four molecules display remarkable thermal stability, with ≈5% weight loss occurring at temperatures of 254 °C, 316 °C, 260 and 268 °C for molecules **1**‐**3**, respectively. All these SAMs exhibit high *T_d_* due to the dithioalkylated‐methylidenyl‐CDT backbone, which offers improved planarity. This planarity enhances π–π stacking and strengthens S—S intermolecular interactions, allowing for closer molecular packing and stronger interactions.^[^ ^57^ ^]^ Consequently, these factors enable the organic materials to better withstand thermal stress, leading to higher thermal stability.

Figure 1c presents the UV–vis absorption spectra of SAMs **1**–**3**. The absorption spectra of molecules **CDTS‐MN** (**1**) and **CDTS^b^‐MN** (**1b**) in *o*‐C_6_H_4_Cl_2_ exhibit a significant red shift, with *λ*_max_ values of 593 and 592 nm, respectively. In contrast, **CDTS‐CA** (**2**) shows an absorption at *λ*_max_ = 550 nm, while **CDTS‐PA** (**3**) has a *λ*_max_ of 548 nm. This red shift suggests that the incorporation of a polar, electron‐deficient dicyano‐functionality in molecules **1** and **1b** enhances conjugation significantly compared to SAMs **2** and **3**.^[^ ^50^ ^]^ Differential pulse voltammetry (DPV) was conducted in *o*‐DCB at 25 °C to assess the electrochemical properties of SAM molecules **1**‐**3** (see Figure 1d,e). Tetrabutylammonium hexafluorophosphate was utilized as the electrolyte, while ferrocene acted as an internal standard to calibrate the oxidation potentials of the SAMs, with a reference potential set at +0.64 V.^[^ ^58^ ^]^ The equation E_HOMO_ = −(4.44 + *E*_ox_) was used to calculate the HOMO energy levels of SAM molecules **1‐3**. The oxidation peaks of molecules **1**‐**3** are located ≈0.85, 0.86, 0.78, and 0.70 V, respectively, resulting in E_HOMO_ values of −5.29 (**1**), −5.30 (**1b**), −5.22 (**2**), and −5.20 eV (**3**), respectively. The HOMO energy levels of cyano‐based SAMs −5.29 eV (**1**) (−5.30 eV (**1b**)), cyanoacrylic acid‐based SAMs (−5.22 eV (**2**)), and phosphonic acid‐based SAM (−5.20 eV (**3**)) illustrate the differing electronic effects of these functional groups. The cyano group, being a strong electron‐withdrawing moiety, results in the differing HOMO level due to its higher oxidation potential. In contrast, cyanoacrylic acid, while still containing the cyano group, exhibits a slightly deeper HOMO level due to the presence of the acrylic moiety, which can provide the resonance stabilization. Phosphonic acid‐based SAMs, with their effective electron‐donating capabilities through lone pairs on oxygen, yield the deeper HOMO level among these SAM series. This trend highlights how the varying electronic characteristics of these functional groups impact the electrochemical properties of the respective SAMs.^[^ ^46^, ^47^, ^49^ ^]^ The LUMO variables are calculated by adding the HOMO and bandgap values (LUMO = HOMO + *E*_g_). Molecules **1**–**3** have estimated LUMO values of −3.54 (**1**), −3.56 (**1b**), −3.43 (**2**), and −3.37 (**3**) eV respectively (Figure 1e).

### 2.2 Perovskite Quality Examination

The morphological and structural integrity of tin perovskite films is a key determinant of both the fill factor and overall PCE in photovoltaic devices. Attaining high‐performance devices necessitates the fabrication of high‐quality, uniform perovskite films with minimal defects and optimal surface properties. In this investigation, we conducted a comprehensive analysis of the surface roughness, potential, and morphology of various tin perovskite films through Kelvin Probe Force Microscopy (KPFM), Atomic Force Microscopy (AFM), and Scanning Electron Microscopy (SEM). These measurements were undertaken to better rigorously elucidate the influence exerted by disparate SAMs on the nuances of film formation.

**Figure 2:**
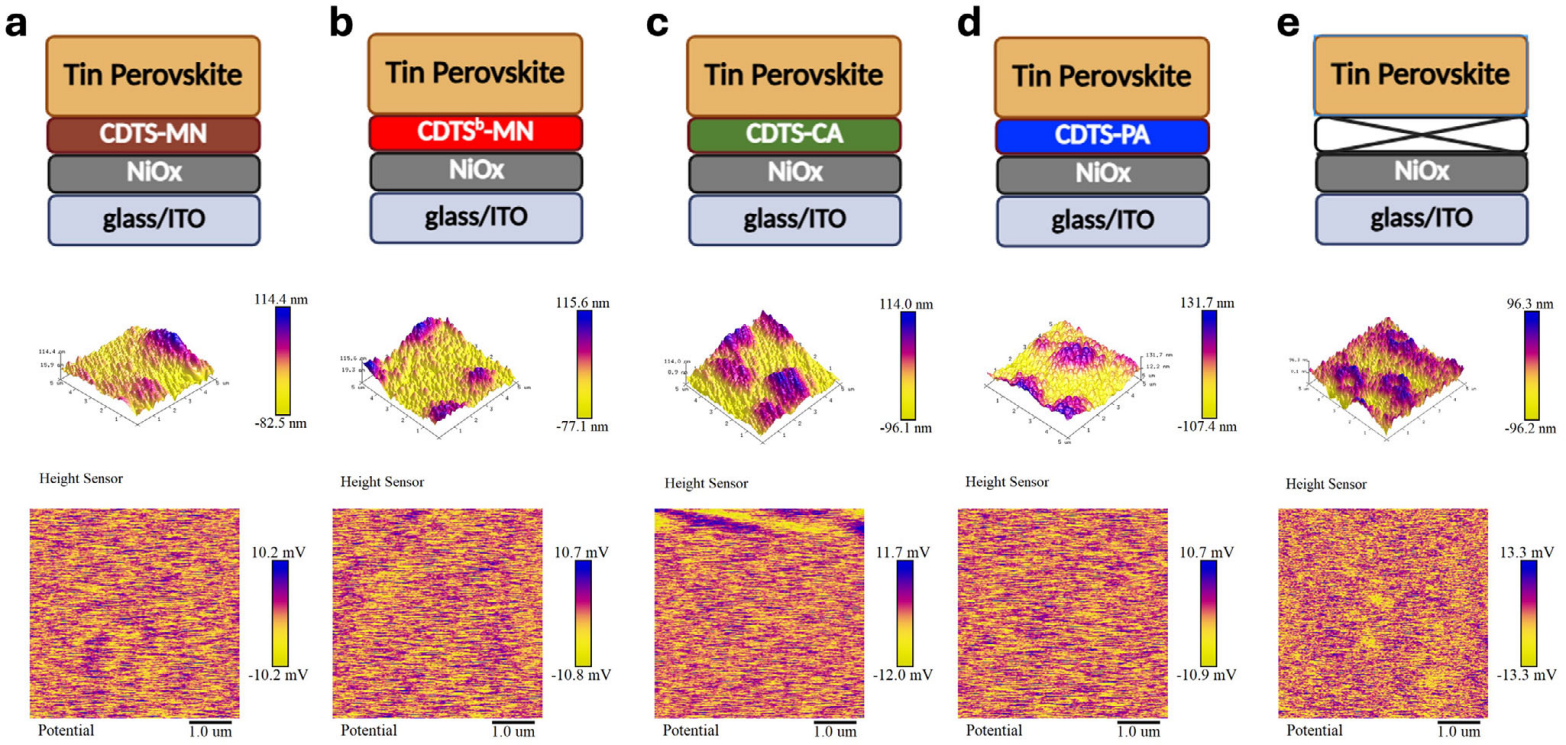
AFM and KPFM images of tin perovskite films deposited on a) **NiOx/CDTS‐MN** b) **NiOx/CDTS^b^‐MN** c) **NiOx/CDTS‐CA** d) **NiOx/CDTS‐PA** and e) **NiOx**‐only substrates.

The AFM was employed to evaluate the topography of the NiOx substrates modified with different SAMs—**CDTS‐MN**, **CDTS^b^‐MN**, **CDTS‐PA**, **CDTS‐CA**—as well as a NiOx‐only control film. As illustrated in **Figure** **2** (3D) and Figure S31 (Supporting Information) (2D), the roughness values of the SAM‐modified NiOx/ITO films were measured to be 12.5 nm for **NiOx/CDTS‐MN**, 14.3 nm for **NiOx/CDTS^b^‐MN**, 16.8 nm for **NiOx/CDTS‐PA**, and 18.6 nm for **NiOx/CDTS‐CA**, while the unmodified NiOx/ITO film exhibited a considerably rougher surface of 21.8 nm. These results clearly indicate that **NiOx/CDTS‐MN** delivers a smoother surface profile, which can facilitate the formation of a more uniform and defect‐free perovskite layer. Interestingly, the morphological evolution of the tin perovskite layer is strongly influenced by the nature of the underlying substrate. While perovskite films grown directly on SAM‐functionalized ITO substrates tend to adopt a sheet‐like structure, the incorporation of NiOx as a buffer layer transforms the morphology into a more spherical, ball‐shaped configuration. This transition results in a larger specific surface area, which enhances the interfacial contact between the NiOx/ITO and the perovskite layers, thereby improving film coverage and mechanical stability. To further evaluate the charge‐transport potential imparted by the SAMs, as shown in Figure S32 (Supporting Information), KPFM on bare NiOx/SAM substrates reveals downward conductive‐potential‐difference shifts relative to pristine NiOx, verifying SAM‐induced work‐function tuning that establishes an energetically favorable, hole‐selective surface. Building upon this interfacial modulation, we quantified the surface potential roughness of the subsequently deposited perovskite layers as presented in Figure 2. Perovskites grown on **NiOx/CDTS‐MN**, **NiOx/CDTS^b^‐MN**, **NiOx/CDTS‐CA**, and **NiOx/CDTS‐PA** exhibit surface potential roughness values of 2.31, 2.42, 2.57 and 2.44 mV, respectively, markedly lower than the 3.02 mV recorded for the reference NiOx sample. The lower surface potential roughness indicates more homogeneous band alignment and a reduced density of interfacial traps, which collectively enable efficient hole transport while suppressing interfacial electron accumulation.

As shown in Figure S33 (Supporting Information), the perovskite fabricated using **CDTS‐MN** demonstrates distinct advantages in terms of crystallinity, featuring significantly larger grain sizes and more cohesive grain boundaries compared to other samples. Such morphological characteristics are emblematic of augmented film formation and a diminished trap‐state density, both of which are indispensable for facilitating efficient charge transport. The cross‐sectional SEM image in Figure S34 (Supporting Information) reveals that the perovskite layer created with **CDTS‐MN** exhibits a greater thickness (≈341 nm) than the films prepared with other SAMs in the CDTS‐based series. This increased thickness potentially supports improved light harvesting, contributing to higher current densities and overall device efficiency. Surface roughness plays a pivotal role in the interfacial quality between the perovskite and electron transport layers.

The primary factor affecting the connection between the tin perovskite and the hole transport layer is the contact angle of the precursor, determining their interfacial adhesion. SAMs do not, however, invariably have hydrophilic qualities. To address this limitation, we incorporated NiOx to enhance SAMs hydrophilicity. Figure S35 (Supporting Information) presents the contact angles of tin perovskite precursor solutions on SAM/NiOx films, comparing those of SAMs lacking NiOx. After using NiOx, the contact angles for **CDTS‐MN**, **CDTS^b^‐MN**, **CDTS‐PA**, and **CDTS‐CA** were measured to be 10.95°, 12.35°, 18.11°, and 18.88°, respectively, as shown in Figure S36 (Supporting Information). In contrast, the NiOx‐only film exhibited a significantly higher contact angle of 23.2° (Figure S36, Supporting Information). NiOx's assistance with improving surface hydrophilicity is confirmed by the observed decrease in contact angles. Furthermore, a reduced contact angle during the two‐step fabrication process encourages the generation of SnI_2_ films, which in turn affects FAI deposition and makes it easier to create high‐quality FASnI_3_ film. Better perovskite film quality and increased device efficiency are the results of this advancement.

### 2.3 Electronic Properties of Tin Perovskite

**Figure 3:**
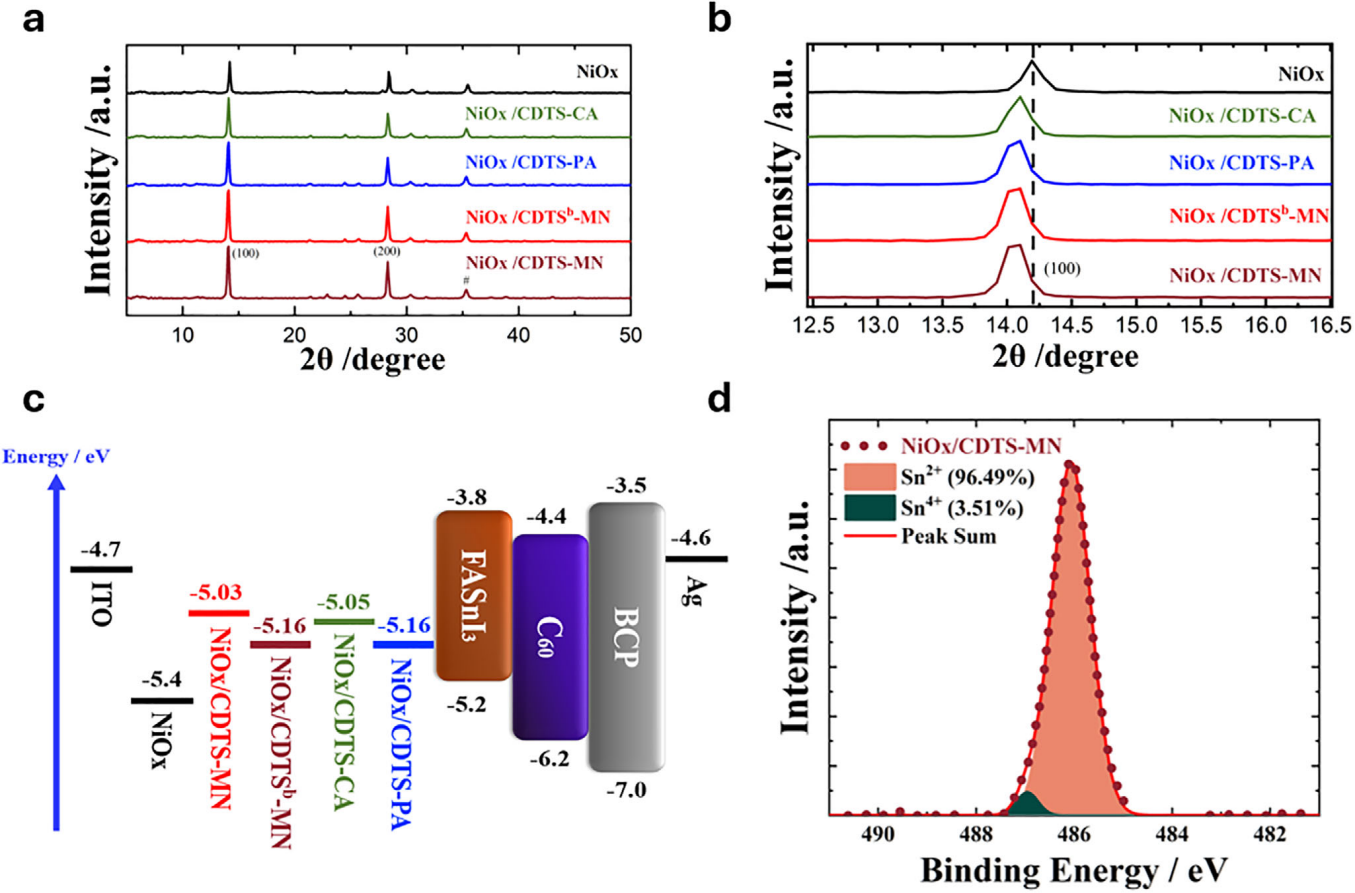
a) XRD patterns of tin perovskite films on NiOx modified with different SAMs. b) Peak shifts of the (100) diffraction plane. c) Energy‐level alignment of device components. d) XPS spectra of tin perovskite films formed on NiOx/CDTS‐MN.

The crystallinity of the tin perovskite is vital for the solar cell performance. The X‐ray diffraction (XRD) results of the CDTS‐based tin perovskite are displayed in **Figure** **3**a. We find out that the primary peak at 14° has different XRD intensities, this peak corresponding to the (100) plane signal. Among the samples, **NiOx/CDTS‐MN** exhibits the highest peak intensity, suggesting superior film quality compared to the other tin perovskites. Notably, the alteration of SAM results in the generation of tensile strain in the perovskite films because its thermal expansion constant is lower than that of NiOx. Finally, as seen in Figure 3b, the perovskite (100) peaks were shifted to smaller angles, indicating expansion of the crystal units.

The detailed energy level alignment across these layers, as determined by ultraviolet photoelectron spectroscopy (UPS), is illustrated in Figure 3c, providing insights into the interfacial energy offsets and facilitating an understanding of charge carrier dynamics within the device. Figures S37 and S38 (Supporting Information) exhibit the UPS raw data with and without NiOx. The persistent challenge of tin oxidation in tin perovskite necessitates innovative solutions. Note that the slight variations in the trends of energy levels observed among the DFT, DPV, and UPS measurements are not unexpected and are probably due to the distinct environmental conditions in which the measurements or calculations were conducted.^[^ ^58^, ^59^ ^]^ To mitigate this impediment, we have substituted PEDOT: PSS with a SAM and NiOx. Simultaneously, x‐ray photoelectron spectroscopy (XPS) was used to investigate the oxidation state of tin in perovskite films incorporating CDTS‐series SAMs. The Sn^2^⁺ ratio was determined to be 96.49% for **CDTS‐MN**, 95.18% for **CDTS^b^‐MN**, 94.48% for **CDTS‐PA**, and 93.68% for **CDTS‐CA**. Notably, **CDTS‐MN** exhibited the highest Sn^2^⁺/Sn⁴⁺ ratio, as illustrated in Figure 3d and Figure S39 (Supporting Information). The empirical evidence exhibits the profound efficacy of the NiOx/SAM bilayer in attenuating the tin oxidation within the perovskite layer. The XPS results are consistent with the device performances, showing the same trend for this series of SAM devices.

### 2.4 Carrier Dynamics

**Figure 4:**
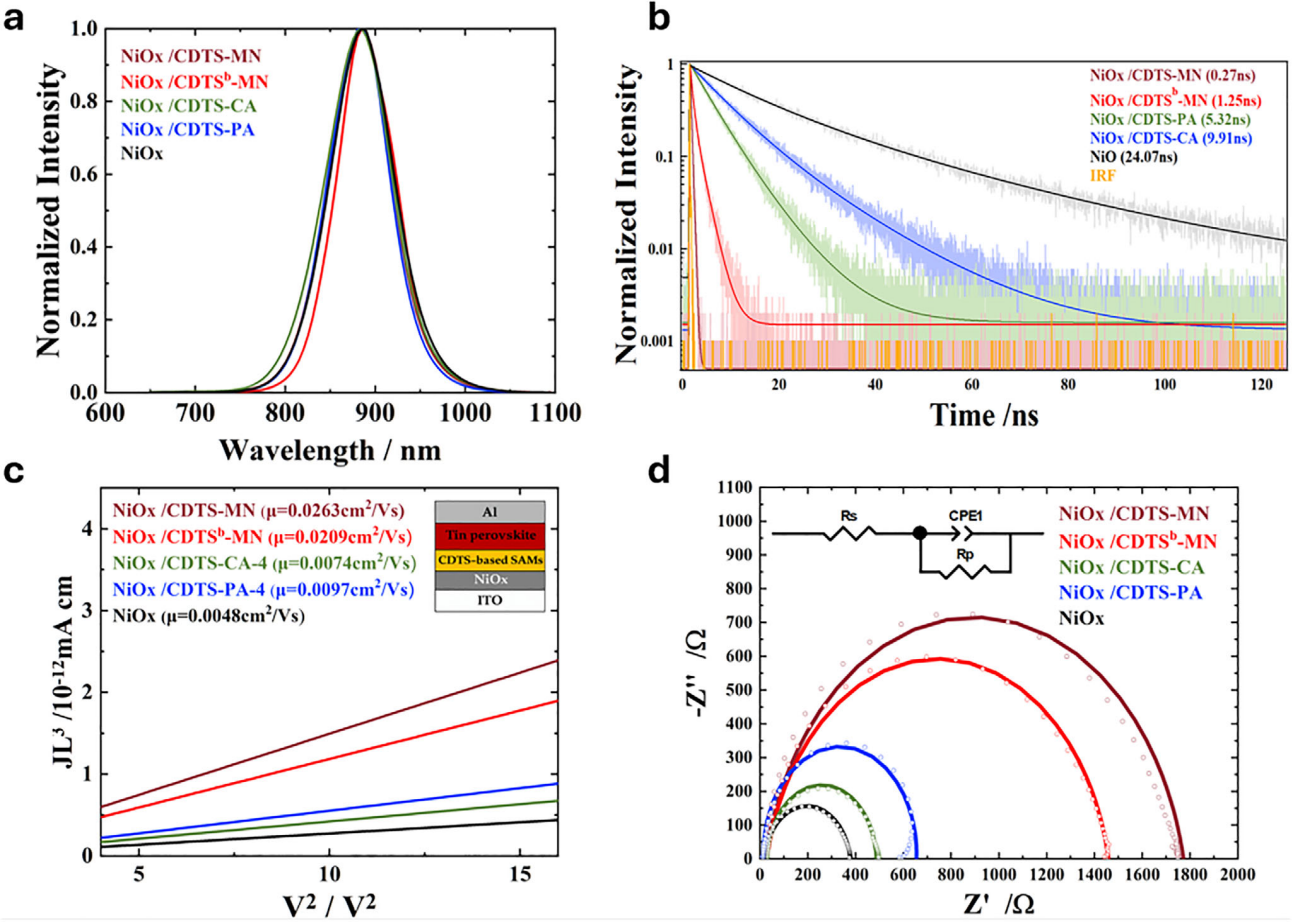
a) Steady–state PL spectra of perovskites on SAM‐modified NiOx. b) PL decay curves measured by TCSPC. c) Hole mobility extracted via SCLC from ITO/NiOx/SAM/perovskite/Al devices. d) EIS Nyquist plots of corresponding devices.

Charge transfer plays a vital role in a solar cell's operation. We replaced the PEDOT: PSS with NiOx/CDTS‐series SAMs, so the HTL should have greater hole mobility, preferable charge extraction, and reduce charge recombination. Regarding the optical characteristics, **Figure** **4**a displays the PL spectra for the four films, and their PL peak locations are comparable. To assess hole extraction in tin perovskite films incorporating CDTS‐series SAMs, we performed time‐correlated single‐photon counting (TCSPC) measurements. As depicted in Figure 4b and the results summarized in Table S3 (Supporting Information), the decay profiles indicate that **NiOx/CDTS‐MN** achieves the most rapid hole extraction, demonstrating its outstanding efficiency in this process. Initially, electron transport from the bulk to C_60_ dominates at shorter times, while interfacial recombination becomes the primary pathway in a later stage. While fast hole extraction is essential, hole mobility is another critical parameter to be studied. To quantify it, we utilized space‐charge limited current (SCLC) measurements.

In solar cells, the highest permissible current density for steady‐state electron transport is defined by space‐charge‐limited electron flow. Hole mobility was extracted from the trap‐free region using the Mott–Gurney equation, where the current is governed by space‐charge effects in the absence of trapping.^[^ ^60^ ^]^ As illustrated in Figure 4c, **NiOx/CDTS‐MN** exhibits the greatest hole mobility among all CDTS‐series SAMs in cooperation with NiOx, underscoring its superior charge transport capability compared to other CDTS‐shaped SAMs. To further analyze charge recombination characteristics, we conducted electrochemical impedance spectroscopy (EIS) measurements under dark conditions, applying open‐circuit voltage (*V*oc) as a bias. The impedance response, depicted as a single semicircle in Figure 4d, represents the resistance to interfacial charge recombination. An elevated charge recombination resistance signifies a substantial suppression of this charge recombination process. This reduction in recombination losses directly correlates with improved charge carrier lifetime and, consequently, manifests as a marked enhancement in overall device performance metrics. As shown in Figure 4f, the resistance follows the trend: **NiOx/CDTS‐MN** > **NiOx/CDTS^b^‐MN** > **NiOx/CDTS‐PA** > **NiOx/CDTS‐CA** > **NiOx‐based**, which is consistent with the observed *V*oc values.

### 2.5 Device Performance and Stability

**Figure 5:**
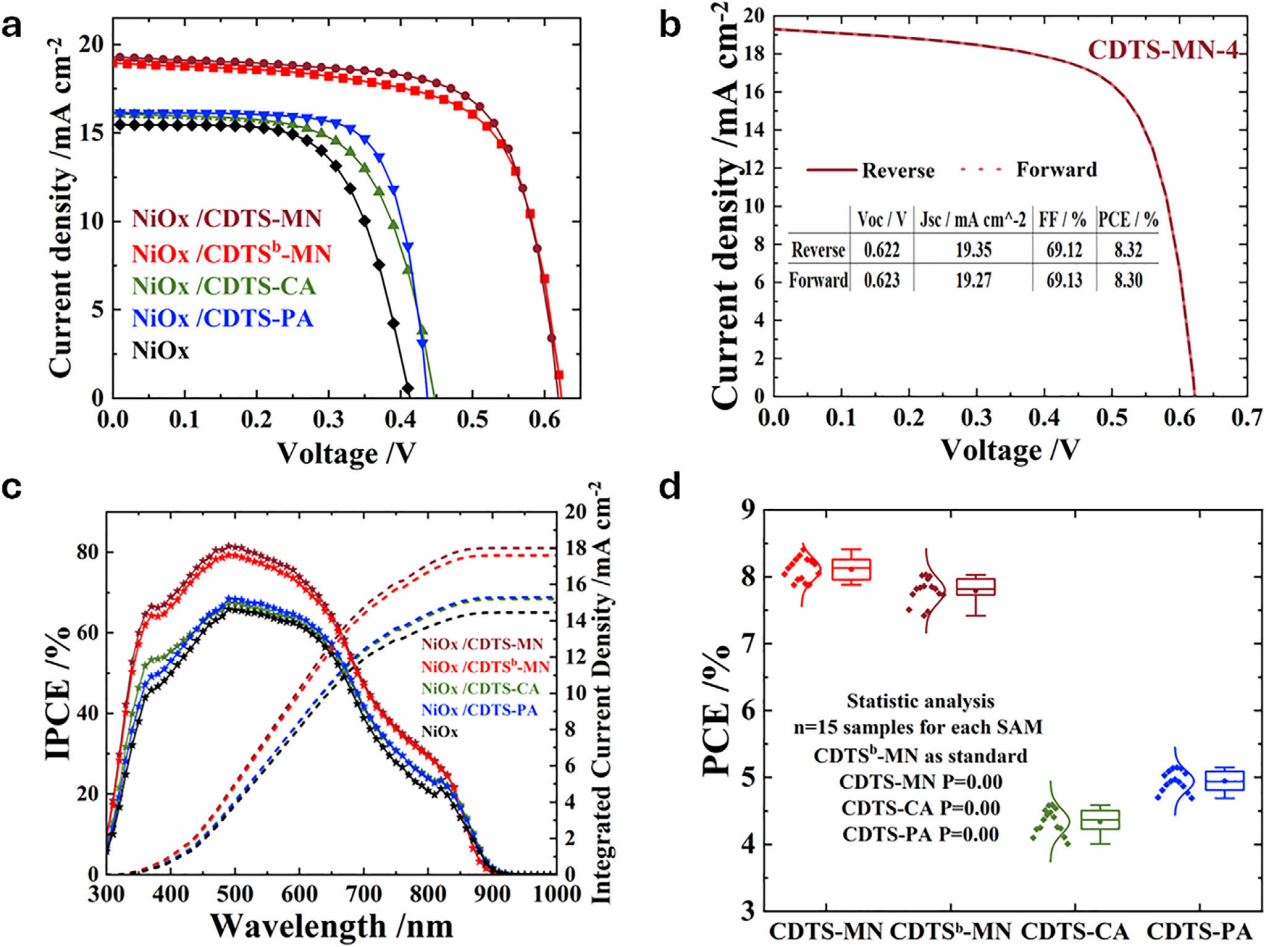
a) *J–V* characteristics of devices incorporating various CDTS‐based SAMs. b) Hysteresis behavior of the **CDTS‐MN** device. c) IPCE spectra and integrated J_SC_ values. d) Device‐to‐device PCE variation (n = 15) for each SAM.

We fabricated tin‐based perovskite solar cells featuring a multilayered architecture comprising ITO/NiOx/CDTS‐derived SAM/Sn‐based perovskite/C_60_/BCP/Ag. The *I‐V* characteristics in **Figure** **5**a show the performance of devices that incorporate NiOx/SAMs, while Table S4 (Supporting Information) provides relevant photovoltaic parameters. To compare the results with different CDTS‐series HTMs synthesized in this investigation, NiOx‐only was utilized as a control unit of this series.

The photovoltaic performance of the devices incorporating various CDTS‐based SAMs follows the trend: **NiOx/CDTS‐MN** (8.41%) > **NiOx/CDTS^b^‐MN** (8.03%) > **NiOx/CDTS‐PA** (5.15%) > **NiOx/CDTS‐CA** (4.59%) > **NiOx‐**only control (4.08%). Among them, the **NiOx/CDTS‐MN** device demonstrates the highest PCE, with a notable advantage exhibiting almost no hysteresis during the forward and reverse scan cycles, as shown in Figure 5b. Figure S40 (Supporting Information) compares device performance of CDTS‐based SAMs with and without the presence of NiOx. This negligible hysteresis strongly suggests improved interfacial contact and efficient charge transport within the device. To further substantiate these findings, the incident photon‐to‐current conversion efficiency (IPCE) spectra and the corresponding integrated short‐circuit current densities (*J*_SC_) are displayed in Figure 5c. The spectral response clearly confirms the prominent performance of the **NiOx/CDTS‐MN**‐based device, with a high capacity for photon harvesting, especially in the wavelength region of 350–700 nm (Figure S41, Supporting Information). This high IPCE correlates closely with the elevated *J*_SC_ and confirms the improved light absorption and carrier collection enabled by the **NiOx/CDTS‐MN** interface.

In addition to enhanced performance, the statistical reproducibility of the device efficiency was evaluated through fabrication and testing of 15 individual solar cells for each type of **CDTS‐**based SAM along with NiOx. The resulting PCE, *V*oc, *J*sc, and FF distribution with the raw data summarized in Tables S5–S8 (Supporting Information), are presented as box plots in Figure 5d and Figure S42 (Supporting Information), confirms that the performance of the **NiOx/CDTS‐MN** configuration is not only outstanding but also consistent, which is crucial for practical deployment of TPSCs.

**Figure 6:**
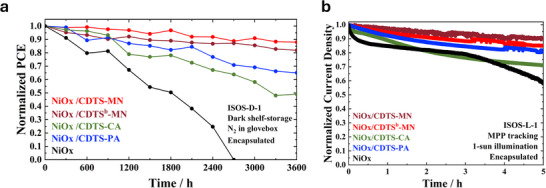
a) Shelf‐life stability (ISOS‐D‐1). b) MPPT performance under continuous illumination (ISOS‐L‐1).

Stability is critical for solar cell applications, and two types of stability assessments were conducted in this study. The first test, evaluating long‐term efficiency retention under shelf storage conditions (ISOS‐D‐1), is depicted in **Figure** **6**a. Over 3600 h, the **NiOx/CDTS‐MN** devices showed exceptional long‐term stability, retaining almost 90% of their original efficiency. Enhanced stability can be attributed to the best film morphology, crystalline structure, hole mobility, and optoelectronic properties. The second test, where we used ISOS‐L‐1 protocol to conduct the maximum power point tracking (MPPT) conditions under one sun illumination, demonstrated that the **CDTS‐MN** device retained 90.3% of its original PCE after 5 h of illumination (Figure 6b).

## 3 Conclusion

We synthesized and characterized four molecules based on dithioalkylated‐CDT unit such as **CDTS‐MN** (**1**), **CDTS^b^‐MN** (**1b**), **CDTS‐CA** (**2**), and **CDTS‐PA** (**3**), to act as SAMs for TPSCs. DFT calculations revealed that the triphenylamine groups positioned on one side of the CDTS core promote effective charge transport to the perovskite layer. Meanwhile, dicyanovinylene, cyanoacrylic acid, and phosphonic acid groups on the opposite side provide strong anchoring to the NiOx/ITO surface, which increases the hydrophilicity and surface texture of the ITO substrate, enabling uniform SAM formation. The inclusion of solubilizing thioalkyl groups to the CDT core is anticipated to improve the solubility, promote intramolecular interactions, reduce and regulate the packing of SAMs on the NiOx/ITO substrates. Single crystal structures of **CDTS‐MN** and **CDTS^b^‐MN** revealed S—S intermolecular interactions and improved π‐conjugation, aiding in the formation of dense SAMs that promote efficient charge transport. In our study, using only NiOx as a HTM in TPSC yielded a low PCE of 4.0%. However, integrating the new organic SAMs (**1**‐**3**) with NiOx significantly improved their device efficiencies, achieved an excellent PCE of 8.41% for the NiOx/**CDTS‐MN** device. In contrast, the devices made of NiOx/**CDTS^b^‐MN**, NiOx/**CDTS‐CA** and NiOx/**CDTS‐PA** achieved PCEs of 8.04%, 4.54%, 5.15% respectively. Further, extensive characterizations including SEM, AFM, KPFM, XPS, TCSPC, SCLC and EIS were performed to analyze the optoelectronic properties of the NiOx/SAMs based perovskite thin films. This research offers a promising approach to improving performance and stability in TPSCs by utilizing innovative CDTS‐based SAMs combined with NiOx HTMs, which is expected to further advance TPSC technology.

## 4 Experimental Section

### Materials

All reagents were used as received without further purification unless otherwise specified. The following chemicals were employed: the commercial ITO glass (1.9 × 1.9 cm^2^), nickel oxide nanoparticles (NiOx, Lumtech), formamidinium iodide (FAI, Greatcell Solar), tin(II) iodide (SnI_2_, 99.999%, Alfa Aesar), tin(II) fluoride (SnF_2_, 99%, Aldrich), dimethyl sulfoxide (DMSO, Aldrich), chlorobenzene (Aldrich), isopropanol (IPA, Aldrich), hexafluoro‐2‐propanol (HFP, TCI), and ethylenediammonium diiodide (EDAI_2_, Greatcell Solar). EDAI2 was synthesized by reacting an excess of hydroiodic acid (HI, 57%, Alfa Aesar) with 1,2‐diaminoethane (99%, Alfa Aesar) in an ice bath. The resulting white solid was thoroughly washed with diethyl ether and dried in a vacuum oven at 50 °C for 24 h. SAM solutions were made with dry ethanol that was obtained through distillation.

### Synthesis of CDTS‐Based SAMs

**Scheme 1:**
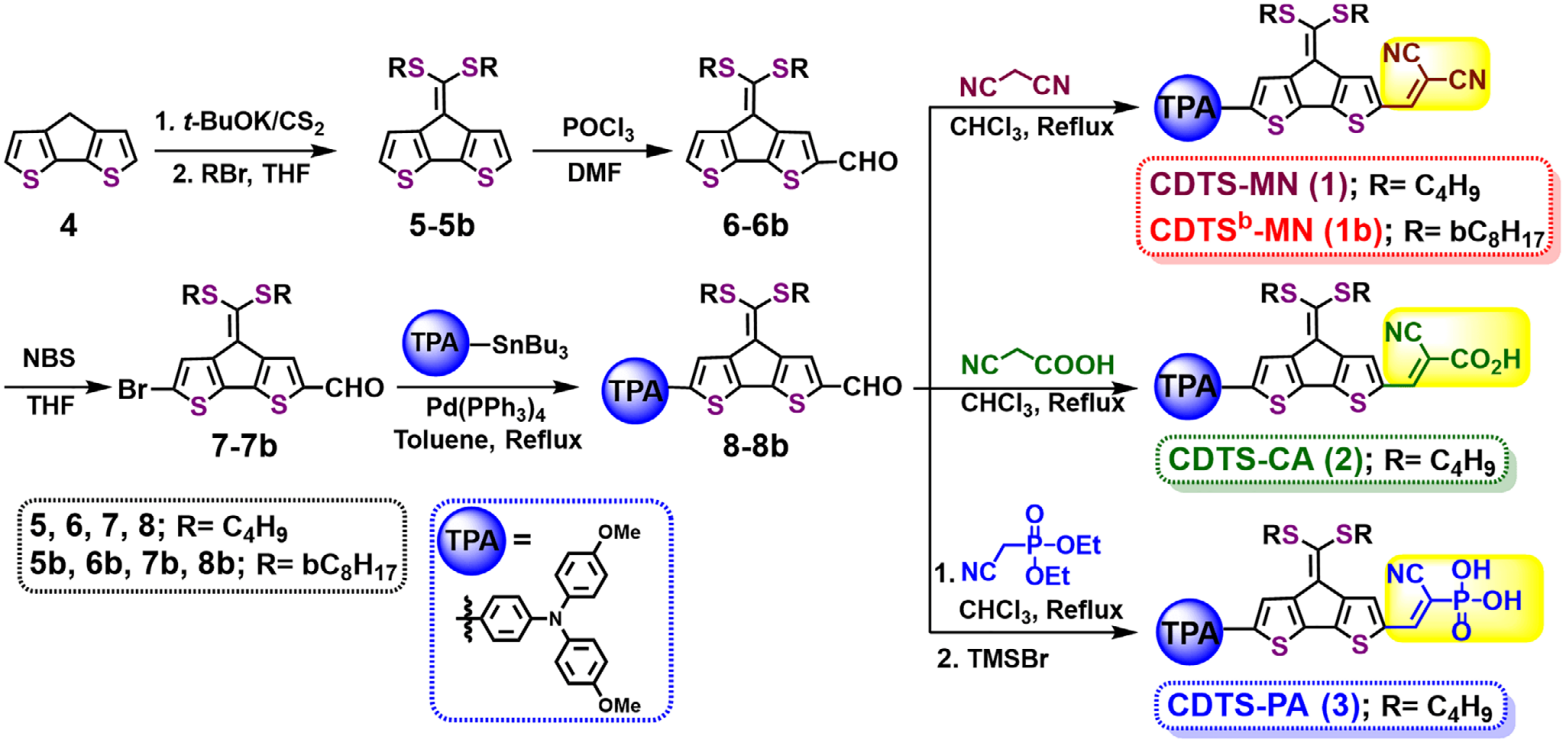
Synthesis of CDTS‐based SAMs as HTMs for TPSC application.

The synthetic pathways for SAMs molecules 1‐3 are illustrated in **Scheme** **1**. The precursor compound CDT (4) was synthesized using established methods from the literature.50, 53 Initially, compound 4 was deprotonated with t‐BuOK and subsequently treated with CS2 to generate the ketene dithiolate anion in situ.50 This anion was then alkylated using alkyl bromides (br‐C8H17 and C4H9Br), resulting in the formation of the corresponding dialkylated thioacetals, 5 and 5b, with excellent yields ranging from 84% to 95%. Following this, the dialkylated thioacetals (5‐5b) underwent a Vilsmeier‐Haack reaction, resulting in the formation of mono‐formylated moieties 6 and 6b with satisfactory yields. Further, intermediates 6 and 6b were reacted with n‐bromosuccinimide (NBS), leading to the production of brominated derivatives 7 and 7b, also achieved in high yields (85%–91%). The key aldehydes 8 and 8b were obtained through Stille coupling of bromo‐intermediates 7 and 7b with the 4‐methoxy‐N‐(4‐methoxyphenyl)‐N‐(4‐(tributylstannyl)phenyl)aniline in satisfactory yields (70%–80%). Furthermore, Knoevenagel condensation of aldehydes 8 and 8b with propanedinitrile in the presence of pyridine in chloroform produced target molecules CDTS‐MN (1) and CDTSb‐MN (1b) in good yields (≈80%). Similarly, the Knoevenagel condensation of aldehydic intermediate 8 with a mixture of cyanoacetic acid and piperidine in refluxing chloroform yielded the molecule CDTS‐CA (2) in good yield (≈70%). Finally, the target molecule CDTS‐PA (3) was synthesized by reacting the aldehydic intermediate 8 with diethyl (cyanomethyl) phosphonate, followed by hydrolysis reaction in satisfactory yield (≈65%). Molecules 1‐3 demonstrate excellent solubility in organic solvents such as chlorobenzene, chloroform, and tetrahydrofuran. Additionally, the structural analysis of all target compounds was conducted using ^1^H and ^13^C NMR spectroscopy, along with mass spectrometry.

### Preparation of Hole Transport Layer and Perovskite Films

For the preparation of hole transport layer precursors, NiOx nanoparticles were dispersed in deionized water to achieve a concentration of 0.2 mM. NiOx nanoparticle solution was spin‐coated onto the cleaned ITO substrates at 2000 rpm for 30 s and annealed at 150 °C for 10 min before the SAM dipping. After removal from the solution, the substrates were annealed at 100 °C for 10 min to remove residual solvent. For the preparation of tin perovskite precursors, 0.8 mM SnI_2_, 0.16 mM SnF_2_, and 0.008 mM EDAI_2_ were dissolved in 1 mL dimethyl sulfoxide (DMSO) and stirred overnight in a nitrogen glovebox. The antisolvent solution was prepared by dissolving 20 mg FAI in a IPA/HFP/CB cosolvent (5:5:2, v/v/v, 1.2 mL). For film deposition, 40 µL of the perovskite precursor was spin‐coated onto the SAM‐modified NiOx/ITO substrates at 6000 rpm for 1 min. Subsequently, 100 µL of the FAI antisolvent solution was loaded onto the SnI2‐coated substrates and spin‐coated at 5000 rpm for 12 s. The resulting films were immediately annealed at 70 °C for 10 min to complete crystallization.

### Device Fabrication

The commercial ITO glass substrates (1.9 × 1.9 cm^2^) were sequentially cleaned by sonication in isopropanol, acetone, and deionized water for 30 min each, followed by drying under a nitrogen stream and treatment with ultraviolet‐ozone for 30 min. The SAM solution was deposited onto the ITO substrates by dipping as described above. After the substrates had cooled to room temperature, the perovskite films were subsequently deposited onto the SAM‐modified layers using the same procedure as detailed previously. The substrates were transferred to a vacuum chamber for sequential deposition of the remaining layers. A 30 nm thick C_60_ layer was thermally evaporated as the electron transport layer, followed by a 5 nm hole‐blocking layer of BCP and a 100 nm top metal layer of Ag. All depositions were carried out under a base pressure ≈5 × 10^−6^ Torr.

### General SAM Characterizations

1H and 13C NMR spectra were recorded using a Bruker 500 instrument, with reference to solvent signals. Thermo gravimetric analysis (TGA) was performed on a Perkin Elmer TGA‐7 thermal analysis system using dry nitrogen as a carrier gas at a flow rate of 40 mL min^−1^. UV–vis absorption and fluorescence spectra were obtained in the indicated solvents at room temperature using JASCO V‐530 and Hitachi F‐4500 spectrometers, respectively. Differential pulse voltammetry (DPV) experiments were performed with a conventional three electrode configuration (a platinum disk working electrode, an auxiliary platinum wire electrode, and a non‐aqueous Ag reference electrode, with a supporting electrolyte of 0.1 M tetrabutylammonium hexafluorophosphate (dry TBAPF6) in the specified dry solvent, using a CHI621C Electrochemical Analyzer (CH Instruments).

### Characterizations of Films and Devices

*J–V* curves were obtained at a scan rate of 0.07 V s^−1^ using a SAN‐EI solar simulator (model XES‐40S1) under standard one sun illumination (AM 1.5G spectrum, 100 mW cm^2^), with calibration against a standard silicon reference cell (Oriel, model PN 91 150 V, VLSI standard). The devices' active area was 0.0225 cm^2^, as specified by a metal mask. Following calibration with a standard silicon photodiode (Hamamatsu model S1337‐1012BQ), incident photon‐to‐current efficiency (IPCE) spectra were produced. X‐ray diffraction (XRD) patterns were measured using a Bruker D8‐Advance diffractometer with Cu Kα radiation. The samples' morphology and structure were studied utilizing a field‐emission scanning electron microscopy (SEM, Hitachi model SU8010) and an atomic force microscope and Kelvin probe force microscopy (AFM, KPFM, SII Nanotechnology Inc. model VT SPM). UV–vis/NIR absorption spectra have been collected using a Jasco V‐570 spectrophotometer with a spherical attachment for increased sensitivity. PL measurements were performed using 450 nm excitation with emission spectra recorded from 700 to 1100 nm.

### Carrier Dynamic Measurements

For PL decay studies, a time‐correlated single‐photon counting (TCSPC) system (Fluotime 200, PicoQuant) was used with excitation at 635 nm, the PL decays were monitoring at 840 nm. The laser operated at a repetition rate of 25 MHz and a pulse energy of 4 µJ cm^−2^. Faster decay components (τ_1_) were attributed to interfacial charge recombination, while slower components (τ_2_ or τ_3_) reflected bulk recombination processes. Electrochemical impedance spectra (EIS) were measured to probe interfacial charge carrier recombination in the devices.^[^ ^54^ ^]^ Measurements were done in the dark with the bias at the open‐circuit voltage (VOC). Nyquist plots showed a single semicircle, indicating carrier recombination resistance, with the semicircle size reflecting the magnitude of this resistance.^[^ ^55^ ^]^ The data were fitted using an equivalent circuit with a series resistance (Rs) and a parallel RC element representing the carrier dynamics. Hole‐only devices with an ITO/HTL/perovskite/Al structure were prepared for space charge limited current (SCLC) measurements. The hole transport layer (HTL) and perovskite were deposited using the same process as the photovoltaic devices. The Al electrode was thermally evaporated at a base pressure of ≈5 × 10^−6^ Torr. Dark *J–V* scans were recorded from 0 to 5 V using a Keithley 2400 source meter. The hole mobility was calculated using the Mott–Gurney law.

### Statistical Analysis

Statistical methods were used to analyze various photovoltaic parameters of the TPSCs based on all SAMs by Statistical Product and Service Solutions (SPSS) software. 15 devices were randomly selected for statistics; their raw data were listed in Tables S5–S8 (supporting Information). The corresponding boxplots were shown in Figure 5d and Figure S42 (Supporting Information). Their minimum/maximum/average PCE standard deviation and P value (**CDTS^b^‐MN** as a standard sample) were summarized in the following:

*CDTS^b^‐MN*: *V*_OC_: 0.535–0.625 V, 0.581±0.027 V. *J*_SC_: 18.96–20.18 mA cm^2^, 19.65±0.35 mA cm^2^. FF: 65.8%–71.6%, 68.4±2.0%. PCE: 7.4%–8%, 7.8±0.2%.

*CDTS‐MN*: *V*_OC_: 0.558–0.623 V, 0.589±0.016 V; P = 0.32. *J*_SC_: 19.27–20.14 mA cm^2^, 19.81±0.30 mA cm^2^; P = 0.21. FF: 66%–74.2%, 69.6±2.4%; P = 0.14. PCE: 7.9%–8.4%, 8.1±0.2%; P = 0.00.

*CDTS‐PA*: *V*_OC_: 0.424–0.492 V, 0.462±0.027 V; P = 0.00. *J*_SC_: 13.66–17.96 mA cm^2^, 15.58±1.36 mA cm^2^; P = 0.00. FF: 66.1%–72.2%, 69.0±2.0%; P = 0.41. PCE: 4.7%–5.2%, 4.9±0.2%; P = 0.00.

*CDTS‐CA*: *V*_OC_: 0.412–0.551 V, 0.475±0.040 V; P = 0.00. *J*_SC_: 12.41–16.57 mA cm^2^, 14.63±1.31 mA cm^2^; P = 0.00. FF: 60.3%–65.7%, 62.8±1.7%; P = 0.00. PCE: 4‐4.6%, 4.3±0.2%; P = 0.00.

## Conflict of Interest

The authors declare no conflict of interest.

## Author Contributions

S.N.A, Y.‐S.S, C.‐H.K, and C.‐H.K contributed equally to this work. S.N.A, Y.‐S.S, M.‐C.C, and E.W.‐G.D designed and directed the study. C.‐H.K and P.‐Y.H contributed to device fabrication. S.N.A, A.V., and P.‐Y.L. contributed to synthesis of SAMs. S.N.A, Y.‐S.S and C.‐H.K contributed to organic material characterization and device characterization along with data analysis. S.‐H.H and C.‐L.L carried out and interpreted the DFT studies. X.J. supported the instrument analysis. M.‐C.C, and E.W.‐G.D supervised the whole project. S.N.A, Y.‐S.S, C.‐H.K, M.‐C.C, and E.W.‐G.D wrote the manuscript. E.W.‐G.D completed the formatting and finalizing manuscript draft.

## Supporting information



Supporting Information

## Data Availability

Research data are not shared.
