# Supplementary material for: Harnessing Functionalized Thioalkylated‐Cyclopentadithiophene Monolayers on NiOx for Two‐Step Fabricated Tin Perovskite Solar Cells
Source: Small Methods. 2025 Sep 3;9(11):e01309. doi: 10.1002/smtd.202501309 (PMC12641338; doi:10.1002/smtd.202501309)
Supplement: Supplementary file 1 — Supporting Information [file SMTD-9-e01309-s001.docx]

Supporting Information

**Harnessing Functionalized Thioalkylated-Cyclopentadithiophene Monolayers on NiOx for Two-step Fabricated Tin Perovskite Solar Cells**

*Shakil N. Afraj,^a ‡^* *Yun-Sheng Shih,^b,c‡^* *Che-Hsin Kuo, ^b,c‡^ Chun-Hsiao Kuan, ^b,c‡^ Pei-Yu Huang, ^b,c^ Pei-Yun Lee,^a^ Arulmozhi Velusamy, ^a^ Shao-Huan Hong,^d^ Cheng-Liang Liu,^d^ Ming-Chou Chen,^a,^* Eric Wei-Guang Diau,^b,c^**

*^a^*Department of Chemistry, National Central University, No. 300 Zhongda Rd., Taoyuan 320317, Taiwan. Email: [mcchen@ncu.edu.tw](mailto:mcchen@ncu.edu.tw)

*^b^*Department of Applied Chemistry and Institute of Molecular Science, National Yang Ming Chiao Tung University, 1001 Ta-Hseuh Rd., Hsinchu 300093, Taiwan.

E-mail: [diau@nycu.edu.tw](mailto:diau@nycu.edu.tw)

*^c^*Center for Emergent Functional Matter Science, National Yang Ming Chiao Tung University 1001 Ta-Hseuh Rd., Hsinchu 300093, Taiwan.

*^d^*Department of Materials Science and Engineering, National Taiwan University, No. 1, Sec. 4, Roosevelt Rd., Taipei 106319, Taiwan.

Shakil N. Afraj, Yun-Sheng Shih, Che-Hsin Kuo, and Chun-Hsiao Kuan contributed equally to this work.

KEYWORDS: Self-assembled monolayers, Thioalkylated-cyclopentadithiophene, high efficiency, tin perovskite, high-stability.

**Scheme S1.** Synthesis of compounds **5** and **5b**.

**General procedure for the synthesis of compounds 5** and **5b:** Under anhydrous condition solution of 4H-cyclopenta[2,1-b:3,4-b']dithiophene (**4**) (1.5 mmol) in anhydrous THF (15 ml) was added to potassium tert-butoxide (3.6 mmol) containing flask using dropping funnel at 0°C and stirred the reaction for 1 hour. Carbon disulfide (1.9 mmol) was added via syringe to the reaction mixture and stirred for 10 min. Next, alkyl bromides (3.2 mmol) was added a via syringe over 5 min and the reaction mixture was stirred overnight under dark. After completion of reaction, it was quenched by aqueous ammonium chloride and THF was removed by rotary evaporator and extracted with water and ether, organic layer was dried over sodium sulphate. Organic layer was concentrated and crude product was purified by column chromatography using hexane as eluent to give desired products **5–5b**.

**Compound 5:** The title product was purified by column chromatography (eluent: n-hexane), yielding the product **5** as a deep orange liquid (0.33 g, 83%). ^1^H NMR (500 MHz, CDCl_3_) *δ* 7.88 (d, *J* = 4.9 Hz, 1H), 7.04 (d, *J* = 4.9 Hz, 1H), 2.98 (t, *J* = 7.3 Hz, 2H).

**Compound 5b:** The title product was purified by column chromatography (eluent: hexane) to afford the product **5b** as a deep red liquid (1 g, 77% yield). ^1^H NMR (500 MHz, CDCl_3_) *δ* 7.87 (d, *J* = 4.3 Hz, 1H), 7.04 (d, *J* = 4.4 Hz, 1H), 3.00 (d, *J* = 6.0 Hz, 2H).

**Scheme S2.** Synthesis of compounds **6** and **6b**.

**Compound 6:** Under anhydrous condition, POCl_3_ (0.28 mL, 2.82 mmol) was added dropwise at 0°C into a solution of DCE/DMF (0.8 mL, 9.4 mmol). The mixture was stirred for 1 hour, then compound **5** (0.3 g, 0.94 mmol) was added. After warming to room temperature, the reaction was allowed to proceed overnight. Completion of the reaction was confirmed by TLC. The reaction was quenched with deionized water, followed by extraction with ethyl acetate and deionized water. The organic layer was collected, dried over anhydrous sodium sulfate, and concentrated under reduced pressure. The crude product was purified by column chromatography (eluent: n-hexane), affording the product **6** as an orange liquid (0.4 g, 70%). ^1^H NMR (300 MHz, CDCl_3_) *δ* 9.84 (s, 1H), 8.50 (s, 1H), 7.93 (d, *J* = 5.1 Hz, 1H), 7.29 (d, *J* = 5.0 Hz, 1H), 3.03 (t, *J* = 7.5, 1.9 Hz, 4H).

**Compound 6b:** Under anhydrous conditions, POCl_3_ (0.3 mL, 3.35 mmol) was slowly added dropwise to a mixture of DCE/DMF (0.9 mL, 33.5 mmol) at 0°C and the mixture was stirred for 1 hour. Compound **5b** (0.2 g, 1.2 mmol) was then added, and the reaction was allowed to warm to room temperature and stirred overnight. The completion of the reaction was confirmed by TLC. The reaction was quenched with deionized water, and the mixture was extracted with ethyl acetate and deionized water. The organic layer was collected, dried over anhydrous sodium sulfate, and concentrated under reduced pressure. The crude product was purified by column chromatography (eluent: hexane) to afford the product **6b** as an orange liquid (0.4 g, 70% yield). ^1^H NMR (500 MHz, CDCl_3_) δ 9.84 (s, 1H), 8.51 (s, 1H), 7.92 (d, *J* = 5.0 Hz, 1H), 7.30 (d, *J* = 4.9 Hz, 1H), 3.04 (d, *J* = 5.8 Hz, 4H).

**Scheme S3.** Synthesis of compounds **7** and **7b**.

**Compound 7:** Compound **6** (0.3 g, 0.91 mmol) and THF (10 mL) were placed in a flask. At 0°C, a solution of NBS (0.19 g, 1.09 mmol) in THF (10 mL) was added dropwise slowly to the flask, and the reaction was allowed to proceed overnight. The reaction completion was confirmed by TLC. The reaction was quenched with deionized water, followed by extraction with ethyl acetate. The organic layer was collected, dried over anhydrous sodium sulfate, and concentrated under reduced pressure to remove the solvent. The crude product **7** was purified by column chromatography (eluent: n-hexane), affording the product as an orange liquid (0.4 g, 93%). ^1^H NMR (500 MHz, CDCl_3_) δ 9.88 (s, 1H), 8.51 (s, 1H), 7.99 (s, 1H), 3.05 (m, 4H).

**Compound 7b**: Compound **6b** (0.2 g, 0.61 mmol) was placed in a flask with THF (10 ml). A solution of NBS (0.13 g, 0.73 mol) in THF (10 ml) was slowly added dropwise at 0°C. The reaction mixture was stirred overnight. The completion of the reaction was confirmed by TLC. The reaction was quenched with deionized water and extracted with ethyl acetate. The organic layer was collected, dried over anhydrous sodium sulfate, and concentrated under reduced pressure. The crude product **7b** was purified by column chromatography (eluent: hexane) to afford the product as an orange liquid (0.4 g, 71% yield). ^1^H NMR (500 MHz, CDCl_3_) δ 9.85 (s, 1H), 8.48 (s, 1H), 7.97 (s, 1H), 3.05 (m, 4H).

**Scheme S4.** Synthesis of key intermediates **8** and **8b**.

**Compound 8**: Under anhydrous condition, compound **7** (0.2 g, 0.42 mmol), 4-methoxy-N-(4-methoxyphenyl)-N-(4-(tributylstannyl)phenyl)aniline (0.35 g, 0.59 mmol), and Pd(PPh_3_)_4_ (0.06 g, 0.06 mmol) were dissolved in anhydrous toluene (20 mL). The reaction mixture was heated to reflux for 48 hours, then cooled to room temperature. After filtration through celite under vacuum, the solvent was removed under reduced pressure. The crude product was purified by column chromatography (eluent: ethyl acetate:hexane = 20:80), affording the product as a black solid (0.2 g, 69%). ^1^H NMR (500 MHz, DMSO-d_6_) δ 9.89 (s, 1H), 8.61 (s, 1H), 8.07 (s, 1H), 7.52 (s, 2H), 7.09 (m, 4H), 6.97 (m, 4H), 6.83 (m, 2H), 3.78 (s, 6H), 3.11 (m, 4H). ^13^C NMR (125 MHz, CDCl_3_) δ 182.5, 156.14, 148.69, 148.53, 148.23, 147.51, 140.29, 134.40, 134.0, 126.81, 126.29, 120.03, 119.78, 114.73, 55.44, 34.91, 32.27, 21.99, 21.95, 13.61.

**Compound 8b**: Under anhydrous condition, compound **7b** (0.2 g, 0.34 mmole), 4-methoxy-N-(4-methoxyphenyl)-N-(4-(tributylstannyl)phenyl)aniline (0.28 g, 0.48 mmole), and Pd(PPh_3_)_4_ (0.06 g, 0.06 mmol) were dissolved in anhydrous toluene (20 mL). The mixture was heated under reflux for 48 hours. After the reaction was complete, the mixture was cooled to room temperature and filtered through Celite under vacuum. The solvent was removed under reduced pressure, and the crude product was purified by column chromatography (eluent: ethyl acetate = 20:80) to afford the product as a black liquid (0.3 g, 88% yield). ^1^H NMR (500 MHz, DMSO-d_6_) δ 9.87 (s, 1H), 8.57 (s, 1H), 8.05 (s, 1H), 7.46 (d, J = 8.2 Hz, 2H), 7.08 (d, *J* = 8.6 Hz, 4H), 6.95 (d, *J* = 8.5 Hz, 4H), 6.79 (d, *J* = 8.8 Hz, 2H), 3.76 (s, 6H), 3.12 (s, 4H); ^13^C NMR (125 MHz, CDCl_3_) δ 182.49, 156.12, 148.66, 140.31, 134.39, 126.79, 126.20, 120.07, 119.69, 114. 72, 55.44, 40.07, 39.46, 32.49, 32.41, 28.83, 28.13, 26.74, 25.73, 22.95, 17.29, 14.00, 13.55, 10.87.

**Synthesis of SAM CDTS-MN (1):** Under an anhydrous condition, compound **8** (0.1 g, 0.12 mmol), malonitrile (0.82 g, 1.23 mmol), and pyridine (1.0 mL) were dissolved in anhydrous chloroform (20 mL). The reaction mixture was refluxed for 24 hours, then cooled to room temperature. The reaction was quenched with deionized water, followed by extraction with ethyl acetate and deionized water. The organic layer was collected, dried over anhydrous sodium sulfate, and concentrated under reduced pressure to remove the solvent. The crude product was purified by column chromatography (eluent: ethyl acetate:n-hexane = 40:60), affording the product as a black solid (0.8 g, 76%). ^1^H NMR (500 MHz, CDCl_3_) *δ* 8.45 (s, 1H), 8.03 (s, 1H), 7.71 (s, 1H), 7.43 (d, *J* = 8.1 Hz, 2H), 7.10 (d, *J* = 8.7 Hz, 4H), 6.92 (d, *J* = 8.3 Hz, 2H), 6.86 (d, *J* = 8.6 Hz, 4H), 3.81 (s, 6H), 3.04 (t, *J* = 7.1 Hz, 4H); ^13^C NMR (126 MHz, CDCl_3_) *δ* 127.06, 126.57, 119.77, 119.69,114.87, 55.53, 35.34, 35.34, 32.35, 13.62. HRMS (HR-FAB [M]+) calcd for C_42_H_39_N_3_O_2_S2: 745.1900, Found: 745.1920.

**Scheme S5.** Synthesis of target SAMs; **1, 1b, 2-3**.

**Synthesis of SAM CDTS^b^-MN (1b):** Under anhydrous condition at 0°C, compound **7b** (0.1 g, 0.12 mmol) was added dropwise into a solution of malononitrile (0.82 g, 1.23 mmol) and pyridine (1.0 mL) dissolved in anhydrous chloroform (20 mL). The reaction mixture was refluxed for 24 hours, then cooled to room temperature. The reaction was quenched with deionized water, followed by extraction with ethyl acetate and deionized water. The organic layer was collected, dried over anhydrous sodium sulfate, and concentrated under reduced pressure to remove the solvent. The crude product was purified by column chromatography (eluent: ethyl acetate:n-hexane = 40:60), affording the product as a black solid (0.8 g, 76%). ^1^H NMR (500 MHz, CDCl_3_) *δ* 8.43 (s, 1H), 8.05 (s, 1H), 7.71 (s, 1H), 7.43 (s, 2H), 7.08 (s, 4H), 6.87 (s, 6H), 3.82 (s, 6H), 3.06 (s, 4H). ^13^C NMR (126 MHz, CDCl_3_) *δ* 156.41, 154.84, 151.16, 150.26; 149.26, 140.15, 133.69, 119.79, 55.55, 40.20, 32.49, δ 28.88, 25.77, 22.97, 14.03, 10.89. HRMS (HR-FAB [M]^+^) calcd for C_50_H_55_N_3_O_2_S2 : 857.3200, Found: 857.3172.

**Synthesis of SAM CDTS-CA (2):** Under anhydrous condition, compound **8** (0.1 g, 0.11 mmol), 2-cyanoacetic acid (0.19 g, 2.27 mmol), and pyridine (1 mL) were dissolved in anhydrous chloroform (20 mL). The reaction mixture was refluxed for 24 hours, then cooled to room temperature. The reaction was quenched with methanol, followed by vacuum filtration and washing with methanol/n-hexane. The crude product was recrystallized from dichloromethane/n-hexane, affording the product as a black solid (0.1 g, 65%). ^1^H NMR (500 MHz, DMSO-d_6_) δ 8.59 (s, 1H), 8.33 (s, 1H), 8.03 (s, 1H), 7.49 (d, *J* = 7.9 Hz, 2H), 7.08 (d, *J* = 7.8 Hz, 3H), 6.95 (d, *J* = 8.7 Hz, 3H), 6.81 (d, *J* = 7.9 Hz, 2H), 3.76 (s, 6H), 3.10 (m, 4H). ^13^C NMR (75 MHz, DMSO-d_6_) *δ* 156.46, 148.71, 152.86, 146.92, 144.67, 140.04 (s), 134.34, 152.86, 133.03, 127.35, 126.55, 119.66, 115.47, 55.73, 32.29, 21.75, 13.94; HRMS (HR-FAB [M]^+^) calcd for C_42_H_40_N_2_O_4_S2 : 764.1900, Found : 763.1865.

**Synthesis of intermediate; CDTS-PE:** Under anhydrous condition, compound **8** (0.2 g, 0.48 mmol), diethyl (cyanomethyl)phosphonate (0.26 g, 1.44 mmol), and pyridine (1.0 mL) were dissolved in anhydrous chloroform (20 mL). The reaction mixture was refluxed for 24 hours, then cooled to room temperature. The reaction was quenched with deionized water, followed by extraction with dichloromethane and deionized water. The organic layer was collected, dried over anhydrous sodium sulfate, and concentrated under reduced pressure to remove the solvent. The crude product was purified by column chromatography (eluent: ethyl acetate: n-hexane = 55:45), affording the product as a black solid (0.2 g, 83%). ^1^H NMR (500 MHz, DMSO-d_6_) δ 8.70 (s, 1H), 8.24 (d, *J*_P-H_ = 19.1 Hz, 1H), 8.07 (d, *J* = 22.3 Hz, 1H), 7.50 (d, *J* = 8.0 Hz, 2H), 7.09 (d, *J* = 8.1 Hz, 4H), 6.95 (d, *J* = 8.1 Hz, 4H), 6.81 (d, *J* = 7.9 Hz, 2H), 4.23 (m, 4H), 3.76 (s, 6H), 3.10 (d, *J* = 6.5 Hz, 4H). ^13^C NMR (75 MHz, DMSO-d_6_) *δ* 165.51, 156.63, 134.22, 129.91, 129.09, 127.54, 126.78, 122.80, 121.53, 119.41, 115.53, 106.31, 70.25, 67.63, 55.75, 38.72, 32.30, 30.38, 28.84, 23.88, 22.83, 21.74, 14.33, 13.89, 11.35.

**Synthesis of SAM CDTS-PA (3):** Under anhydrous condition, compound **CDTS-PE** (0.1 g, 0.48 mmole) was dissolved in anhydrous dichloromethane (20 mL). Bromotrimethylsilane (0.26 g, 1.44 mmol) was added dropwise at 0°C. The reaction mixture was stirred at room temperature for 24 hours. The reaction was quenched by adding methanol, followed by vacuum filtration with methanol/hexane washing. Recrystallization (dichloromethane/hexane) afforded the product **3** as a black solid (0.8 g, 89% yield). ^1^H NMR (500 MHz, DMSO-d_6_) δ 8.45 (s, 1H), 7.92 (s, 2H), 7.41 (s, 2H), 7.05 (d, *J* = 6.9 Hz, 4H), 6.93 (d, *J* = 7.1 Hz, 4H), 6.78 (d, *J* = 7.2 Hz, 2H), 3.75 (s, 6H), 3.08 (s, 4H), 1.68 – 1.59 (m, 4H), 1.42 (s, 4H), 0.96 – 0.82 (m, 6H). ^13^C NMR (126 MHz, DMSO-d_6_) δ 170.79 (s), δ 156.57 (s), 147.65 (s), δ 148.94 (s), δ 147.83 (s), δ 144.60 (s), 140.03 (s), δ 134.03 (s), δ 133.40 (s), 132.66 (s), 127.44 (s), 126.70 (s), 119.58 (s), 115.53 (s), 60.22 (s), 55.76 (s), 34.99 (s), 32.29 (s), 21.75 (s), 21.21 (s), 14.55 (s), 13.91 (s). ^31^P NMR (202 MHz, DMSO-d_6_) δ 6.48 (s). HRMS (HR-FAB [M]^+^) calcd for C_41_H_41_N_2_O_5_PS2 : 800.1600, Found : 800.1630.

**
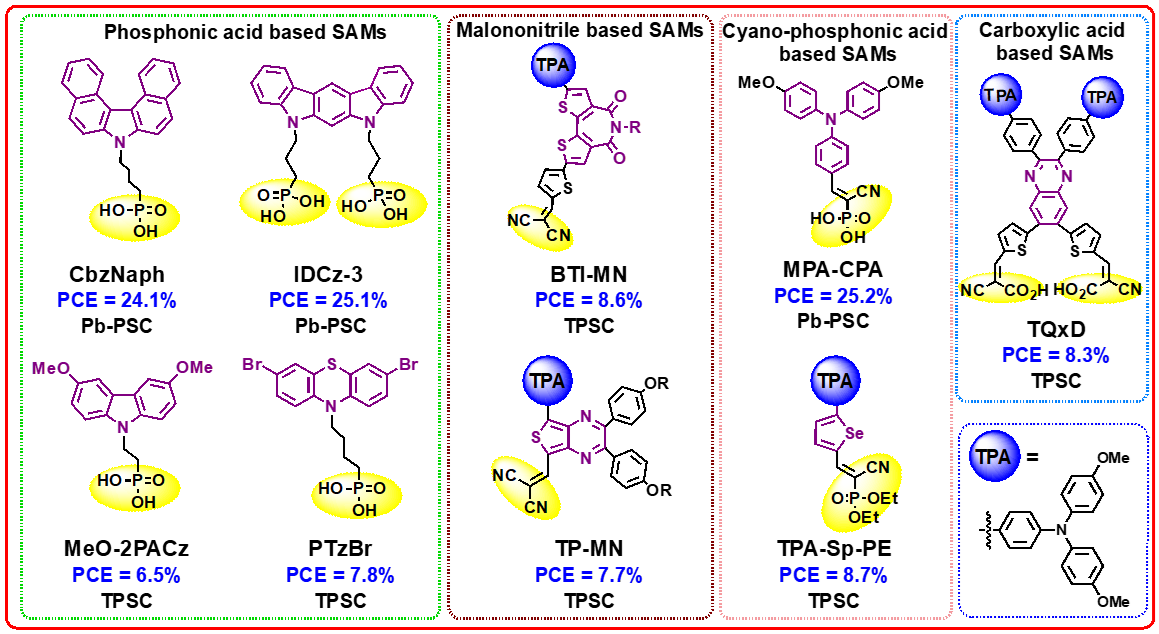
**

**Figure S1.** Molecular structures of the reported SAMs for Pb- and Sn-PSCs categorized by their anchoring groups.

***Single crystal Analysis***

To gain a better understanding of the SAM molecules structure, single crystals of **CDTS-MN** and **CDTS^b^-MN** were grown by vapor difussion method. The single crystal structures, determined through diffraction are displayed in **Figure S2**, with the corresponding crystal data provided in **Table S3** of SI. Both molecules crystallize in the triclinic system with a P-1 space group. In **Fig. S2a**, the intramolecular distances are 3.39 Å (S---N), 2.73 Å (S---H), 2.75 Å (S_alkyl_---H), and 3.05 Å (S_alkyl_---S_alkyl_), with the SAM molecule anchored through the two cyano groups. Similarly, **Fig. S2d**, shows intramolecular distances of 3.32 Å (S---N), 2.75 Å (S---H), 2.75-2.80 Å (S_alkyl_---H), and 3.04 Å (S_alkyl_---S_alkyl_). These interactions likely facilitate the formation of well-ordered SAMs on the substrate, characterized by compact and angled textures, as illustrated in **Fig. S2a,d**, where the short C=C double bond (1.36 Å) between the thiophene and CN groups is observed. Furthermore, the small interplanar angles between thiophene and the CN group 8.6° in **CDTS-MN** (**Fig. S2a**), and 13.4° in **CDTS^b^-MN** (**Fig. S2d**) indicate that **CDTS-MN** possesses a more planar molecular backbone compared to **CDTS^b^-MN**. This increased planarity is favorable for efficient π-conjugation and can potentially enhance charge transport properties. Similarly, the dihedral angles between the dithioalkyl methylene units and the central core ~8.5° for **CDTS-MN**, and 14.0° for **CDTS^b^-MN** further support this conclusion. Additionally, the larger interplanar angles observed for the phenyl rings in the TPA groups ~64.0°/63.2° for **CDTS-MN** (**Fig. S2a**) and 79.9°/54.2° for **CDTS^b^-MN** (**Fig. S2d**) suggest improved solubility. The angle between the TPA unit and central core is 24.5° and 12.0° for **CDTS-MN** and **CDTS^b^-MN**, respectively. The wide angles (~116°) formed between the two CN units in both compounds (**Fig. S2a, d**) likely help the SAM molecules remain firmly anchored to the NiOₓ substrate. Additionally, the wide angles (~120°) observed between the two S-alkyl groups (**Fig. S2a, d**) may contribute to the overall molecular stability and packing behavior. Further, side views of molecules **CDTS-MN** and **CDTS^b^-MN** illustrating interplanar angles between phenyl rings in the TPA groups with the molecular length of 22.27 Å for **CDTS-MN**, and 21.20 Å for **CDTS^b^-MN** (**Fig. S2b, e**). The proposed packing arrangement of **CDTS-MN** (anchored via two legs) and **CDTS^b^-MN** (anchored via one leg) SAM molecules on the NiOx substrate, as illustrated in **Fig. S2c** & **f**, is believed to contribute to more efficient charge transport, thereby enhancing TPSC performance.^[1-3]^


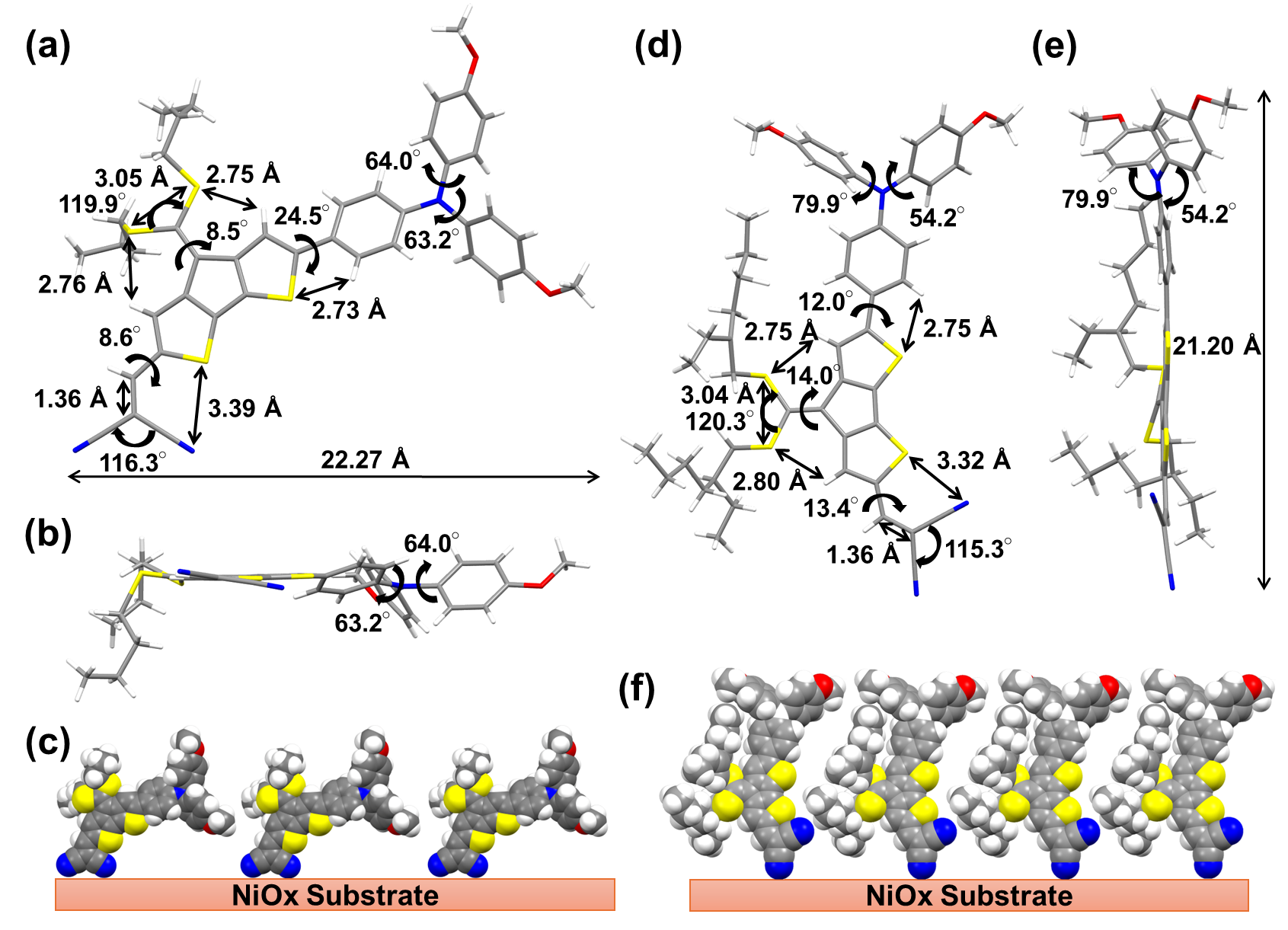


**Figure S2.** Single crystal structures of **CDTS-MN** and **CDTS^b^-MN** SAMs. (a, d) Top views of molecules **CDTS-MN** and **CDTS^b^-MN** showing intramolecular interactions and interplanar angles; (b, e) Side views of molecules **CDTS-MN** and **CDTS^b^-MN** illustrating interplanar angles; (c, f) Proposed packing patterns of the SAMs on a NiOx/ITO substrate. **a-c** corresponds to **CDTS-MN**, while **d-f** corresponds to **CDTS^b^-MN**.


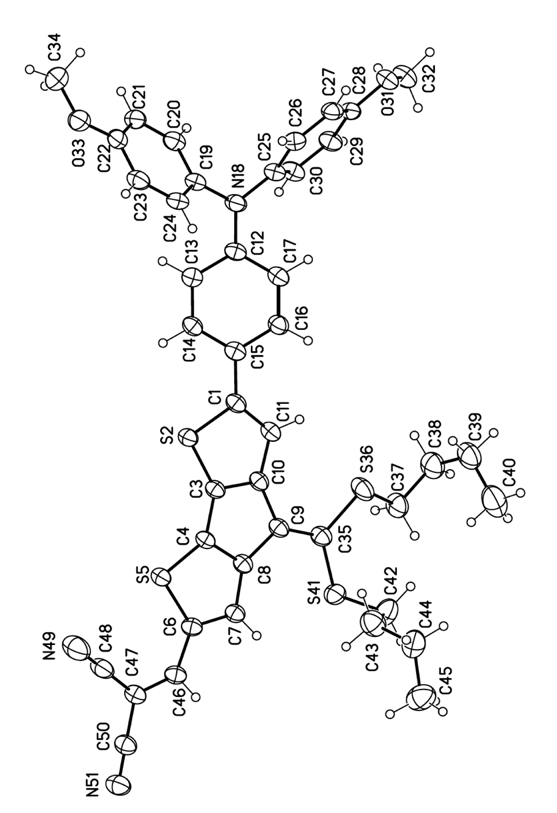


**Figure S3.** Perspective ORTEP drawing of the molecular structures of **CDTS-MN (CCDC: 2447099)**.

**
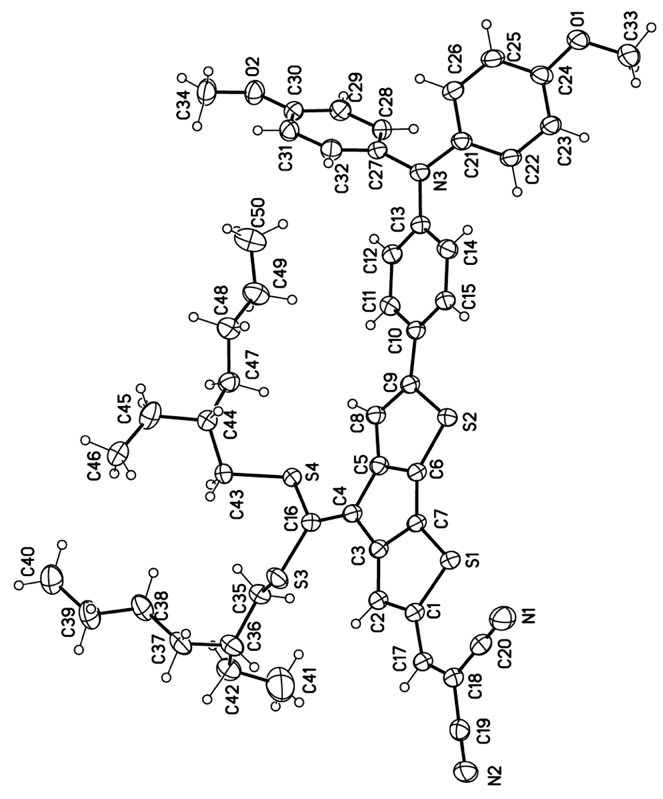
**

**Figure S4.** Perspective ORTEP drawing of the molecular structures of **CDTS^b^-MN (CCDC: 2447108)**.


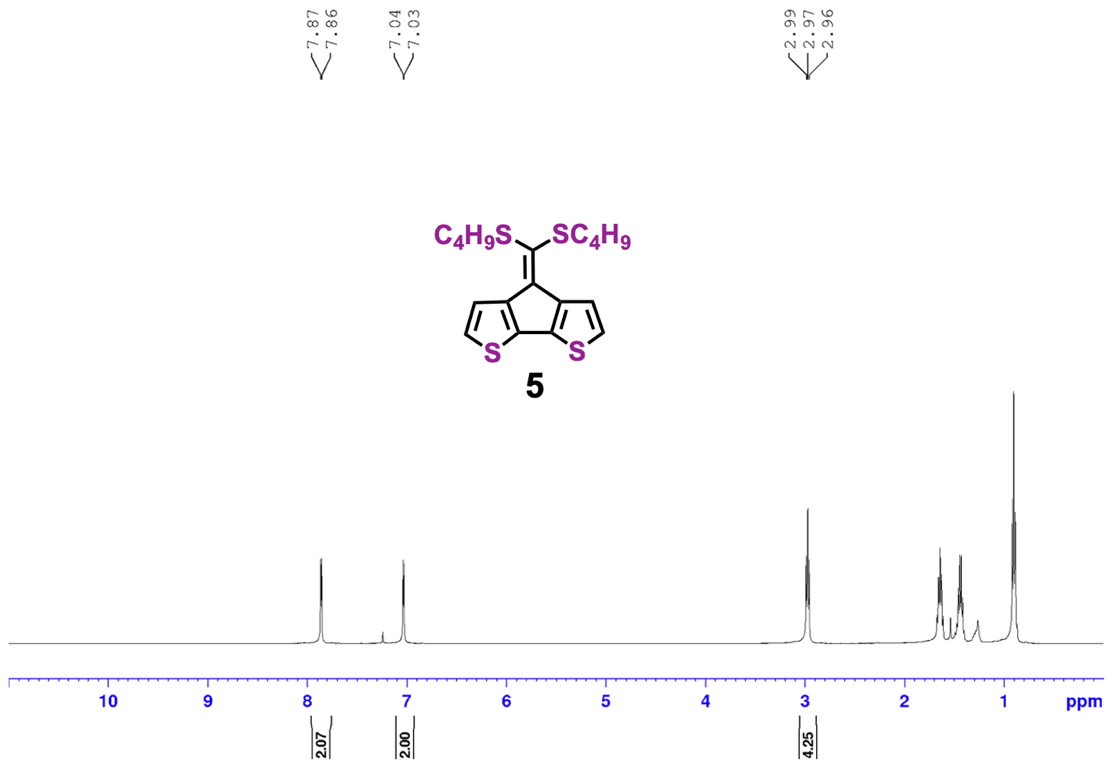


**Figure S5.** ^1^H NMR spectrum of **5** in CDCl_3_.


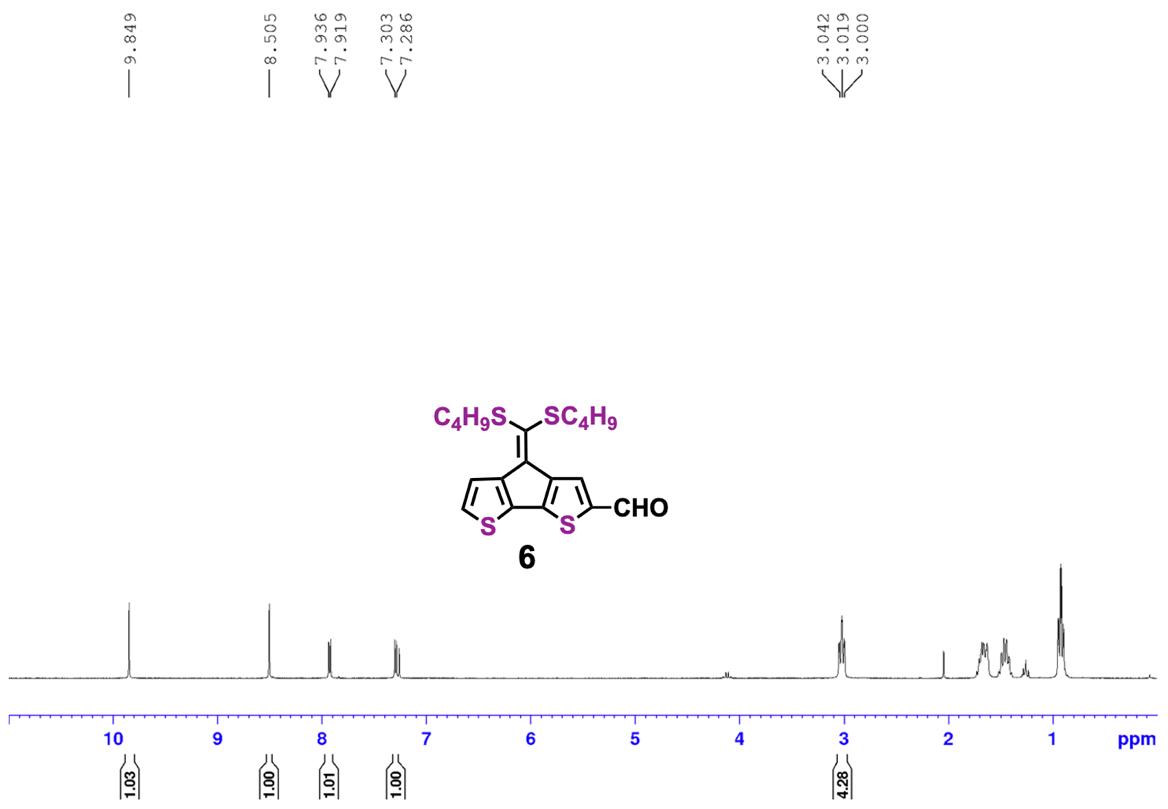


**Figure S6.** ^1^H NMR spectrum of **6** in CDCl_3_.


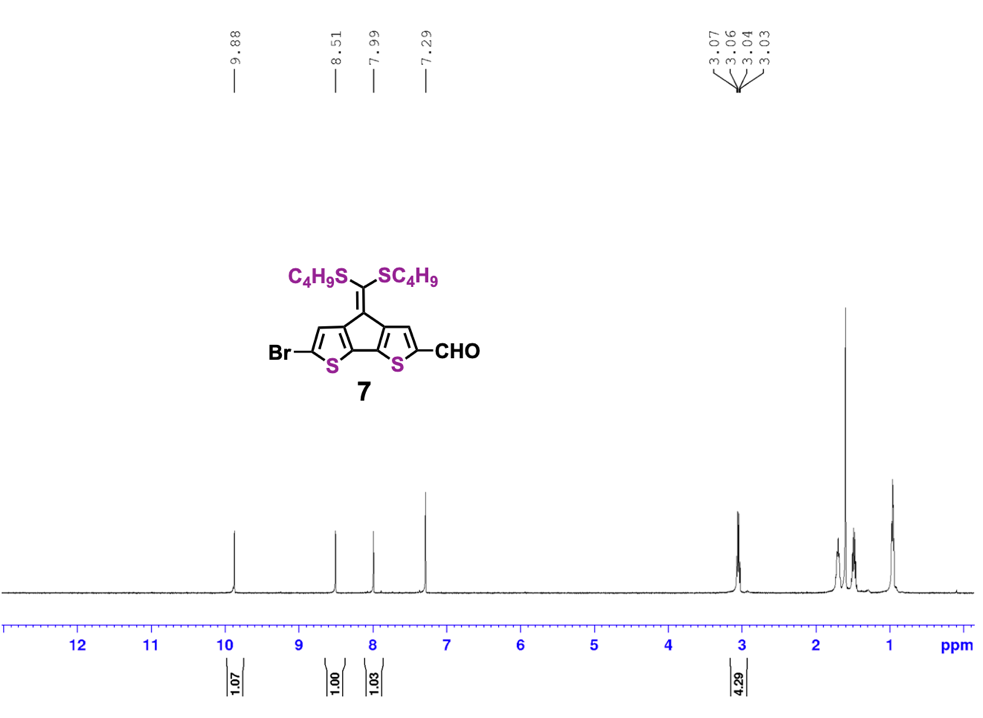


**Figure S7.** ^1^H NMR spectrum of **7** in CDCl_3_.


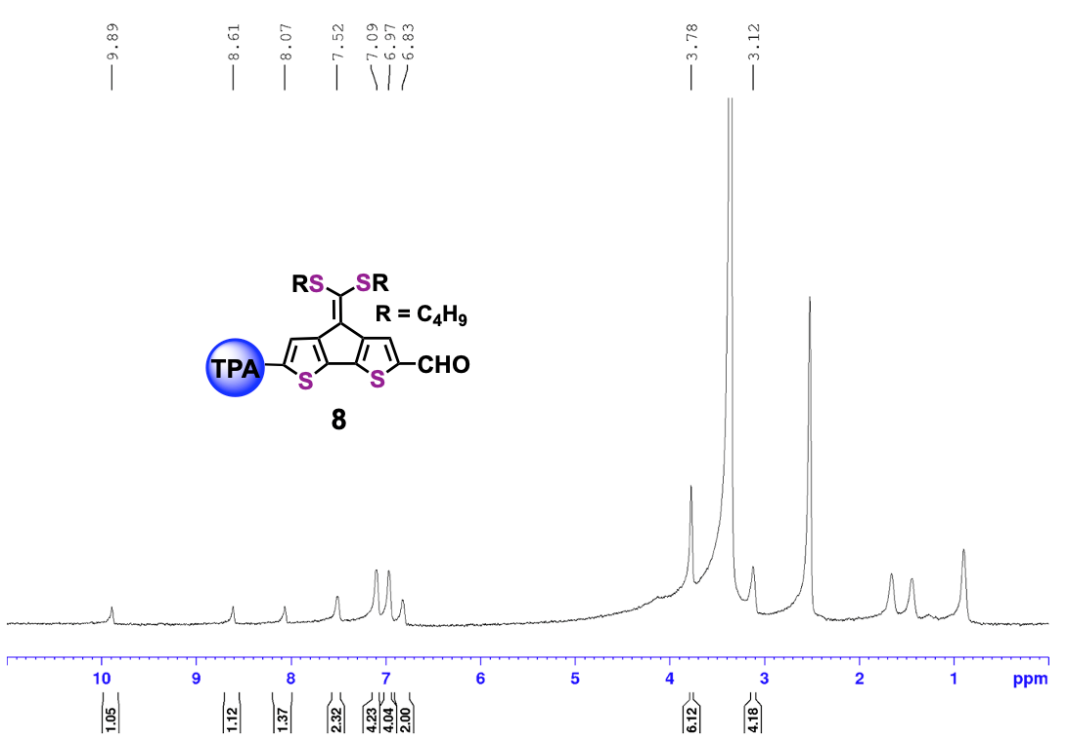


**Figure S8.** ^1^H NMR spectrum of **8** in DMSO-d6.


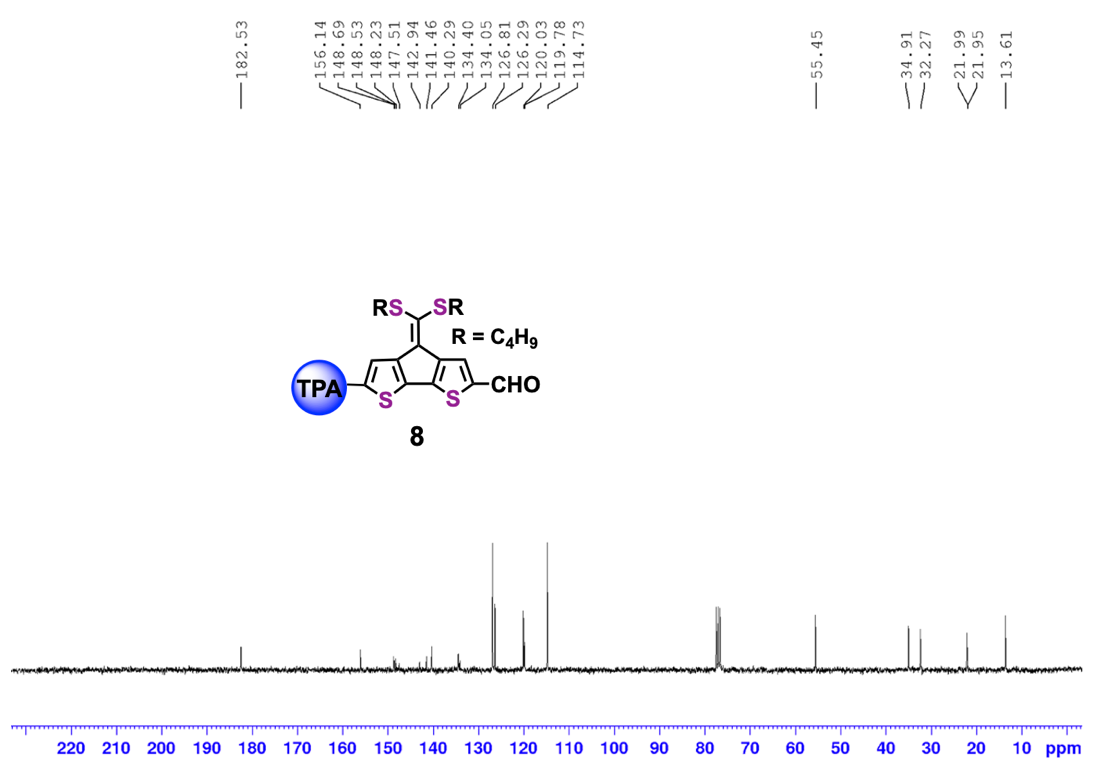


**Figure S9.** ^13^C NMR spectrum of **8** in CDCl_3_.


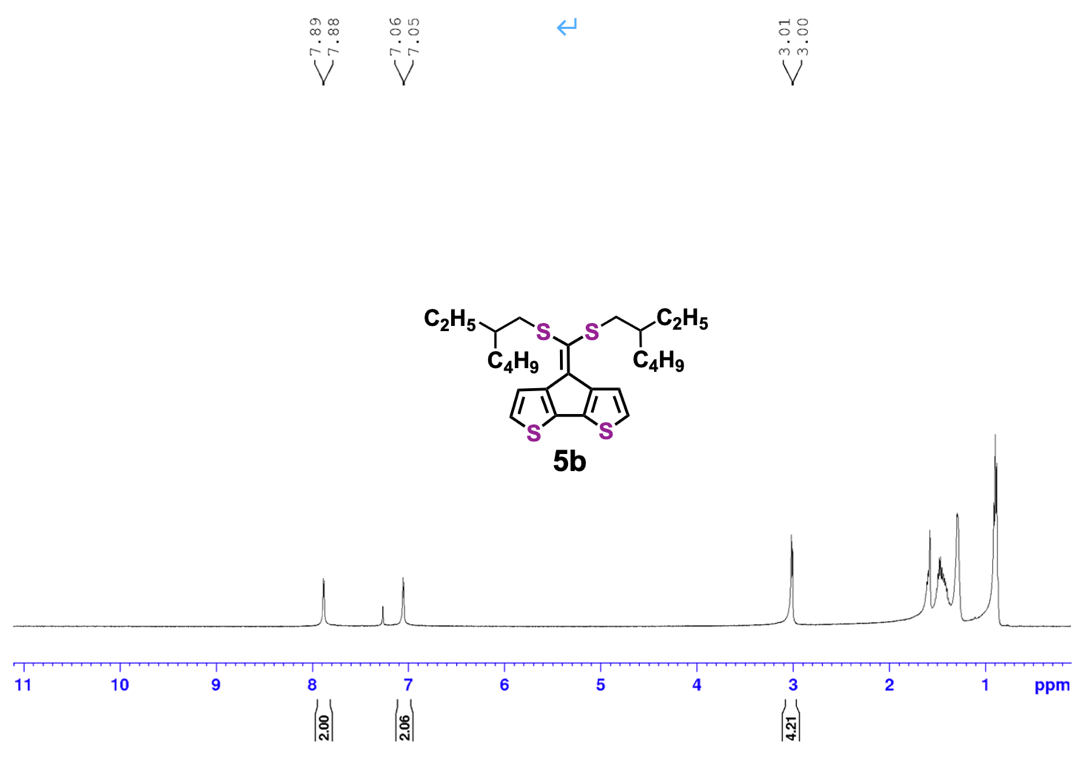


**Figure S10.** ^1^H NMR spectrum of **5b** in CDCl_3_.


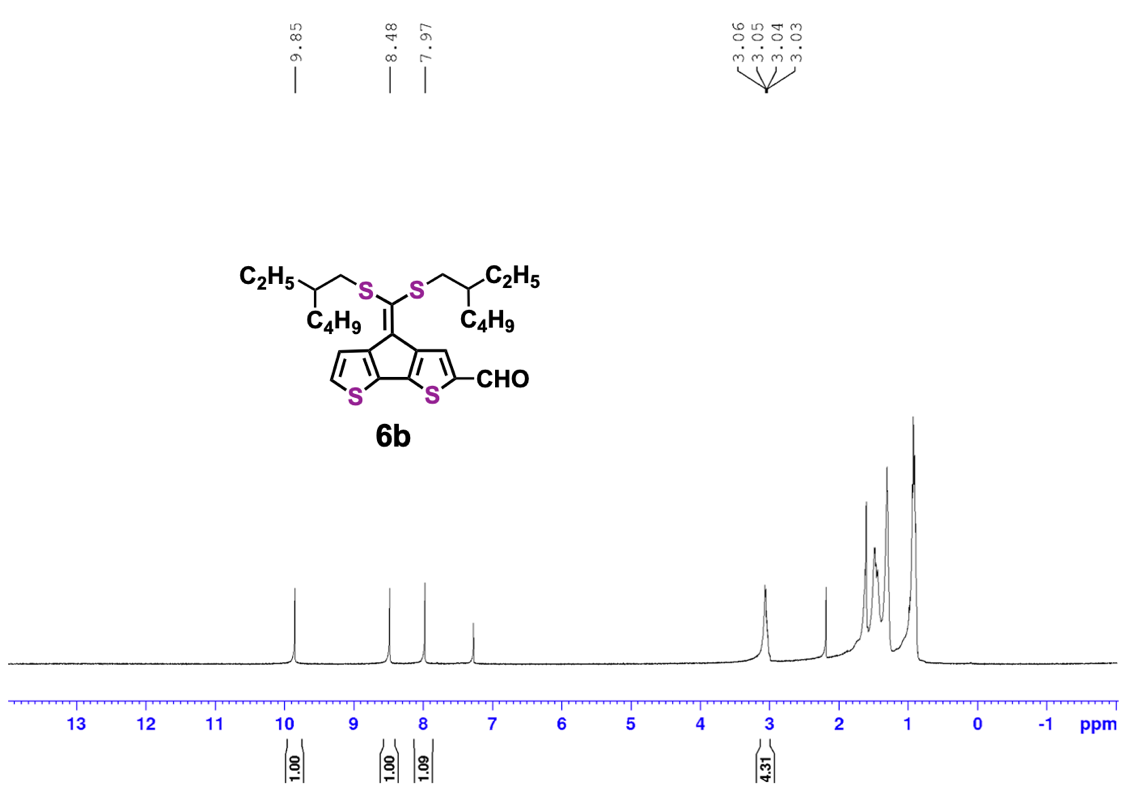


**Figure S11.** ^1^H NMR spectrum of **6b** in CDCl_3_.


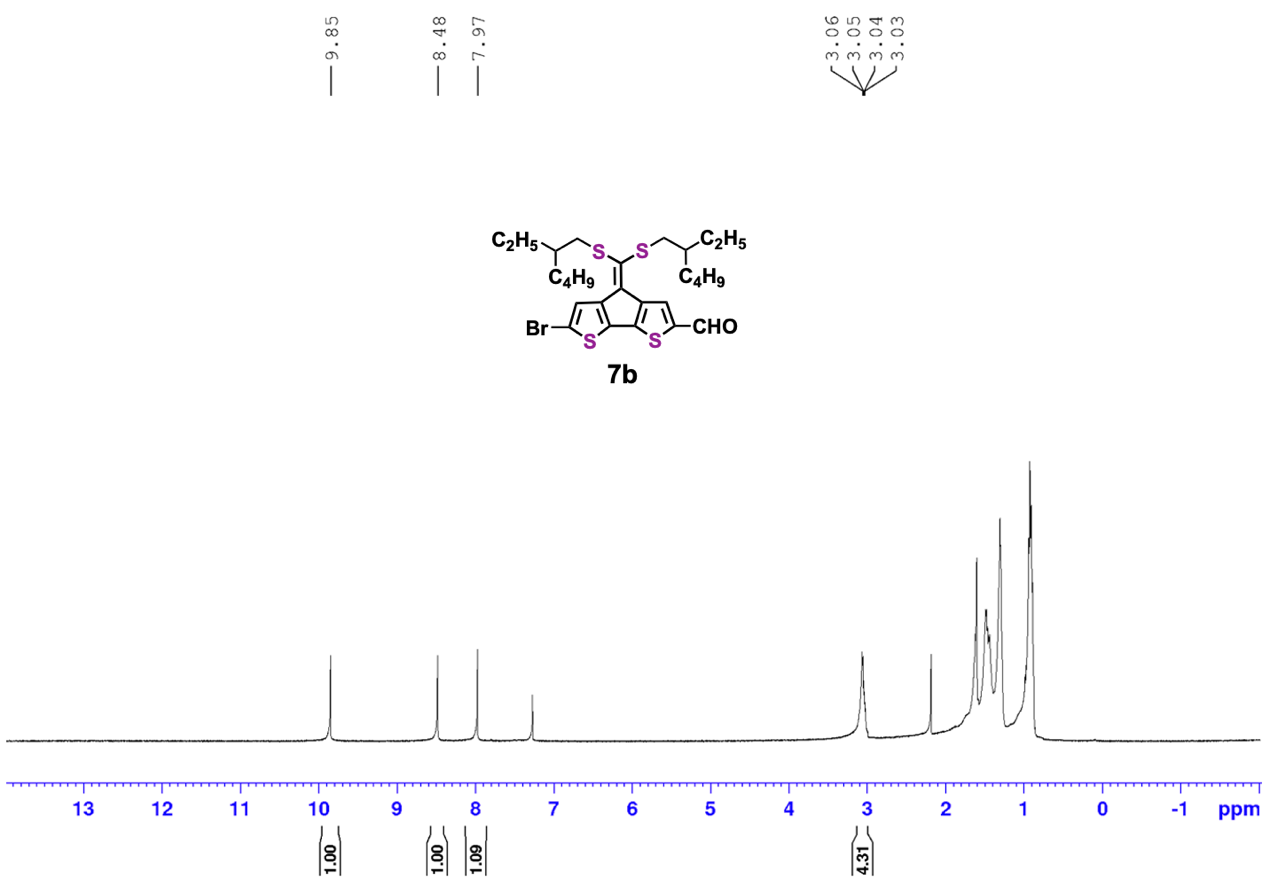


**Figure S12.** ^1^H NMR spectrum of **7b** in CDCl_3_.


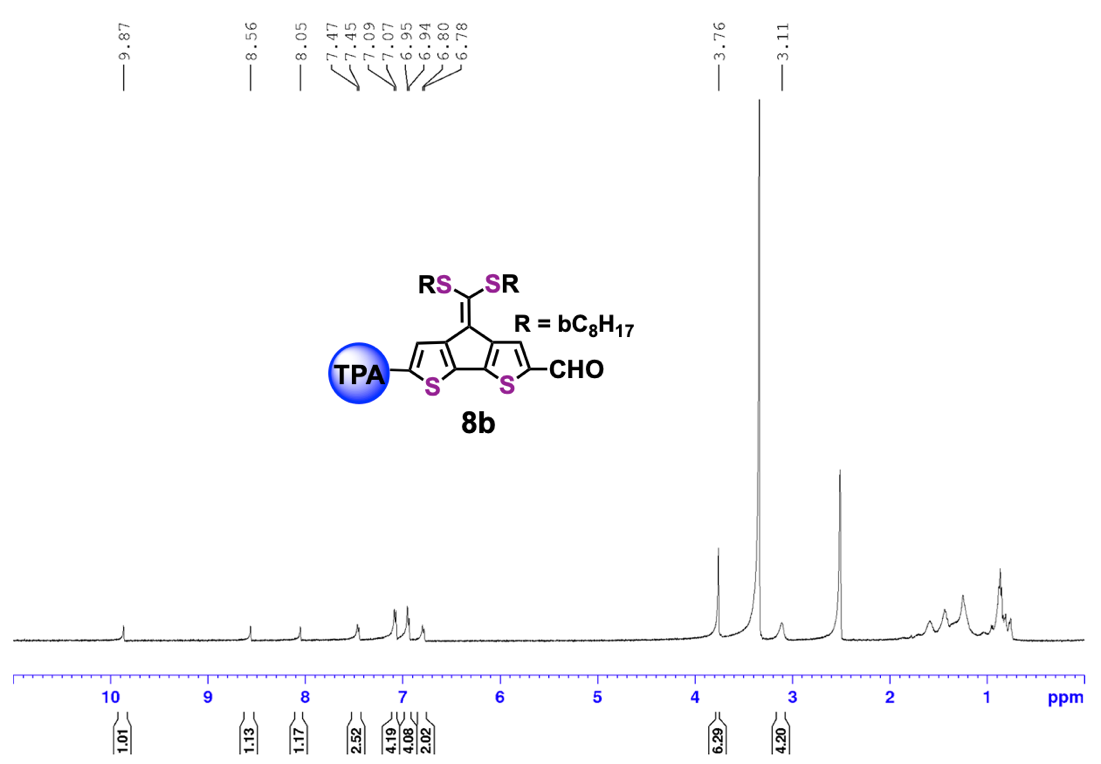


**Figure S13.** ^1^H NMR spectrum of **8b** in DMSO-d6.


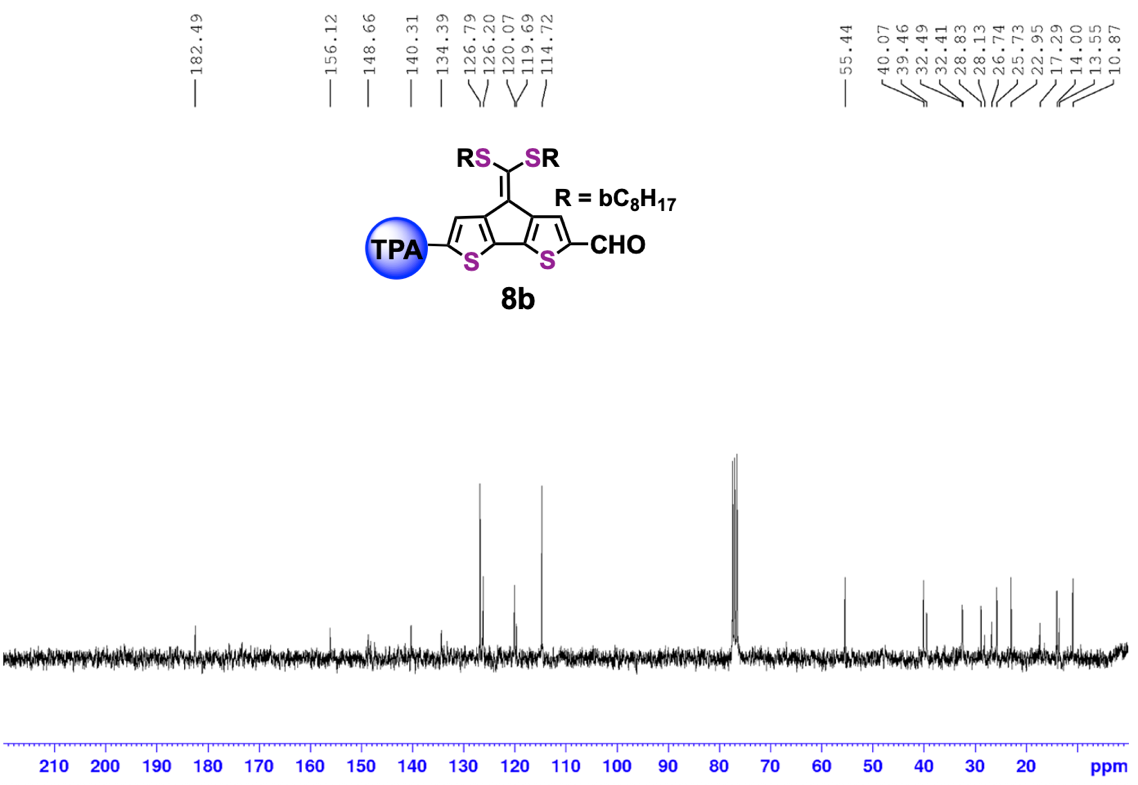


**Figure S14.** ^13^C NMR spectrum of **8b** in CDCl_3_.


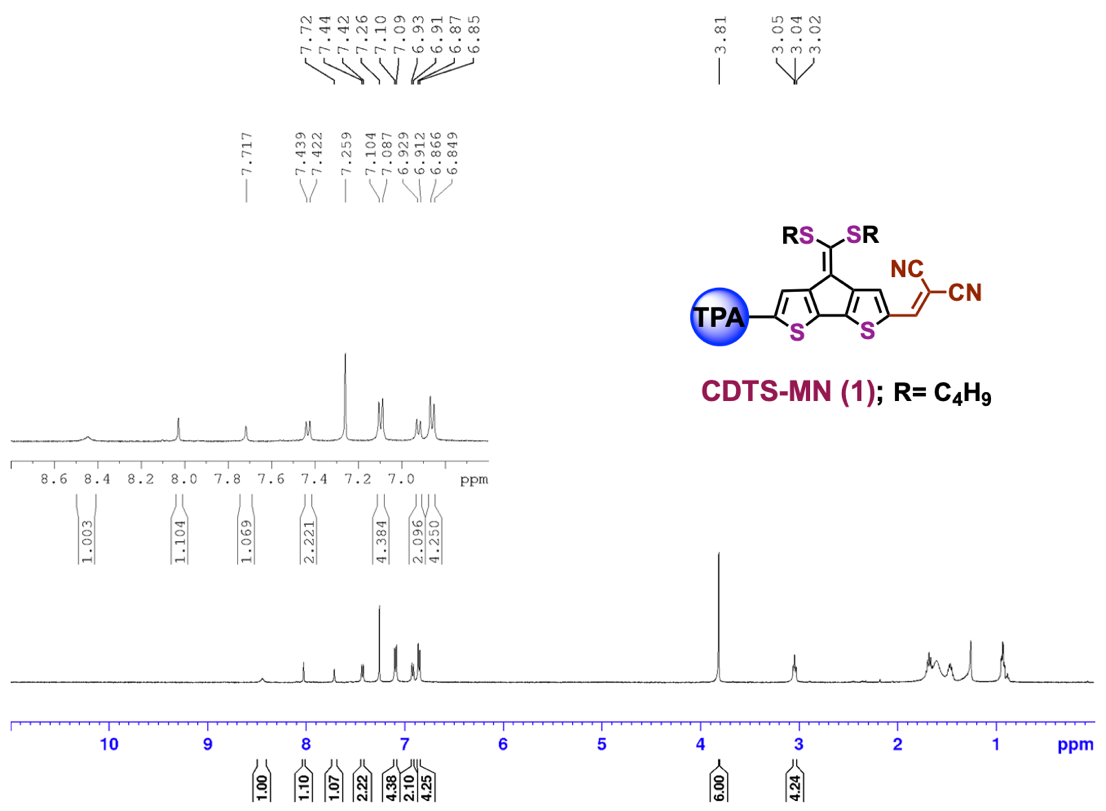


**Figure S15.** ^1^H NMR spectrum of **CDTS-MN (1)** in CDCl_3_.


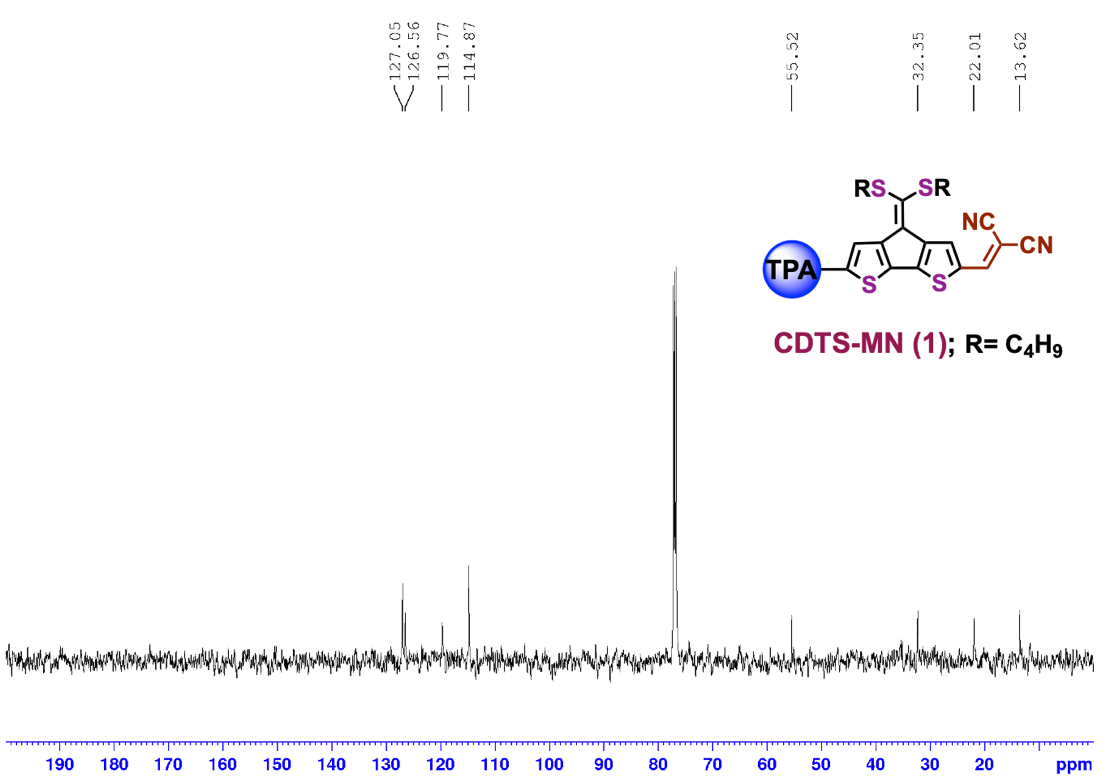


**Figure S16.** ^13^C NMR spectrum of **CDTS-MN (1)** in CDCl_3_.


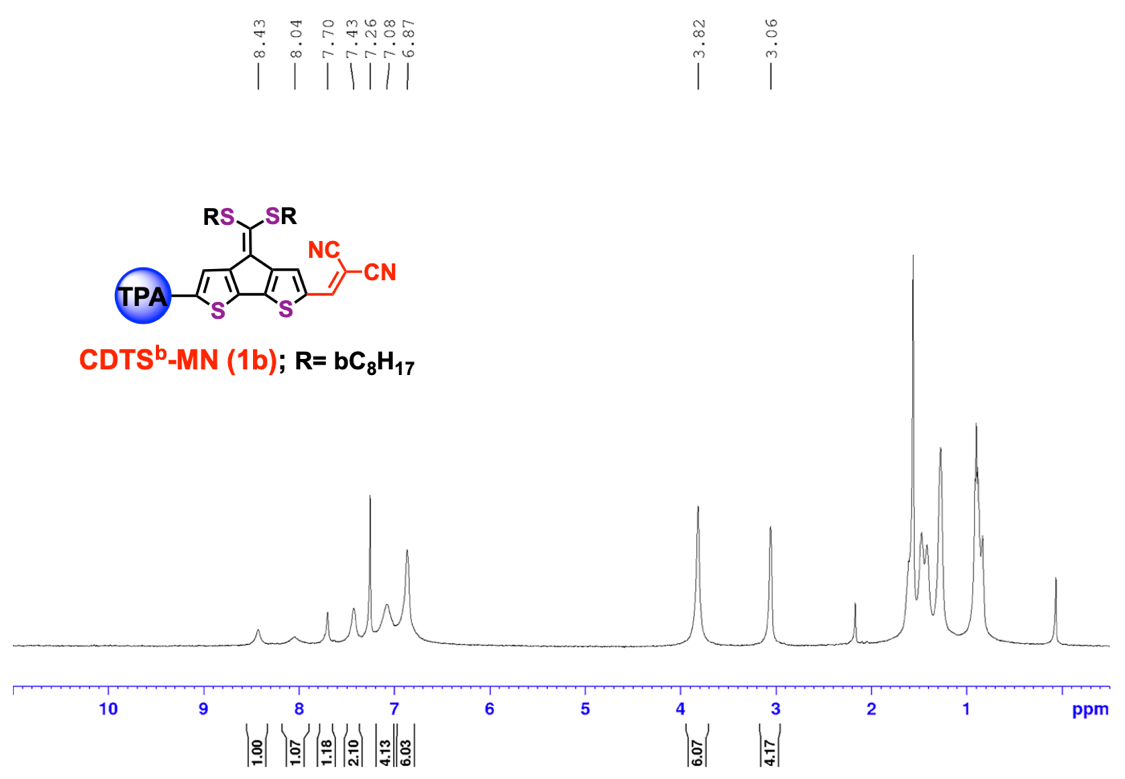


**Figure S17.** ^1^H NMR spectrum of **CDTS^b^-MN (1b)** in CDCl_3_.


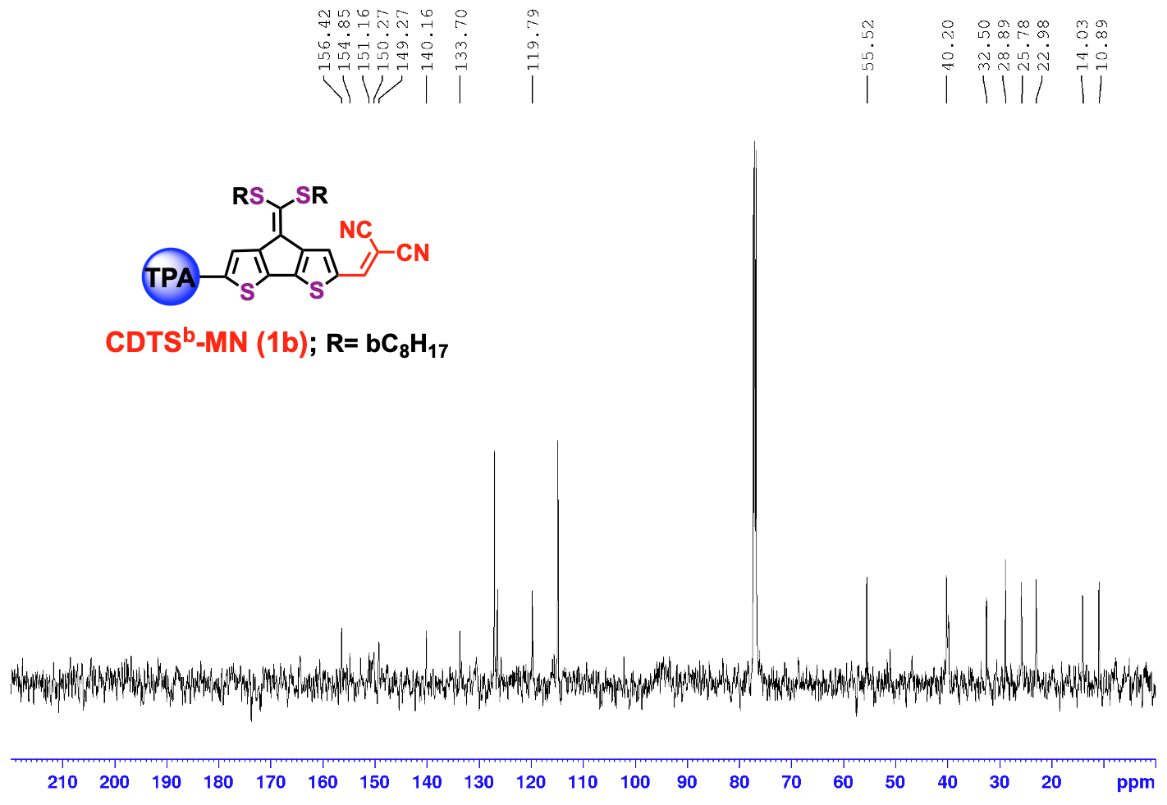


**Figure S18.** ^13^C NMR spectrum of **CDTS^b^-MN (1b)** in CDCl_3_.


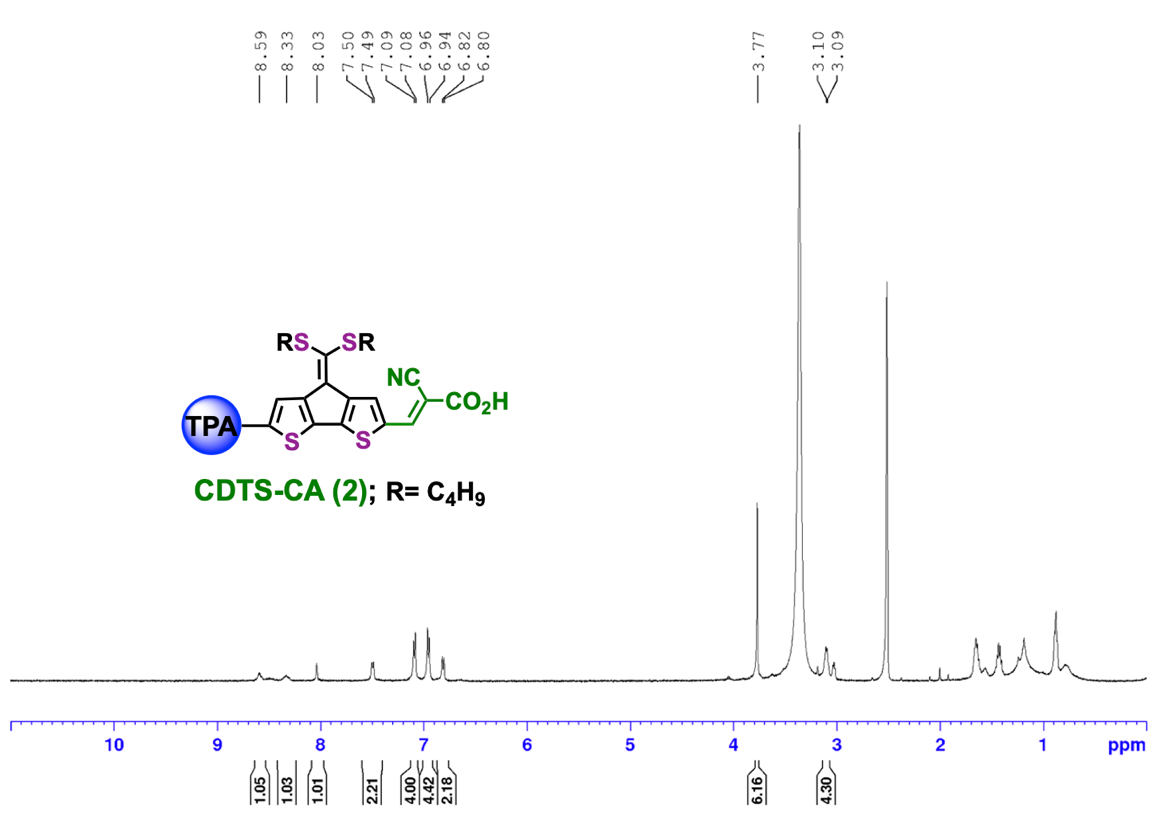


**Figure S19.** ^1^H NMR spectrum of **CDTS-CA (2)** in DMSO-d6.


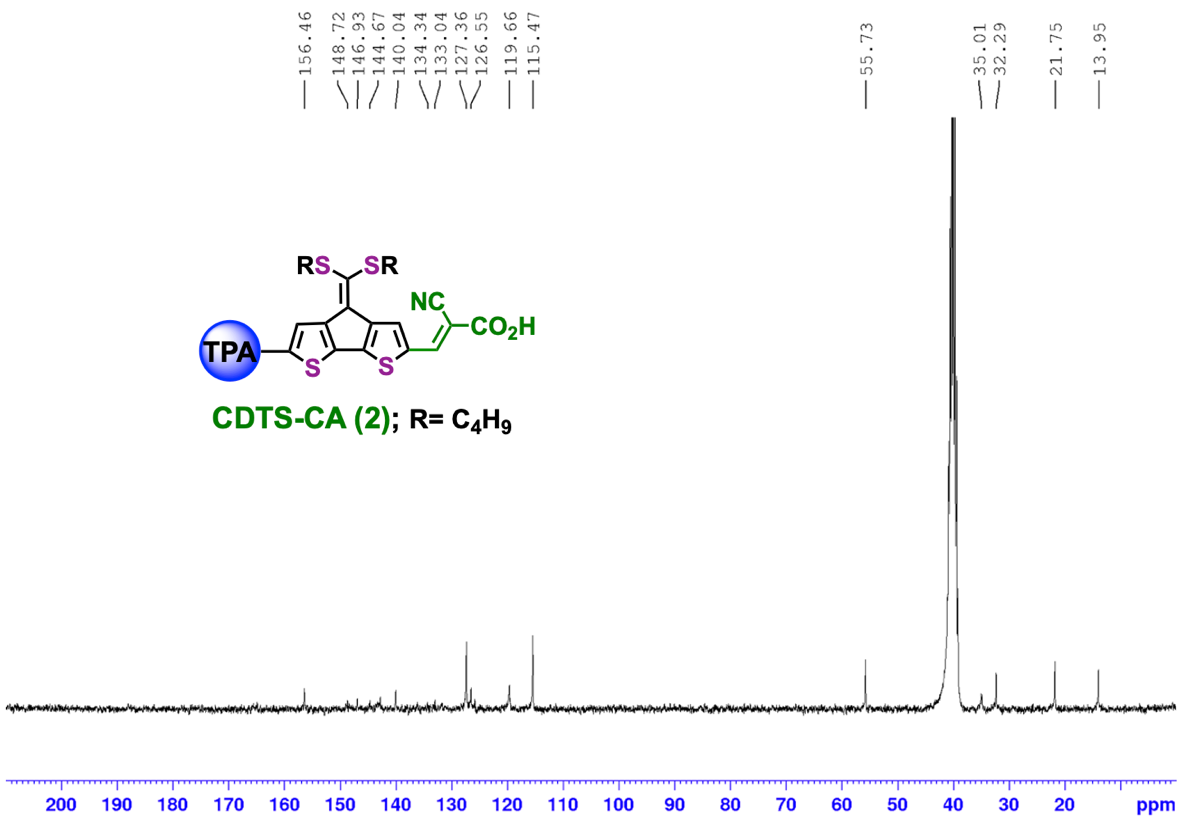


**Figure S20.** ^13^C NMR spectrum of **CDTS-CA (2)** in DMSO-d6.


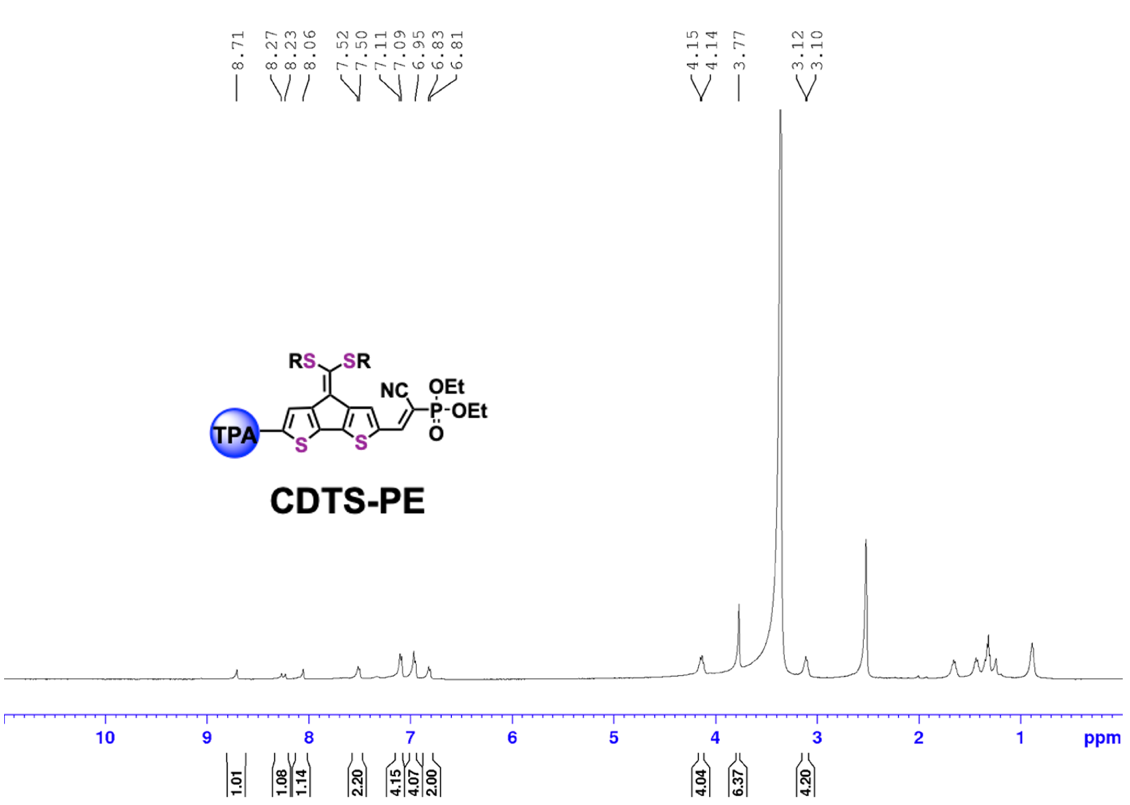


**Figure S21.** ^1^H NMR spectrum of **CDTS-PE** in DMSO-d6.


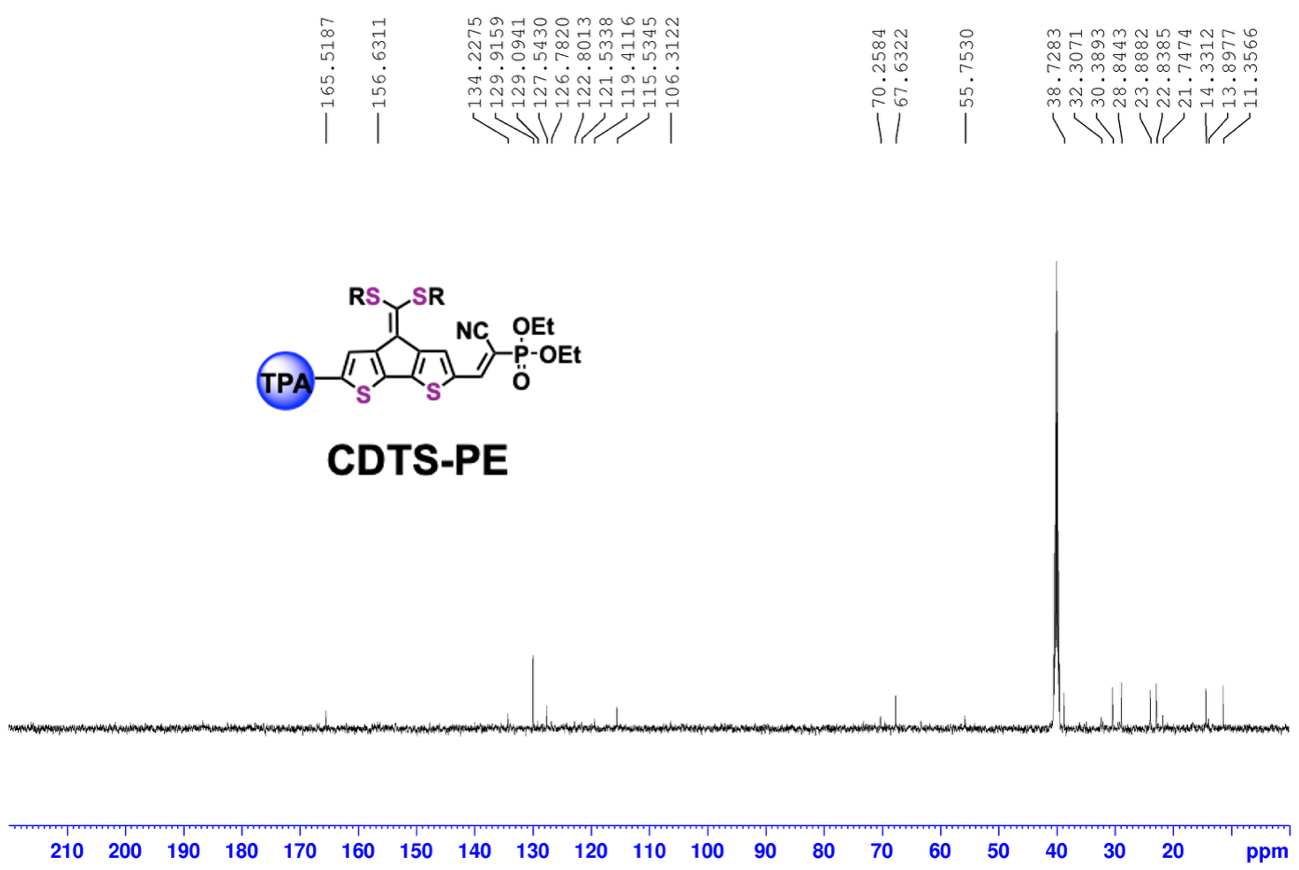
**Figure S22.** ^13^C NMR spectrum of **CDTS-PE** in DMSO-d6.


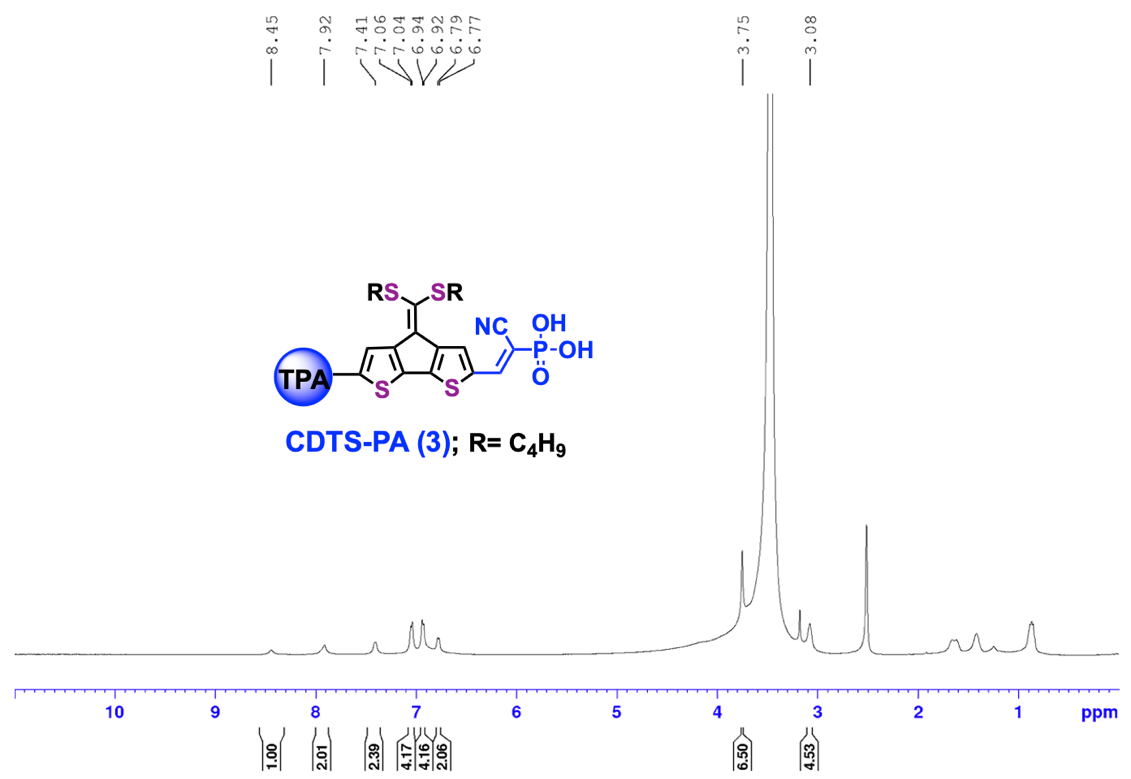


**Figure S23.** ^1^H NMR spectrum of **CDTS-PA (3)** in DMSO-d6.


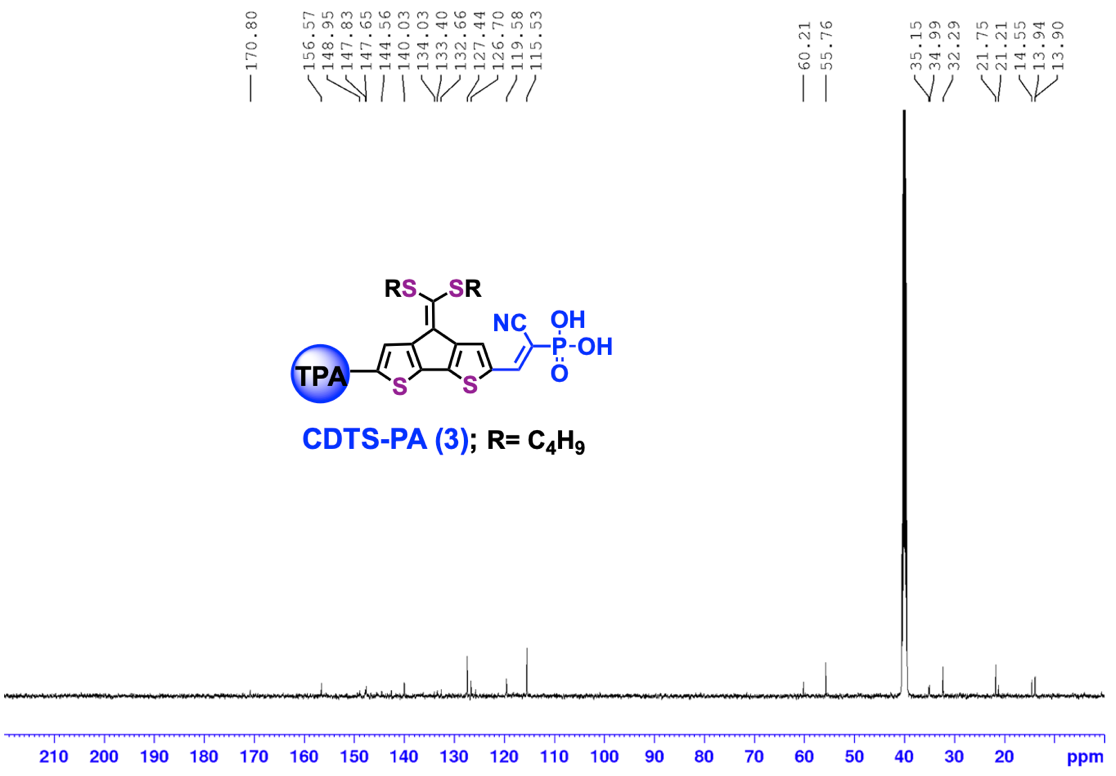


**Figure S24.** ^13^C NMR spectrum of **CDTS-PA (3)** in DMSO-d6.


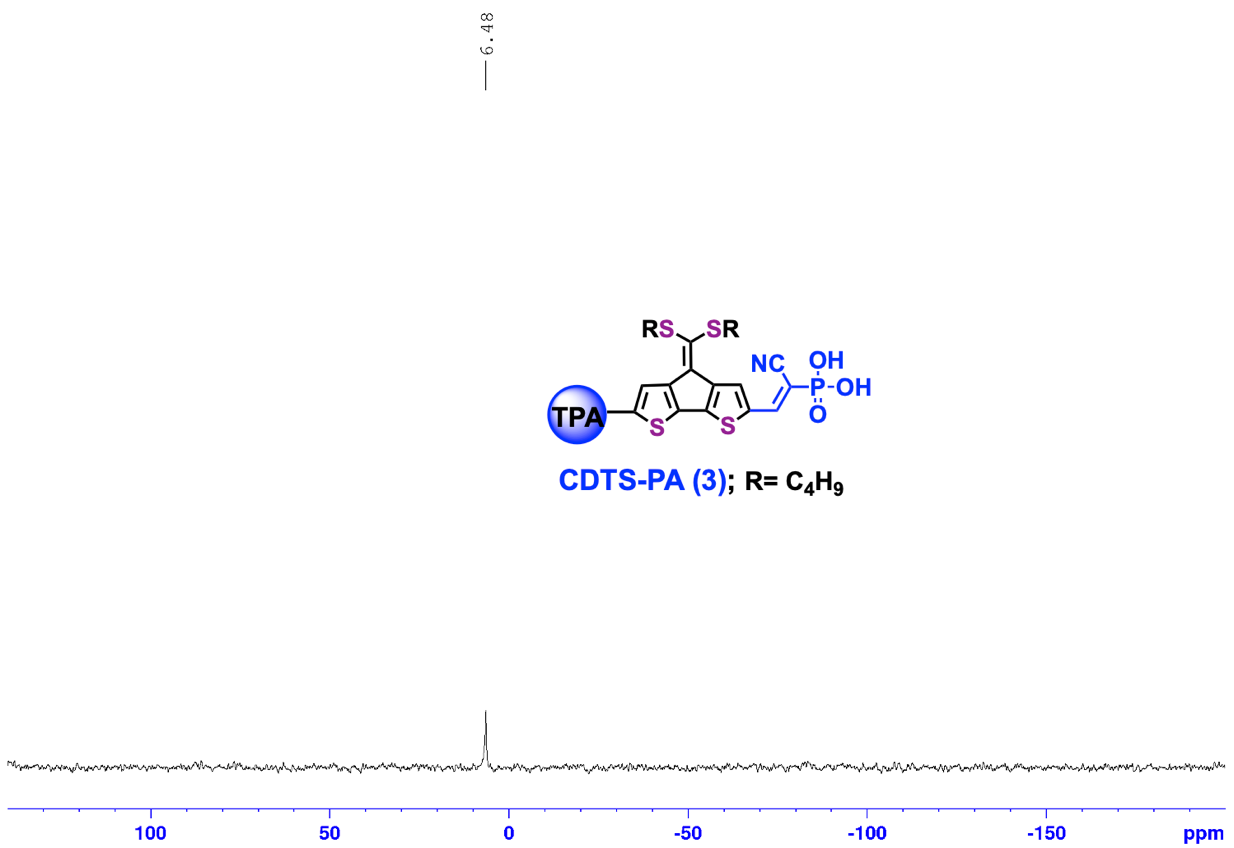


**Figure S25.** ^31^P NMR spectrum of **CDTS-PA (3)** in DMSO-d6.


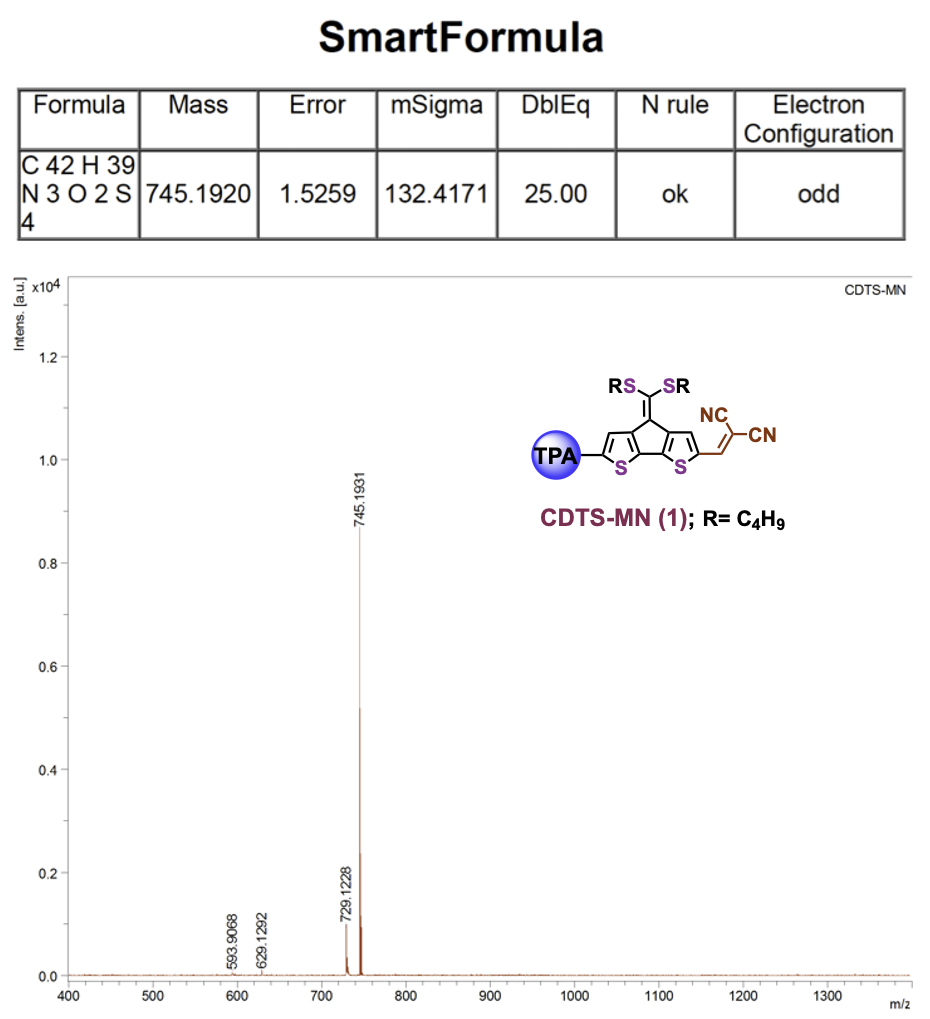


| **Figure S26.** HRMS spectrum of **CDTS-MN (1)**.  **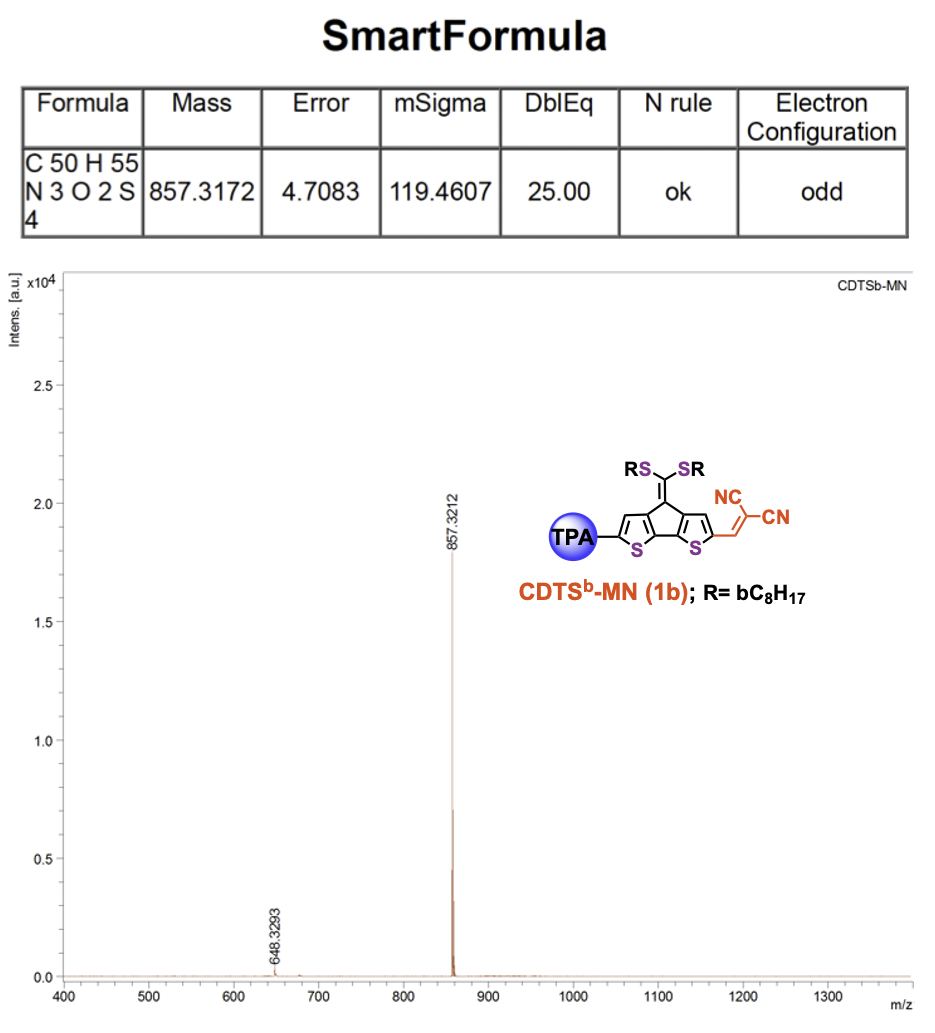**  **Figure S27.** HRMS spectrum of **CDTS^b^-MN (1b)**.  **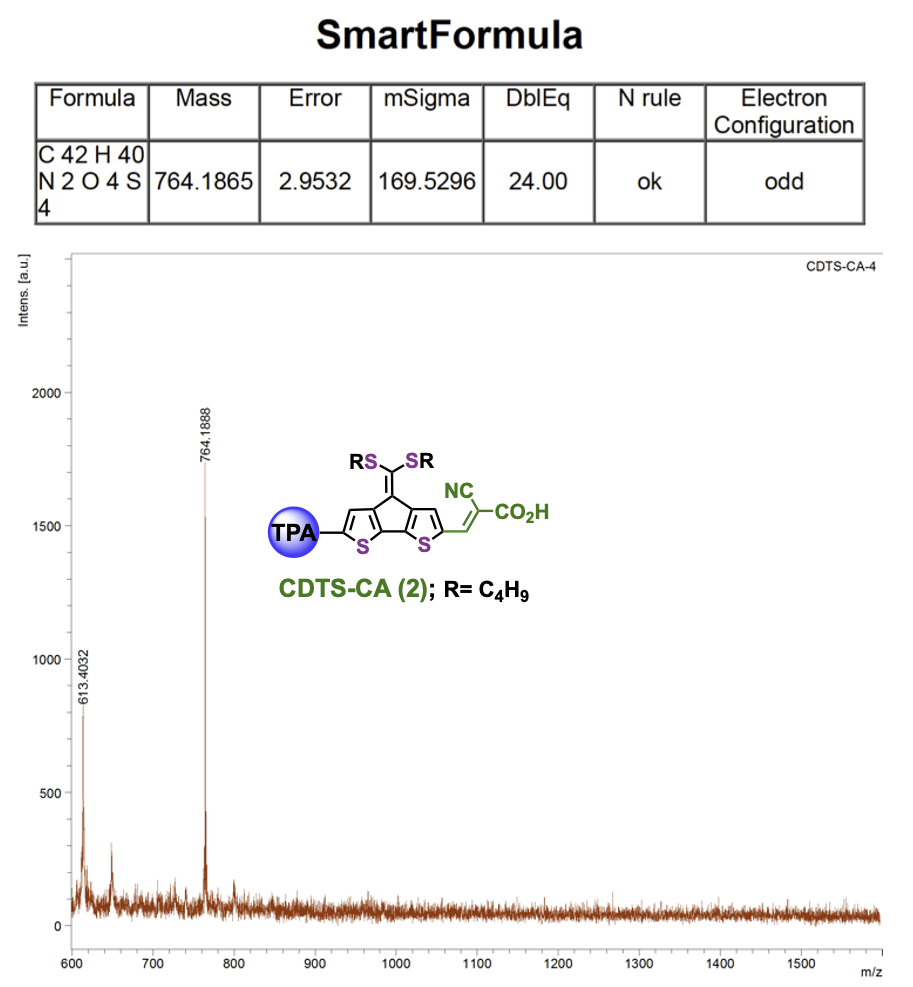**  **Figure S28.** HRMS spectrum of **CDTS-CA (2)**.  **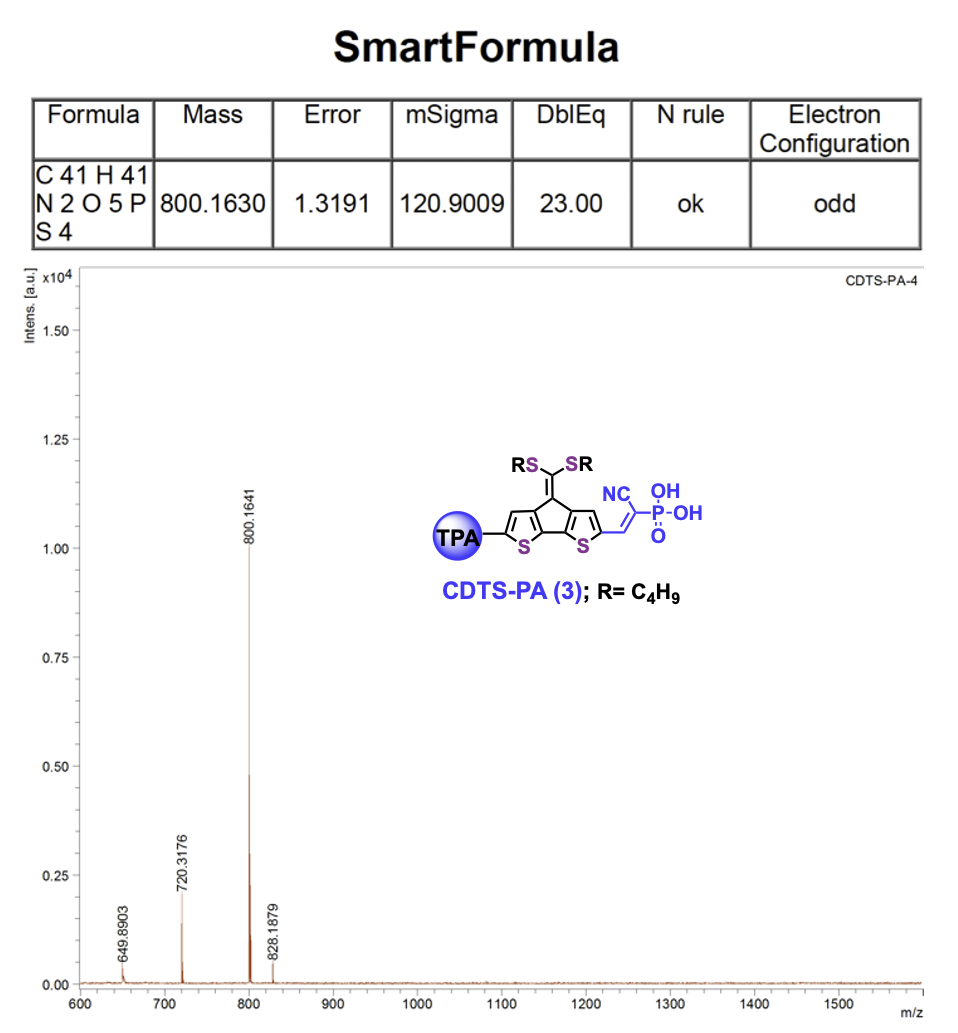**  **Figure S29.** HRMS spectrum of **CDTS-PA (3)**.  ***DFT Analysis***  To explore the electronic structures of CDTS-SAMs, DFT calculations were performed at the B3LYP/6-31G* level of theory with the Gaussian 09W software (**Figure S30**). The HOMOs of the SAM molecules are primarily located on the triphenylamine units, while the LUMOs are situated on the dithioalkylated methylidenyl-CDT unit and its associated anchoring group. DFT-derived E_HOMO_ and E_LUMO_ of molecules **1−3** are located at −4.82/-2.71 eV (**1**), −4.84/2.76 eV (**1b**), −4.80/-2.68 eV (**2**), -4.72/-2.52 eV (**3**), respectively. It is noteworthy that the energy gaps obtained through computational, optical, and electrochemical methods are quite similar, demonstrating the alignment between the experimental results and computational predictions. To investigate the electronic structures of CDTS-SAMs, electrostatic surface potential (ESP) analysis of the CDTS-based SAMs was conducted to investigate the molecular charge distribution and assess the interactions between the NiOx/ITO surface and the anchoring group sites (**Figure S30**). The ESP mapping images for the three CDTS-based SAMs reveal a high density of negative charges, primarily concentrated on the dicyanomethylene, cyanoacrylic acid, and phosphonic acid functionalities. This distribution of negative charge promotes the formation of SAMs on the NiOx/ITO surface by fostering strong interactions with the anchoring groups, potentially improving charge transport at the SAM/NiOx interface in TPSCs.^[4-5]^  **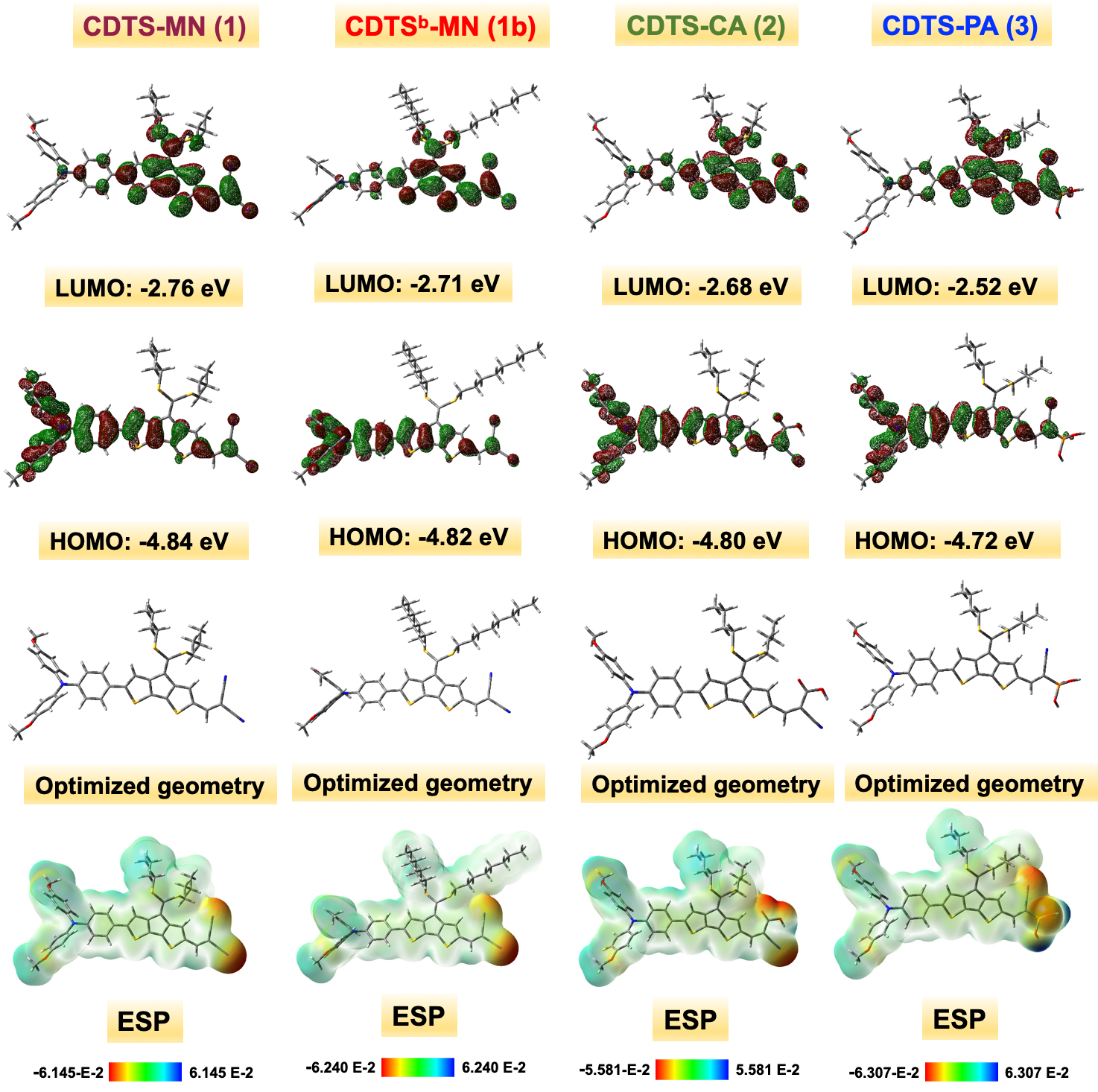**  **Figure S30.** Energy levels derived from DFT calculations, and ESP mapping for molecules **1**–**3**.  **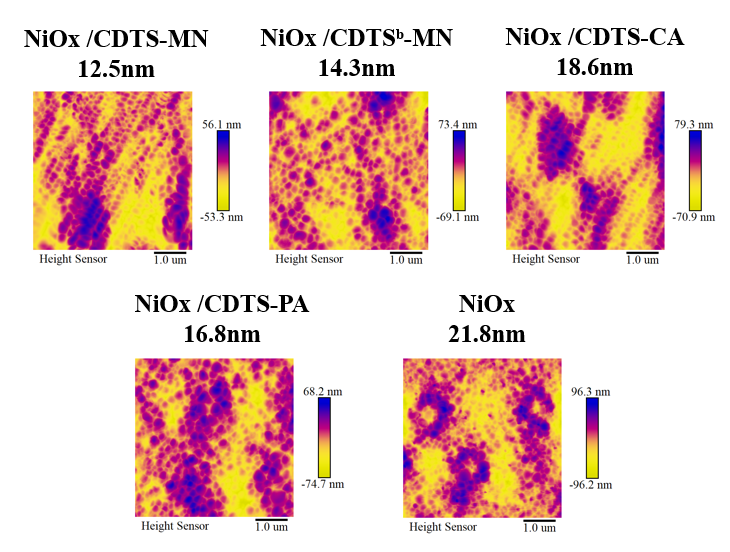**  **Figure S31.** AFM 2D-images of tin perovskite made by two steps for varied SAMs on NiOx  ~~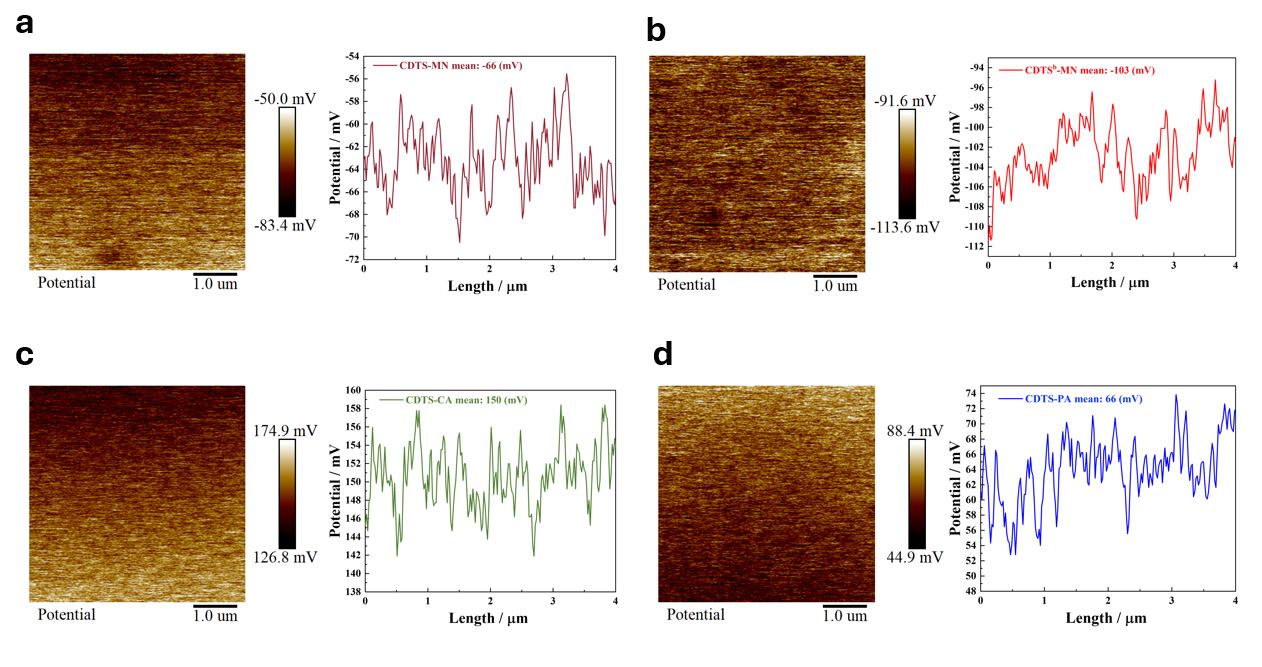~~  **Figure S32**. KPFM images and line profile of CDTS-based SAM deposited on NiOx surfaces (a) **CDTS-MN** (b) **CDTSᵇ-MN** (c) **CDTS-CA** (d) **CDTS-PA**  **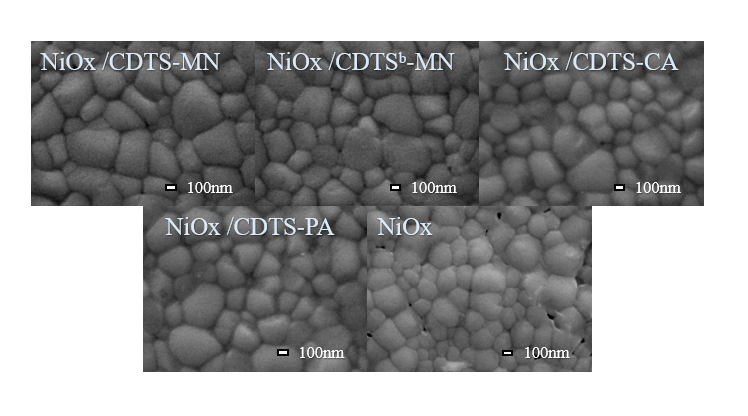**  **Figure S33.** SEM Top view images of tin perovskite on varied NiOx/SAM films.  **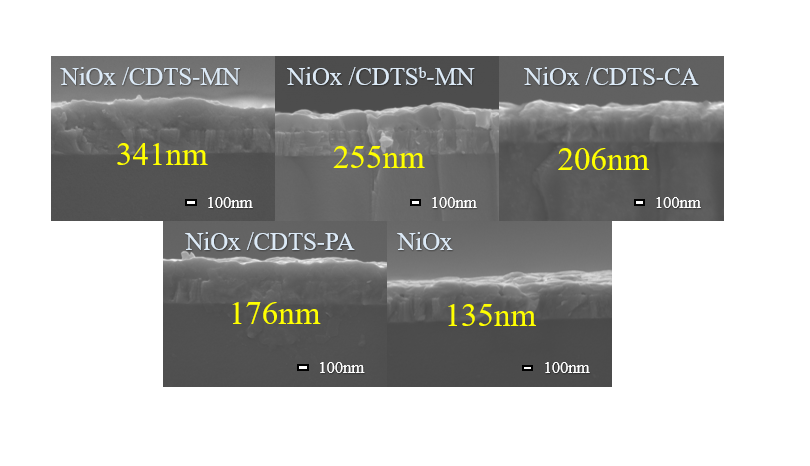**  **Figure S34.** SEM cross section image of tin perovskite with (a) NiOx/**CDTS-MN**; (b) NiOx/ **CDTS^b^-MN**; (c) NiOx/**CDTS-CA**; (d) NiOx/**CDTS-PA**; (e) NiOx-only deposited on ITO substrates.  **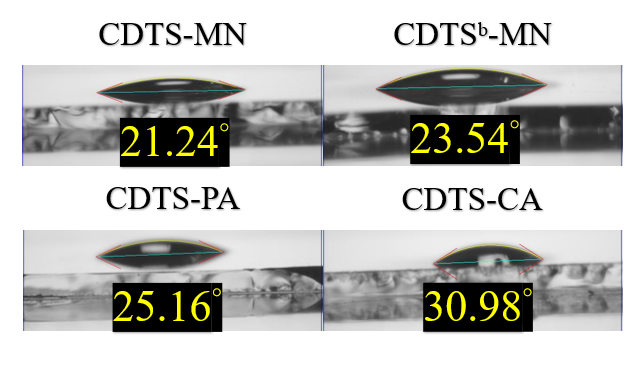**  **Figure S35.** Precursor solution contact angles on varied **CDTS**-SAM films deposited on ITO substrates.  **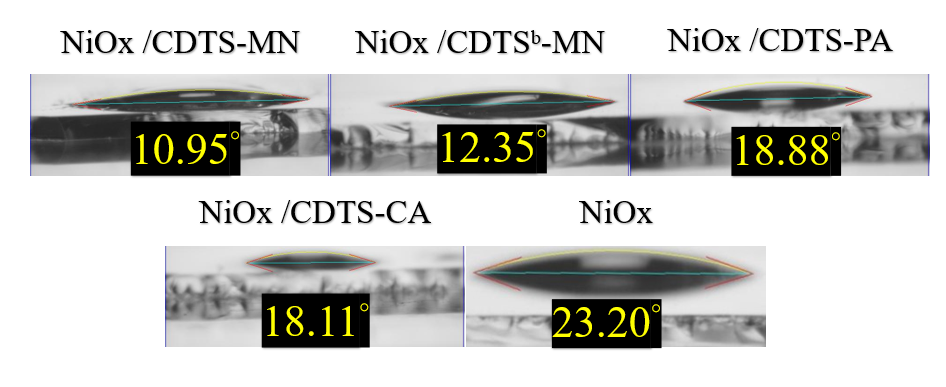**  **Figure S36.** Precursor solution contact angles on **CDTS**-SAMs films deposited on NiOx/ITO substrates in comparison with NiOx-only/ITO film.  **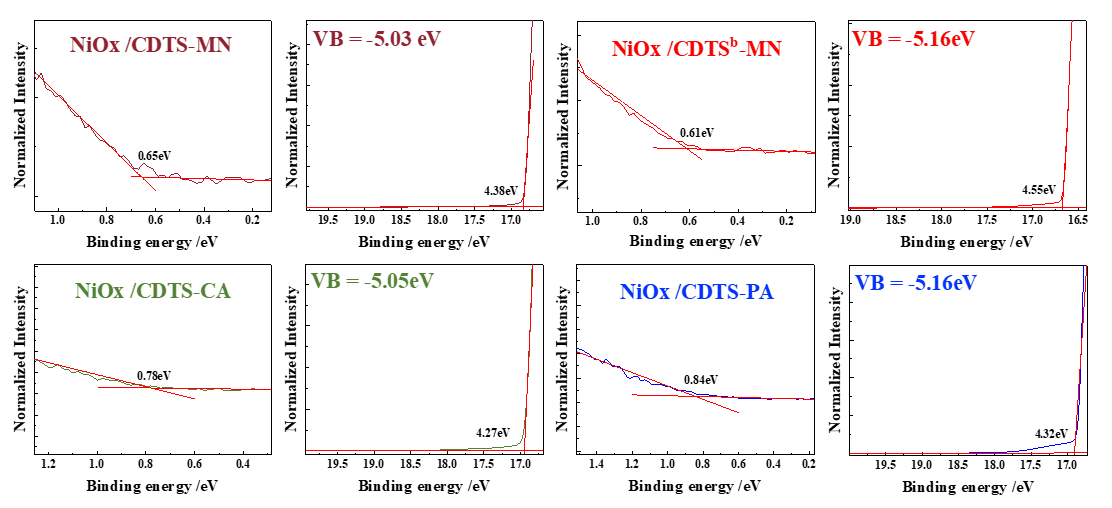**  **Figure S37.** UPS raw data for NiOx/SAM films deposited on ITO substrates.  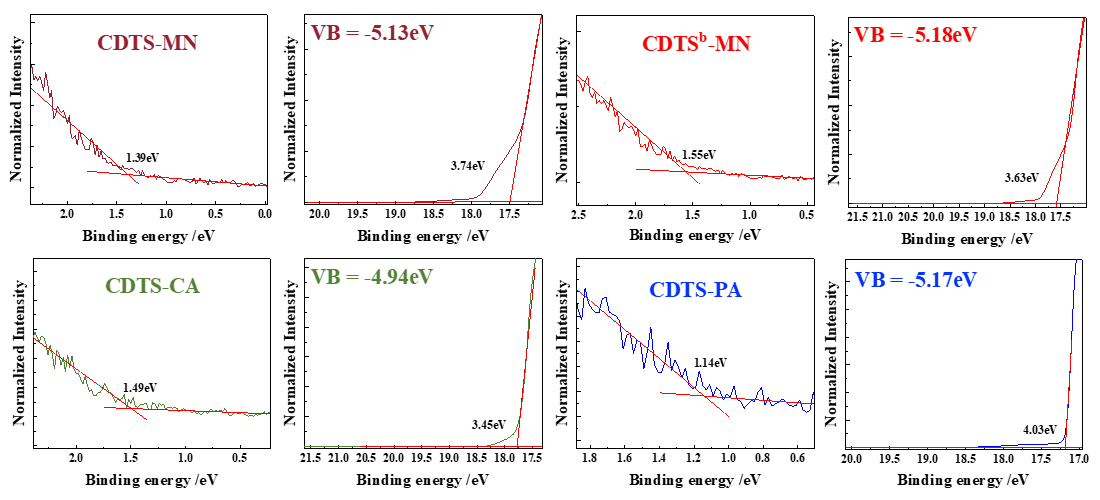  **Figure S38.** UPS raw data for CDTS-based series SAM films deposited on ITO substrates.  **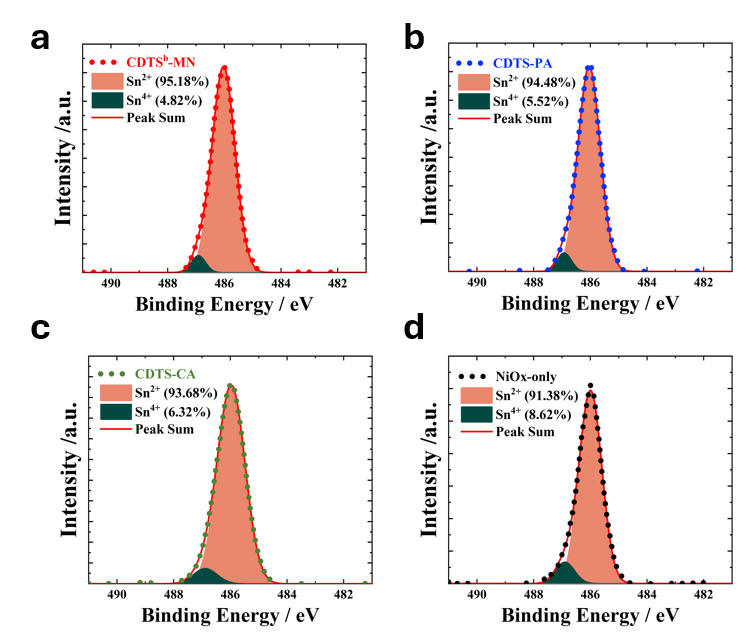**  **Figure S39**. Sn XPS spectra of perovskite film deposited on (a) NiOx/**CDTS^b^-MN** (b) NiOx/**CDTS-PA** (c) NiOx/**CDTS-CA** (d)NiOx-only.  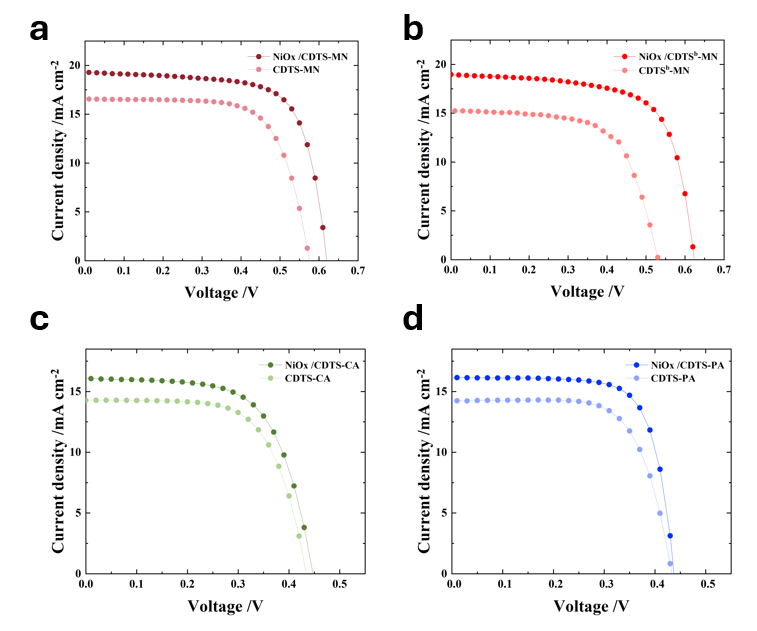  **Figure S40** Device performances of tin perovskite deposited on SAM/ITO substrates (lighter curves) and SAM/NiOx/ITO substrates (darker curves) are presented for (a) CDTS-MN, (b) CDTS-bMN, (c) CDTS-CA, and (d) CDTS-PA.  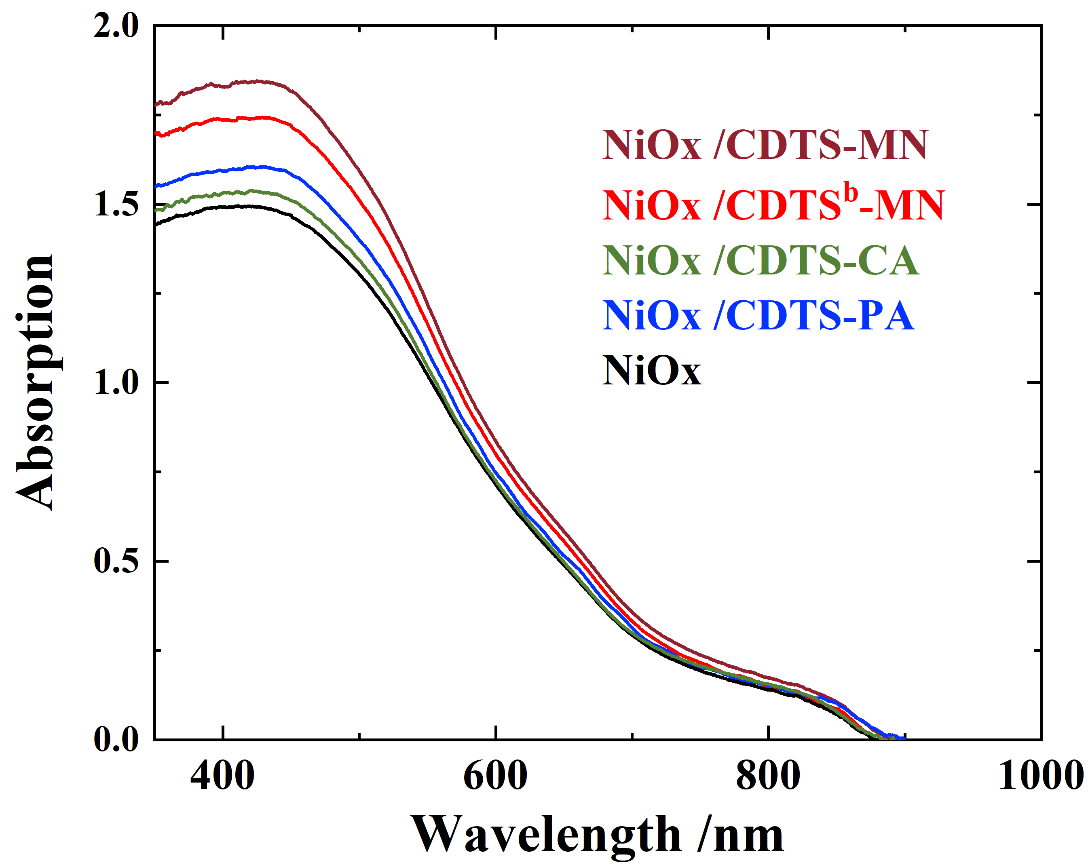  **Figure S41.**  UV/Vis diagram of tin perovskites on NiOx /SAMs as depicted.  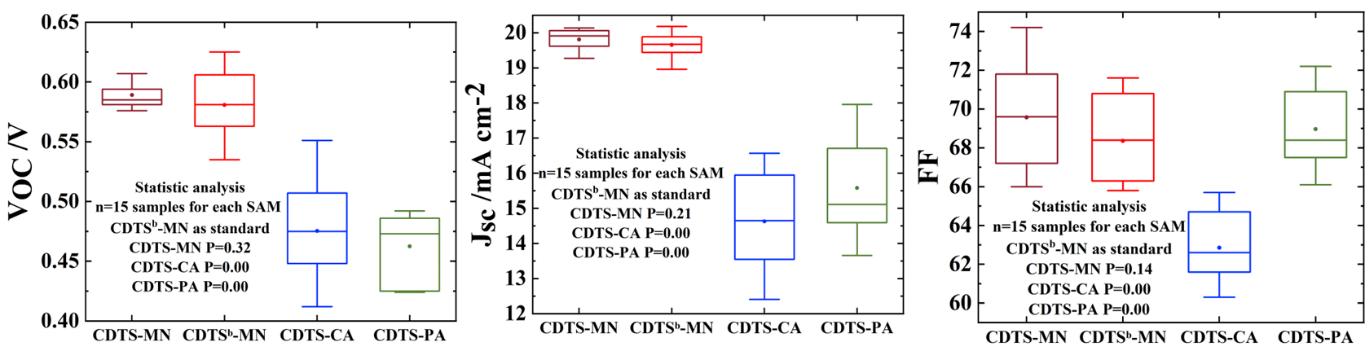  **Figure S42.** Boxplots of *V*oc/*J*sc/FF with different SAM–functionalized tin perovskite devices deposited on NiOx as indicated.  **Table S1. Crystal and structural refinement data of CDTS-MN (1) & CDTS^b^-MN (1b)**   \|  \| **CDTS-MN** \| **CDTS^b^-MN** \| \| --- \| --- \| --- \| \| Identification code \| 240813lt_twin1_hklf4 \| 240717lt_auto \| \| Empirical formula \| C_42_H_39_N_3_O_2_S2 \| C_50_H_55_N_3_O_2_S2 \| \| Formula weight \| 746 \| 858.21 \| \| Temperature/K \| 100.00(11) \| 99.99(10) \| \| Crystal system \| monoclinic \| triclinic \| \| Space group \| P2_1_/n \| P-1 \| \| a/Å \| 10.39420(10) \| 9.61200(10) \| \| b/Å \| 29.0707(2) \| 14.0983(2) \| \| c/Å \| 25.0557(2) \| 17.2064(2) \| \| α/° \| 90 \| 81.0050(10) \| \| β/° \| 93.2260(10) \| 77.6610(10) \| \| γ/° \| 90 \| 85.4030(10) \| \| Volume/Å^3^ \| 7559.00(11) \| 2247.17(5) \| \| Z \| 8 \| 2 \| \| ρ_calc_g/cm^3^ \| 1.311 \| 1.268 \| \| μ/mm^‑1^ \| 2.626 \| 2.273 \| \| F(000) \| 3136 \| 912 \| \| Crystal size/mm^3^ \| 0.19 × 0.04 × 0.02 \| 0.09 × 0.03 × 0.02 \| \| Radiation \| Cu Kα (λ = 1.54184) \| Cu Kα (λ = 1.54184) \| \| 2Θ range for data collection/° \| 7.034 to 149.364 \| 7.68 to 146.304 \| \| Index ranges \| -11 ≤ h ≤ 12, -35 ≤ k ≤ 35, -30 ≤ l ≤ 30 \| -11 ≤ h ≤ 8, -17 ≤ k ≤ 17, -21 ≤ l ≤ 21 \| \| Reflections collected \| 26377 \| 34130 \| \| Independent reflections \| 26377 [R_int_ = ?, R_sigma_ = 0.0106] \| 8716 [R_int_ = 0.0254, R_sigma_ = 0.0248] \| \| Data/restraints/parameters \| 26377/200/1005 \| 8716/423/679 \| \| Goodness-of-fit on F^2^ \| 1.043 \| 1.069 \| \| Final R indexes [I>=2σ (I)] \| R_1_ = 0.0395, wR_2_ = 0.1081 \| R_1_ = 0.0365, wR_2_ = 0.0952 \| \| Final R indexes [all data] \| R_1_ = 0.0436, wR_2_ = 0.1108 \| R_1_ = 0.0433, wR_2_ = 0.0990 \| \| Largest diff. peak/hole / e Å^-3^ \| 0.47/-0.37 \| 0.46/-0.34 \|   **Table S2**. Thermal, optical, and electrochemical properties of the indicated molecules   \| **SAM** \| ***T*_d_^a)^ [°C]** \| ***λ*_abs_ (soln)^b)^ [nm]** \| ***E*_ox_^c)^ [V]** \| **HOMO^d)^ [eV]** \| **LUMO^e)^ [eV]** \| **△*E*_g_^f)^ [eV]** \| \| --- \| --- \| --- \| --- \| --- \| --- \| --- \| \| **CDTS-MN (1)** \| 254 \| 593 \| 0.85 \| -5.29 \| -3.56 \| 1.73 \| \| **CDTS^b^-MN (1b)** \| 316 \| 592 \| 0.86 \| -5.30 \| -3.54 \| 1.76 \| \| **CDTS-CA (2)** \| 260 \| 550 \| 0.78 \| -5.22 \| -3.43 \| 1.80 \| \| **CDTS-PA (3)** \| 268 \| 548 \| 0.70 \| -5.20 \| -3.37 \| 1.83 \|   a) By TGA. b) UV-vis absorption spectra were recorded in *o*-C_6_H_4_Cl_2_. c) By DPV in *o*-C_6_H_4_Cl_2_ at 25 °C. All potentials are reported with reference to the Fc/Fc^+^; d) E(eV) = - (4.44 + E_ox_ (vs NHE)). e) Calculated using equation; E(eV) = HOMO +△E_g_; f) Optical band gap calculated by 1240/λ_onset_.  **Table S3.** TCSPC fitting parameters of various SAMs deposited on NiOx.   \| **Samples** \| ***τ*_1_/ns *(A*_1_*%)*** \| ***τ_2_*/ns *(A_2_%)*** \| **Average Lifetime/ns** \| \| --- \| --- \| --- \| --- \| \| **NiOx/CDTS-MN** \| **0.27 (100%)** \| **One component** \| **0.27** \| \| **NiOx/CDTS^b^-MN** \| **0.55 (68.4%)** \| **1.74 (31.6%)** \| **1.25** \| \| **NiOx/CDTS-CA** \| **4.33 (79.1%)** \| **7.48 (20.9%)** \| **5.32** \| \| **NiOx/CDTS-PA** \| **6.78 (74.0%)** \| **14.16 (26.0%)** \| **9.91** \| \| **NiOx only** \| **12.62 (87.1%)** \| **32.02 (12.9%)** \| **24.07** \|   Note: The average lifetime can be calculated with the equation: 𝜏_𝑎𝑣𝑔_ = (𝐴_1_𝜏_1_^2^ + 𝐴_2_𝜏_2_^2^)/ (𝐴_1_𝜏_1_+𝐴_2_𝜏_2_), where parameters A_1_ and A_2_ are the amplitude fractions for each decay component, and 𝜏_1_ and 𝜏_2_ represent the decay coefficients of the fits.  **Table S4.** Best performance for devices with various SAMs deposited on NiOx   \| **Device** \| *J*_SC_/mA cm^-2^ \| *V*_OC_/V \| FF/% \| PCE/% \| \| --- \| --- \| --- \| --- \| --- \| \| **CDTS-MN (1)** \| 19.320 \| 0.623 \| 69.80 \| 8.41% \| \| **CDTS^b^-MN (1b)** \| 18.960 \| 0.625 \| 67.80 \| 8.03% \| \| **CDTS-CA (2)** \| 16.070 \| 0.452 \| 63.20 \| 4.59% \| \| **CDTS-PA (3)** \| 16.140 \| 0.441 \| 72.20 \| 5.15% \| \| **NiOx-only** \| 15.460 \| 0.413 \| 64.00 \| 4.08% \|   **Table S5.** Photovoltaic parameters of 15 **CDTS^b^-MN** devices fabricated under the same experimental conditions.   \| Device No. \| *J*_SC_/ mA cm^-2^ \| *V*_OC_/ V \| FF \| PCE/ % \| \| --- \| --- \| --- \| --- \| --- \| \| 1 \| 18.96 \| 0.625 \| 67.8 \| 8.03 \| \| 2 \| 20.1 \| 0.564 \| 70.8 \| 8.02 \| \| 3 \| 20.09 \| 0.586 \| 68.1 \| 8.01 \| \| 4 \| 19.89 \| 0.606 \| 66.2 \| 7.97 \| \| 5 \| 19.17 \| 0.614 \| 66.8 \| 7.86 \| \| 6 \| 19.58 \| 0.586 \| 68.4 \| 7.85 \| \| 7 \| 19.77 \| 0.58 \| 68.4 \| 7.84 \| \| 8 \| 19.67 \| 0.581 \| 68.4 \| 7.82 \| \| 9 \| 19.44 \| 0.611 \| 65.8 \| 7.82 \| \| 10 \| 19.88 \| 0.563 \| 69.3 \| 7.75 \| \| 11 \| 19.44 \| 0.601 \| 66.3 \| 7.74 \| \| 12 \| 20.18 \| 0.535 \| 71.6 \| 7.73 \| \| 13 \| 19.34 \| 0.548 \| 70.8 \| 7.51 \| \| 14 \| 19.49 \| 0.541 \| 70.9 \| 7.48 \| \| 15 \| 19.81 \| 0.57 \| 65.8 \| 7.42 \| \| Mean ± s.d. \| 19.65±0.35 \| 0.581±0.027 \| 68.4±2.0 \| 7.8±0.2 \|   **Table S6.** Photovoltaic parameters of 15 **CDTS-MN** devices fabricated under the same experimental conditions.   \| Device No. \| *J*_SC_/ mA cm^-2^ \| *V*_OC_/ V \| FF \| PCE/ % \| \| --- \| --- \| --- \| --- \| --- \| \| 1 \| 19.32 \| 0.623 \| 69.8 \| 8.41 \| \| 2 \| 20.07 \| 0.558 \| 74.2 \| 8.32 \| \| 3 \| 19.97 \| 0.576 \| 71.8 \| 8.26 \| \| 4 \| 19.62 \| 0.582 \| 72.4 \| 8.26 \| \| 5 \| 19.91 \| 0.594 \| 69.6 \| 8.24 \| \| 6 \| 20.05 \| 0.587 \| 69.5 \| 8.19 \| \| 7 \| 20.05 \| 0.585 \| 69.8 \| 8.19 \| \| 8 \| 19.27 \| 0.581 \| 72.6 \| 8.13 \| \| 9 \| 19.75 \| 0.607 \| 67.2 \| 8.05 \| \| 10 \| 20.12 \| 0.582 \| 68.6 \| 8.04 \| \| 11 \| 19.69 \| 0.614 \| 66 \| 7.98 \| \| 12 \| 19.67 \| 0.578 \| 70.1 \| 7.96 \| \| 13 \| 20.06 \| 0.594 \| 66.3 \| 7.9 \| \| 14 \| 19.41 \| 0.591 \| 68.6 \| 7.88 \| \| 15 \| 20.14 \| 0.583 \| 67.1 \| 7.88 \| \| Mean ± s.d. \| 19.81±0.30 \| 0.589±0.016 \| 69.6±2.4 \| 8.1±0.2 \|   **Table S7.** Photovoltaic parameters of 15 **CDTS-PA** devices fabricated under the same experimental conditions.   \| Device No. \| *J*_SC_/ mA cm^-2^ \| *V*_OC_/ V \| FF \| PCE/ % \| \| --- \| --- \| --- \| --- \| --- \| \| 1 \| 16.14 \| 0.441 \| 72.2 \| 5.15 \| \| 2 \| 14.84 \| 0.489 \| 70.9 \| 5.14 \| \| 3 \| 17.96 \| 0.425 \| 67.2 \| 5.13 \| \| 4 \| 17.69 \| 0.424 \| 67.9 \| 5.09 \| \| 5 \| 17.63 \| 0.424 \| 67.6 \| 5.06 \| \| 6 \| 15.6 \| 0.473 \| 68.1 \| 5.03 \| \| 7 \| 14.14 \| 0.492 \| 71.4 \| 4.97 \| \| 8 \| 15.11 \| 0.492 \| 66.4 \| 4.94 \| \| 9 \| 15.27 \| 0.478 \| 67.5 \| 4.93 \| \| 10 \| 15.1 \| 0.473 \| 68.4 \| 4.89 \| \| 11 \| 14.91 \| 0.476 \| 68.6 \| 4.87 \| \| 12 \| 14.32 \| 0.477 \| 70.4 \| 4.81 \| \| 13 \| 14.59 \| 0.462 \| 70.8 \| 4.77 \| \| 14 \| 13.66 \| 0.486 \| 70.9 \| 4.7 \| \| 15 \| 16.71 \| 0.425 \| 66.1 \| 4.69 \| \| Mean ± s.d. \| 15.58±1.36 \| 0.462±0.027 \| 69.0±2.0 \| 4.9±0.2 \|   **Table S8.** Photovoltaic parameters of 15 **CDTS-PA** devices fabricated under the same experimental conditions.   \| Device No. \| *J*_SC_/ mA cm^-2^ \| *V*_OC_/ V \| FF \| PCE/ % \| \| --- \| --- \| --- \| --- \| --- \| \| 1 \| 16.07 \| 0.452 \| 63.2 \| 4.59 \| \| 2 \| 15.95 \| 0.475 \| 60.4 \| 4.58 \| \| 3 \| 15.1 \| 0.477 \| 62.9 \| 4.54 \| \| 4 \| 12.99 \| 0.551 \| 63.1 \| 4.51 \| \| 5 \| 15.22 \| 0.471 \| 62.5 \| 4.48 \| \| 6 \| 12.95 \| 0.522 \| 65.7 \| 4.44 \| \| 7 \| 14.65 \| 0.487 \| 61.8 \| 4.41 \| \| 8 \| 14.88 \| 0.454 \| 64.7 \| 4.37 \| \| 9 \| 13.92 \| 0.507 \| 60.3 \| 4.26 \| \| 10 \| 16.47 \| 0.412 \| 62.6 \| 4.25 \| \| 11 \| 13.55 \| 0.479 \| 65.3 \| 4.24 \| \| 12 \| 16.57 \| 0.415 \| 61.6 \| 4.23 \| \| 13 \| 14.13 \| 0.446 \| 65.3 \| 4.11 \| \| 14 \| 12.41 \| 0.533 \| 61.9 \| 4.1 \| \| 15 \| 14.57 \| 0.448 \| 61.4 \| 4.01 \| \| Mean ± s.d. \| 14.61±1.31 \| 0.475±0.040 \| 62.8±1.7 \| 4.3±0.2 \| |
| --- | --- | --- | --- | --- | --- | --- | --- | --- | --- | --- | --- | --- | --- | --- | --- | --- | --- | --- | --- | --- | --- | --- | --- | --- | --- | --- | --- | --- | --- | --- | --- | --- | --- | --- | --- | --- | --- | --- | --- | --- | --- | --- | --- | --- | --- | --- | --- | --- | --- | --- | --- | --- | --- | --- | --- | --- | --- | --- | --- | --- | --- | --- | --- | --- | --- | --- | --- | --- | --- | --- | --- | --- | --- | --- | --- | --- | --- | --- | --- | --- | --- | --- | --- | --- | --- | --- | --- | --- | --- | --- | --- | --- | --- | --- | --- | --- | --- | --- | --- | --- | --- | --- | --- | --- | --- | --- | --- | --- | --- | --- | --- | --- | --- | --- | --- | --- | --- | --- | --- | --- | --- | --- | --- | --- | --- | --- | --- | --- | --- | --- | --- | --- | --- | --- | --- | --- | --- | --- | --- | --- | --- | --- | --- | --- | --- | --- | --- | --- | --- | --- | --- | --- | --- | --- | --- | --- | --- | --- | --- | --- | --- | --- | --- | --- | --- | --- | --- | --- | --- | --- | --- | --- | --- | --- | --- | --- | --- | --- | --- | --- | --- | --- | --- | --- | --- | --- | --- | --- | --- | --- | --- | --- | --- | --- | --- | --- | --- | --- | --- | --- | --- | --- | --- | --- | --- | --- | --- | --- | --- | --- | --- | --- | --- | --- | --- | --- | --- | --- | --- | --- | --- | --- | --- | --- | --- | --- | --- | --- | --- | --- | --- | --- | --- | --- | --- | --- | --- | --- | --- | --- | --- | --- | --- | --- | --- | --- | --- | --- | --- | --- | --- | --- | --- | --- | --- | --- | --- | --- | --- | --- | --- | --- | --- | --- | --- | --- | --- | --- | --- | --- | --- | --- | --- | --- | --- | --- | --- | --- | --- | --- | --- | --- | --- | --- | --- | --- | --- | --- | --- | --- | --- | --- | --- | --- | --- | --- | --- | --- | --- | --- | --- | --- | --- | --- | --- | --- | --- | --- | --- | --- | --- | --- | --- | --- | --- | --- | --- | --- | --- | --- | --- | --- | --- | --- | --- | --- | --- | --- | --- | --- | --- | --- | --- | --- | --- | --- | --- | --- | --- | --- | --- | --- | --- | --- | --- | --- | --- | --- | --- | --- | --- | --- | --- | --- | --- | --- | --- | --- | --- | --- | --- | --- | --- | --- | --- | --- | --- | --- | --- | --- | --- | --- | --- | --- | --- | --- | --- | --- | --- | --- | --- | --- | --- | --- | --- | --- | --- | --- | --- | --- | --- | --- | --- | --- | --- | --- | --- | --- | --- | --- | --- | --- | --- | --- | --- | --- | --- | --- | --- | --- | --- | --- | --- | --- | --- | --- | --- | --- | --- | --- | --- | --- | --- | --- | --- | --- | --- | --- | --- | --- | --- | --- | --- | --- | --- | --- | --- | --- | --- | --- | --- | --- | --- | --- | --- | --- | --- | --- | --- | --- | --- | --- | --- | --- | --- | --- | --- | --- | --- | --- | --- | --- | --- | --- | --- | --- | --- | --- | --- | --- | --- | --- | --- | --- | --- | --- | --- | --- | --- | --- | --- | --- | --- | --- | --- | --- | --- | --- | --- | --- | --- | --- | --- | --- | --- | --- | --- | --- | --- | --- | --- | --- | --- | --- | --- | --- | --- | --- | --- | --- | --- | --- | --- | --- | --- | --- |
|  |

**Reference**

[1] S. N. Afraj, C.-H. Kuan, J.-S. Lin, J.-S. Ni, A. Velusamy, M.-C. Chen, E. W.-G. Diau, *Adv. Funct. Mater.* **2023**, 33, 2213939.

[2] A. Abid, A. Velusamy, S. N. Afraj, W. Pervez, T.-Y. Su, S.-H. Hong, C.-L. Liu, M.-C. Chen, E. W.-G. Diau, *J. Mater. Chem. A* **2025**, 13, 9252.

[3] Y.-S. Shih, A. Velusamy, C.-H. Kuan, P.-Y. Huang, C.-H. Kuo, D.-Y. Zeng, C.-L. Liu, S.-H. Hong, X. Jiang, M.-C. Chen, E. W.-G. Diau, *Small* **2025**, 21, 2500642.

[4] M. Guo, C.-Y. Lin, S.-J. Liou, Y. J. Chang, Y. Li, J. Li, M. Wei, *J. Mater. Chem. A* **2021**, 9, 25086.

[5] T. Wu, Y. Wang, X. Li, Y. Wu, X. Meng, D. Cui, X. Yang, L. Han, *Adv. Energy Mater.* **2019**, 9, 1803766.
